# Emerging role of copper in the pathophysiology of spinal cord injury

**DOI:** 10.4103/NRR.NRR-D-24-01449

**Published:** 2025-07-05

**Authors:** Wenjing Ni, Peiling Qiu, Yang Huang, Sheng Wang, Xiaolei Zhang, Yifei Zhou, Di Zhang

**Affiliations:** 1 Department of Orthopedics, The Second Affiliated Hospital and Yuying Children’s Hospital of Wenzhou Medical University, Wenzhou, Zhejiang Province, China; 2 Zhejiang Provincial Key Laboratory of Orthopedics, Wenzhou, Zhejiang Province, China; 3 The Second School of Medicine, Wenzhou Medical University, Wenzhou, Zhejiang Province, China; 4 Department of Orthopedics, Taizhou Municipal Hospital, Taizhou, Zhejiang Province, China

**Keywords:** apoptosis, copper, cuproptosis, ferroptosis, inflammation, necroptosis, oxidative stress, pyroptosis, spinal cord injury, therapy

## Abstract

Copper is a trace element that plays an important role in neuronal development, maturation, and function. It also acts as a cofactor for various copper-binding proteins or serves as an active component of their structure. Acquired copper deficiency has been associated with numerous neurological diseases. Recent research has demonstrated that serum copper concentrations are elevated following spinal cord injury, similar to the elevated copper levels observed after ischemic insult in a rat model of myocardial infarction. This suggests that spinal cord damage may impair the effective utilization of copper due to local ischemia following spinal cord injury. Studies have shown that copper supplementation may form part of a therapeutic strategy for patients with spinal cord injury. It has been reported to promote T-cell differentiation and proliferation, reduce malondialdehyde levels, decrease myeloperoxidase activity and apoptotic cell numbers, and enhance superoxide dismutase activity and glutathione levels. Additionally, copper supplementation may stimulate the transcriptional activity of hypoxia-inducible factor and restore angiogenic capacity, thereby increasing capillary density. Furthermore, researchers have found that dihydrolipoamide dehydrogenase, an enzyme involved in inducing cuproptosis, can influence the immune microenvironment of spinal cord injury by promoting copper toxicity. This leads to increased peripheral M2 macrophage polarization and systemic immunosuppression. This led us to hypothesize that copper may influence three major pathological pathways after spinal cord injury, inflammation, oxidative stress, and cell death, which are critical targets for therapeutic intervention. On the one hand, copper deficiency can cause spinal cord tissue damage; on the other hand, elevated serum copper may induce copper toxicity, contributing to cell death. Therefore, in this review, we investigate the possible link between spinal cord injury and copper in the perspective of inflammation, oxidative stress, and cell death. Additionally, we review published studies on copper metabolism and explore potential therapeutic strategies by considering various sources and mechanisms of copper delivery.

## Introduction

Spinal cord injury (SCI) represents a major neuropathological condition that is frequently associated with neuropathic pain, deficits in sensorimotor functions and dysfunction of the autonomic system (Dietz and Fouad, 2014). Additionally, injury to the central nervous system (CNS) can disrupt the balance between the immune system and the CNS, leading to secondary immunodeficiency (CNS injury–induced immunodepression) and an enhanced risk of infection (Bietar et al., 2021a, b). These complications are among the primary causes of mortality during the post-acute phase of SCI. The functional deterioration post-SCI occurs mainly through inflammation, oxidative stress, and programmed cell death (PCD). The inflammatory response is complex, involving various cell types and cytokines; an exaggerated inflammatory response can further increase cellular mortality (Orr and Gensel, 2018). Modulating this response is a key therapeutic strategy. The balance between reactive oxygen species (ROS) and antioxidants is vital for normal cellular functionality, and inhibiting ROS may protect spinal cord tissue from oxidative stress and cell death (Zhou et al., 2024). PCD encompasses several mechanisms, including apoptosis, necroptosis, ferroptosis and pyroptosis. Cuproptosis, a newly identified form of PCD, has gained great interest due to its association with the aggregation of fatty acylated protein and proteotoxic stress from the excessive accumulation of copper. Various studies have reported the potential association between cuproptosis and diseases, including SCI. In addition, cuproptosis may represent a novel therapeutic target, as research has shown that regulating cuproptosis-related genes can facilitate the diagnosis of diseases, predict prognosis, and help with pathological staging.

Copper is a key component of neuronal development, maturation and functionality in the CNS (Schlief and Gitlin, 2006; An et al., 2022), including the maintenance of mitochondrial activity (Tsvetkov et al., 2022), the synthesis of neurotransmitters (D’Ambrosi and Rossi, 2015), resistance to oxidative stress (Zhong et al., 2022), and the maintenance of redox balance (Kardos et al., 2018). In addition, copper influences synaptic transmission and associated signaling pathways by modulating Ca^2+^ or zinc binding and by regulating the expression of metallothionein (MT) expression (D’Ambrosi and Rossi, 2015). Acquired copper deficiency has been linked to a range of neurological diseases, including isolated peripheral neuropathy, motor neuron disease, myopathy, cerebral demyelination, cognitive dysfunction and optic neuropathy (Jaiser and Winston, 2010). The increased application of gastric bypass for weight management has highlighted copper deficiency as a contributor to myeloneuropathy. Restructuring the gastrointestinal tract impairs copper absorption, leading to dorsal column dysfunction and sensory ataxia (Kirkland et al., 2022). This suggests that copper may potentially contribute to the physiology and pathophysiology of the CNS including the spinal cord. Nevertheless, there is limited research relating to the relationship between SCI and copper levels.

Recent studies have indicated that serum copper concentrations are increased after SCI (Salsabili et al., 2009; Heller et al., 2021), thus suggesting a potential link between copper and SCI. It is well established that copper ions are typically maintained at a low concentration in healthy organisms, with deficiency and excess potentially leading to cellular damage or even death (Kardos et al., 2018). However, the specific role of elevated copper levels in the context of SCI remains unclear. In the CNS, excessive copper is closely associated with neurotoxicity. For instance, Tindel et al. (2001) demonstrated that the implantation of bronze bullet fragments into the spinal cord of rabbits induced a substantial localized area of neural injury within the spinal cord. Similarly, the injection of 52 to 208 μg of copper in the form of an albumin complex, or as cupric sulfate, into the cerebrospinal fluid (CSF) of cats led to increased concentrations of this metal in the neural tissues along with catastrophic disturbances in neuronal function and rapid changes at the histological level (Vogel and Evans, 1961). Interestingly, copper supplementation following SCI was shown to reduce the levels of malondialdehyde (MDA), reduced myeloperoxidase activity, and diminished the number of apoptotic cells. In addition, copper supplementation enhanced superoxide dismutase (SOD) activity and increased glutathione (GSH) levels, potentially mitigating neurological damage (Tural et al., 2021). Moreover, copper supplementation was shown to have a beneficial effect on motor recovery by increasing T-cell differentiation and proliferative responses, and the levels of SOD and interleukin (IL)-1 (Garcia et al., 2022). Under ischemic conditions, copper supplementation also enhanced the transcriptional activity of hypoxia-inducible factor (HIF)-1 and restored angiogenic capacity, thus resulting in increased capillary density (He and James Kang, 2013); this may facilitate the restoration of blood supply after SCI. Thereby, copper is likely to play a major role in the pathophysiology of SCI and may contribute to neuroregeneration post-injury, particularly via mechanisms involving inflammation, oxidative stress and PCD. Therefore, it is important to maintain copper homeostasis after SCI, and appropriately managing excess copper while supplementing deficiencies may offer novel therapeutic strategies in the future.

In this review, we aim to consolidate existing knowledge regarding copper metabolism within the spinal cord and explore the potential relationship between copper levels and SCI through mechanisms such as inflammation, oxidative stress, and PCD. We also propose several biomarkers associated with copper homeostasis and cuproptosis as this may facilitate disease diagnosis and prognostic predictions. Certain biomarkers may further assist in determining the pathological staging of various conditions. In addition, drawing on prior research, we propose hypotheses regarding the potential sources of increased copper ions following SCI, as well as therapeutic strategies that may be informed by these insights.

## Search Strategy

Relevant literature was retrieved by an electronic search of the PubMed database from January 2014 to December 2024. The search strategy and selection criteria used the following keywords: SCI, copper, inflammation, oxidative stress, apoptosis, pyroptosis, necroptosis, ferroptosis, cuproptosis, autophagy, drug therapy, and CNS. We used various combinations of these search terms to comprehensively access the literature. Articles published in English and focusing on SCI or copper were included. Articles that did not focus on SCI or copper were excluded, as were those not published in English. Most of the selected literature (70% of all references) was published from January 2014 to December 2024.

## Main Pathological Events in Spinal Cord Injury

SCI can be classified into two phases: primary injury and secondary injury. The primary injury involves the mechanical contusion or extrusion that occurs at the moment of the injury, resulting in vertebral fractures and dislocations, as well as the presence of bone fragments and tears in the spinal ligaments (Anjum et al., 2020). Secondary injury involves a series of exacerbated responses to primary injury, including both mechanical and chemical damage (Ahuja et al., 2017; Alizadeh et al., 2019). Secondary injury is categorized into four phases: the acute (within 48 hours), subacute (48 hours to 14 days), intermediate (14 days to 6 months), and chronic (more than 6 months) stages (Shen et al., 2022) (**Table 1**).

**Table 1:** Main pathological events in various phases of spinal cord injury

Phase	Pathological event
**Primary injury**	Ertebral fractures and dislocations Bone fragments and tears
**Secondary injury**	
Acute phase (within 48 h)	Ionic deregulation Free radical formation Excitotoxicity Inflammation Apoptosis Necrosis Vasospasm Local ischemia Hemorrhage Axonal swelling Microglia activation
Subacute phase (48 h to 14 d)	Calcium dysregulation Necrosis Apoptosis Macrophage recruition Myelin debris clearance Astrocyte proliferation Scar production
Intermediate phase (14 d to 6 mon)	Axonal degeneration Formation of cystic cavitations
Chronic phase (more than 6 mon)	Maturation of glial scar

In the acute phase, the clinical manifestations include ionic deregulation, free radical formation, excitotoxicity, edema, inflammation, apoptosis, and necrosis. During this phase, the permeability of the blood–spinal cord barrier intensifies, leading to local ischemic infarction and hemorrhage, which aggravate axonal swelling and the death of neurons and oligodendrocytes. In addition, damage to the spinal cord exposes the injured site to many inflammatory cells, which increases the permeability of the BSCB, further intensifying inflammation and creating a detrimental feedback loop. The infiltration of immune cells at the injury site disrupts blood flow, resulting in vasospasm that can persist for up to 24 hours (Shen et al., 2022). Apoptosis in neurons and glial cells was detected in this phase. In white matter, oligodendrocyte apoptosis also occurs. Microglia begin to be activated to release inflammatory cytokines (Shi et al., 2021).

In the subacute phase, calcium dysregulation becomes one of the most noticeable losses of ionic homeostasis, which contributes to neuronal excitotoxicity, leading to damage to nucleic acids, proteins, and phospholipids and culminating in neurological dysfunction. During this phase, cellular necrosis is amplified, macrophages are recruited, astrocytes proliferate, and scars are produced. Additionally, the clearance of myelin debris through the phagocytic response promotes axonal growth (Anjum et al., 2020).

In the intermediate and chronic phases, the inflammatory response begins to diminish, accompanied by axonal degeneration, the formation of cystic cavitations, and the maturation of the glial scar, all of which impede axonal regeneration and cellular migration (Anjum et al., 2020).

## Role of Copper in the Physiology of the Spinal Cord

CNS contains a significant concentration of copper, ranking third in terms of copper content within the body. Only the liver serves as the body’s major organ for copper storage, and the kidneys exhibit higher copper concentrations than the CNS does (Bhattacharjee et al., 2020). These findings underscore the essential function of copper as an indispensable element in CNS progression. In addition, as a crucial catalytic cofactor for various physiological processes, copper is irreplaceable for proper development of the nervous system (Wen et al., 2021a). Systemic copper levels are regulated within a relatively narrow range to support normal biochemical functions. Dysregulation of copper levels, whether increased or decreased, can have detrimental consequences.

**Figure 1:**
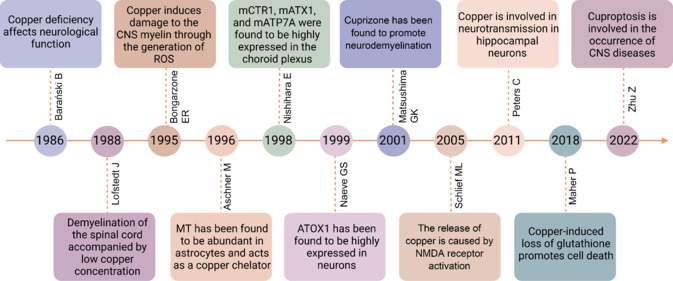
Timeline of the history and milestones of copper in the CNS. In 1986, decreased copper levels in the brain were associated with CNS dysfunction. In 1988, decreased copper concentrations and spinal demyelination were reported in goats with ataxia and paralysis. In 1995, it was reported that the ROS produced by copper can induce the destruction of myelin in the CNS. In 1996, MT was found to be abundantly expressed in astrocytes. In 1998, mCTR1, mATX1, and mATP7A were found to be highly expressed in the choroid plexus. In 1999, ATOX was found to be widely expressed in the brain, especially in neurons. In 2001, cuprizone, a copper chelator, was found to be used for CNS demyelination by inducing oligodendrocyte apoptosis. In 2005, the activation of NMDA promoted the transport of ATPase in the MD, which in turn promoted the release of copper ions. In 2011, copper released in the synaptic gap was shown to modulate neurotransmitter transmission. In 2018, the oxidative stress generated by GSH depletion was recognized as an important mechanism of copper-induced cell death. In 2022, cuproptosis was identified and shown to be widely involved in neurodegenerative diseases. Created with BioRender.com. ATOX: Antioxidant protein; ATP7A: ATPase 1; ATX1: antioxidant protein 1; CNS: central nervous system; CTR1: copper transporter 1; GSH: glutathione; MD: Menkes disease; MT: metallothionein; NMDA: N-methyl-D-aspartatic acid; ROS: reactive oxygen species.

The study of copper in the CNS has a long history. In 1986, decreased copper levels in the brain were associated with CNS dysfunction, suggesting that copper homeostasis in the brain is important for the maintenance of CNS function (Barański, 1986). In 1988, decreased copper concentrations and spinal demyelination were found in goats with ataxia and paralysis, further indicating the crucial role of copper in the CNS and its possible involvement in the generation of myelin (Lofstedt et al., 1988). In 1995, it was reported that the ROS produced by copper can induce the destruction of myelin in the CNS (Bongarzone et al., 1995). In 1996, MT, a chelator of copper ions, was abundantly expressed in astrocytes, suggesting that large amounts of copper may be present in astrocytes (Aschner, 1996). In 1998, *mCTR1* (copper transporter 1), *mATX1* (antioxidant protein 1), and *mATP7a* (copper transporter copper-transporting ATPase 1) were found to be highly expressed in the choroid plexus, suggesting that the choroid plexus functions in copper uptake and efflux in the brain (Nishihara et al., 1998). In 1999, antioxidant protein (ATOX), a copper ion transport protein, was found to be widely expressed in the brain, especially in neurons, suggesting an important role for copper in neurons (Naeve et al., 1999). In 2001, cuprizone, a copper chelator, was found to be used for CNS demyelination by inducing oligodendrocyte apoptosis, indirectly illustrating the important role of copper in oligodendrocyte myelin formation (Matsushima and Morell, 2001). In 2005, the activation of N-methyl-D-aspartic acid (NMDA) promoted the transport of ATPase in Menkes disease, which in turn promoted the release of copper ions, suggesting the possible involvement of copper in neuronal synaptic transmission (Schlief et al., 2005). Fortunately, in 2011, copper released in the synaptic gap was shown to modulate neurotransmitter transmission (Peters et al., 2011). In 2018, oxidative stress generated by GSH depletion was recognized as an important mechanism of copper-induced cell death, providing new insights into the mechanism of copper-induced cell death (Maher, 2018). In 2022, a new type of PCD, copper death, was identified and shown to be widely involved in neurodegenerative diseases, further confirming the important role of copper in the CNS (Zhu et al., 2024c; **Figure 1**).

Like those in the brain, copper is distributed unevenly within the spinal cord, as copper levels in the gray matter are two to three times greater than those in the white matter, particularly in the lateral gray matter (Que et al., 2008; Hilton et al., 2024). Posterior myelitis due to copper deficiency syndrome also confirms the distribution of copper in the spinal cord. The characteristic neurological manifestation of copper deficiency is ataxic myelopathy, which is characterized by an unsteady gait that combines sensory ataxia and spasticity and is related to dysfunctions of the posterior and lateral columns, respectively. This observation underscores the essential role of copper in the functioning of the lateral column (Mutti et al., 2021). The physiological implications of this heterogeneous distribution warrant further investigation. The average neural concentration is approximately 0.1 mM, while extracellular copper levels vary, with concentrations ranging from 10 to 25 µM in blood serum, 0.5 to 2.5 µM in CSF, and 30 µM in synaptic regions. Notably, intracellular copper concentrations are two to three orders of magnitude greater than those found extracellularly, with the highest levels observed in the cytosolic and mitochondrial fractions, a phenomenon that is associated with the intracellular transport of copper (Que et al., 2008; Gaier et al., 2013).

Research on the mechanisms of copper import and export within the spinal cord is limited. However, in the brain, copper enters the brain through capillary endothelial cells that form the blood–brain barrier (BBB) and the choroid plexus, which forms the blood‒CSF barrier (BCB). The BBB is believed to be the principal pathway through which copper penetrates the brain parenchyma, whereas BCB is likely involved in the regulation of copper homeostasis in the CSF (Choi and Zheng, 2009). Given that serum and CSF contain significantly higher concentrations of copper than brain tissues do, the BBB and BCB may serve to restrict the influx of copper into the brain (Lutsenko et al., 2019). Given the presence of analogous structures within the spinal cord, copper may also be conveyed to the spinal cord through the serum and CSF.

Recent findings indicate that copper transport proteins are expressed in the BBB and BCB, which further demonstrates their crucial role in copper transportation (Gromadzka et al., 2020). ATP7A, a critical protein involved in copper export, has been found in the endothelial cells of the choroid plexus and cerebrovascular endothelial cells that form the BBB (Choi and Zheng, 2009; Fu et al., 2014). Dysfunction of ATP7A in the BBB can disrupt copper entry, resulting in insufficient copper in brain tissue. This impairment of ATP7A function can cause progressive neurodegeneration and marked connective tissue dysfunction, which are the clinical manifestations of classic Menkes disease (Horn and Wittung-Stafshede, 2021). Conversely, ATPase copper transporting beta (ATP7B) is also expressed in the endothelial cells of the choroid plexus, albeit with functions distinct from those of ATP7A. In the brain, ATP7B mainly promotes copper efflux from cells. Consequently, a deficiency in ATP7B does not hinder copper transport into the brain; rather, it leads to a gradual accumulation of copper in brain tissues, ultimately resulting in cytotoxic effects (Gromadzka et al., 2020).

In brain tissues, copper ions are internalized primarily by cells through CTR1, which serves as the principal mechanism for copper acquisition in these tissues. In addition, CTR1 expression was found to be regulated by copper levels; it is downregulated in the presence of excess copper and upregulated under conditions of copper deficiency, suggesting the presence of a negative feedback mechanism governing CTR1 regulation (Wang et al., 2025). CTR1 is crucial for maintaining copper homeostasis. Additionally, divalent metal transporter 1 is capable of transporting copper and is abundantly expressed in neurons (Arredondo et al., 2003; Skjørringe et al., 2015). However, such dual transport is restricted to cell types, and further investigation is necessary to determine its role in regulating copper homeostasis within neurons.

Once copper enters cells, it can be distributed into various cellular compartments, contributing to the copper pool and facilitating the function of copper-dependent proteins (Zhu et al., 2024c). ATP7A and ATP7B also function as key regulators of copper homeostasis. At high intracellular copper levels, ATP7A and ATP7B export excess copper out of the cell through the formation of vesicles. ATP7A and ATP7B travel back from the plasma membrane to the trans-Golgi network (TGN) when intracellular copper levels return to basal levels (Bhattacharjee et al., 2020). In noradrenergic neurons, ATP7A is capable of facilitating the transport of copper into peptidyl-α-monooxygenase, dopamine-β-hydroxylase (DBH) and other copper-requiring enzymes for their function, thereby playing crucial roles in the regulation of copper homeostasis. Conversely, ATP7B is situated within vesicles and is responsible for maintaining copper equilibrium in the cytosolic environment (Gaier et al., 2014; Bonnemaison et al., 2016; Schmidt et al., 2018).

In addition to the aforementioned proteins related to copper transport across the membrane, copper homeostasis is also regulated by various other proteins. The first group includes proteins that bind and store copper ions, such as GSH and MTs (Maryon et al., 2013a; Chen et al., 2020a), which play crucial roles in mitigating copper-induced cellular damage. GSH facilitates the uptake of copper and regulates copper distribution between intracellular compartments during neuronal differentiation. The second type is copper ion chaperones, which can support the functions of related proteins. For example, the absence of cytochrome c oxidase 1 and 2 proteins (SCO1 and SCO2) results in diminished cellular copper ion levels (Dodani et al., 2011a). Copper chaperone for superoxide dismutase (CCS), one of the copper chaperones responsible for delivering copper to SOD, also operates through a negative feedback mechanism for the maintenance of copper homeostasis. Specifically, when the intracellular copper concentration is excessive, the inactivation of CCS is enhanced. In contrast, CCS expression is upregulated in response to low intracellular copper levels (Zhu et al., 2024c).

Copper plays different roles in different cells. Research has indicated that the concentration of copper in glial cells is significantly greater than that in neurons. Nevertheless, a comprehensive investigation into the precise distribution and concentration of copper across various cell types within the spinal cord has yet to be conducted and analyzed. Astrocytes, which are the predominant glial cells within the CNS, are physically located at the interface of the CNS blood supply and neurons, and they play important roles in copper acquisition, incorporation and export (Bhattacharjee et al., 2020). Furthermore, astrocytes are chiefly responsible for the regulation of copper levels. Research has shown a positive correlation between the copper concentration and the number of astrocytes present in the substantia nigra, globus pallidus, and striatum in murine models. This correlation may be attributed to the upregulation of MT and GSH levels following exposure to elevated copper levels (Bulcke and Dringen, 2016). This is the reason why astrocytes are often referred to as copper sponges. Nevertheless, research has indicated that the ratio of copper to astrocytes diminishes with increasing age in the murine brain (Ashraf et al., 2019). These findings provide insight into the mechanism underlying age-related neurodegeneration.

It has been demonstrated that astrocytes are capable of exporting copper to neurons. As ATP7A has been found in both the TGN and vesicles of astrocytes, this finding highlights the important role of ATP7A in copper export (Niciu et al., 2006). In addition, the observed accumulation of copper and its sequestration by MT in the astrocytes of macular mice with mutant *ATP7A* further confirmed the involvement of *ATP7A* in copper export (Arredondo et al., 2003). Additionally, researchers have investigated the possibility that astrocytes may participate in cuprizone-induced demyelination in oligodendrocytes within the context of a multiple sclerosis model. This phenomenon is mediated by the neurotrophin receptor TrkB, which has been shown to facilitate the proliferation of astrocytes *in vivo* and regulate the production of astrocytic factors that contribute to the loss of neurons and oligodendrocytes. Additionally, TrkB drives the expression of CTR1, promoting copper uptake by astrocytes. This process is followed by a gradual release of copper, which may lead to the degeneration of oligodendrocytes and the loss of myelin. These findings indicate the dysregulated uptake of circulating copper and its subsequent release by astrocytes in the CNS. This elevated TrkB is related mainly to neuroinflammation. Researchers have reported that the expression of copper transporters in the CNS is profoundly augmented during neuroinflammation. Thus, the increase in CTR1 mediated by TrkB may represent a compensatory response to elevated copper levels aimed at mitigating copper-induced toxicity. The redistribution of copper to various cell types may contribute to the loss of oligodendrocytes and myelin (Colombo et al., 2021). In the CNS, oligodendrocytes can facilitate axonal signaling through the myelination of axonal segments (Kuhn et al., 2019) and provide metabolic support to the axon. The depletion of oligodendrocytes can result in axonal damage. A recent study has indicated that the administration of cuprizone, a copper chelator, can induce demyelination, thereby underscoring the critical role of copper in the myelination process (Zirngibl et al., 2022).

In neurons, copper can serve as a crucial cofactor of neurotransmitters and enzymes, such as DBH and tyrosinase, to support neuronal maturation and physiology. In noradrenergic neurons, copper is integral to the regulation of the catecholamine balance, which encompasses dopamine, norepinephrine/noradrenaline, and epinephrine/adrenalin. The conversion of dopamine to norepinephrine occurs within secretory granules and is contingent upon the activity of DBH, which is modulated by copper availability. Disorders in copper metabolism can significantly influence catecholamine equilibrium; specifically, copper deficiency in neurons can impair DBH function, resulting in elevated dopamine levels and diminished norepinephrine levels (Lutsenko et al., 2019). In addition, copper plays an essential role in synaptic transmission. Copper is located in the synaptic vesicles from which it is released and can be released upon neuronal depolarization. Upon stimulation of the NMDA receptor (NMDAR), calcium ions enter the cell through the NMDAR channel, subsequently triggering the translocation of copper-ATP7A to synapses. This translocation facilitates the release of copper into the synaptic cleft, where it can inhibit NMDAR activation, thereby providing a protective mechanism for neurons against glutamatergic excitotoxicity. Copper can also be involved in glutamatergic transmission, promoting the development and synaptogenesis of neurons. The presence of copper in the synaptic cleft can modulate various neurotransmitter receptors, including ionotropic glutamate receptors, gamma-aminobutyric acid ionotropic receptors, and ionotropic purinergic receptors (D’Ambrosi and Rossi, 2015). Copper also participates in the modulation of signaling cascades activated by neurotrophic factors, which are orchestrated by kinases and phosphatases (Grubman and White, 2014). Copper can bind to brain-derived neurotrophic factor and nerve growth factor, thereby influencing their interactions with specific receptors, TrkB and TrkA, respectively. The binding of copper to brain-derived neurotrophic factor is associated with a reduction in the proliferation of SH-SY5Y neuroblastoma cells, whereas its interaction with nerve growth factor is linked to an increase in cell proliferation (Travaglia et al., 2011, 2012). Furthermore, copper can increase the activity of receptors by promoting the phosphorylation of TrkB through matrix metalloproteinases (MMPs), ultimately fostering synaptic growth (Grubman and White, 2014). Therefore, copper appears to influence multiple stages of signal transduction pathways initiated by trophic factors, underscoring its critical role in neuronal function.

## Role of Copper in Spinal Cord Injury

### Potential role of copper in trace element regulation after spinal cord injury

Iron is one of the essential nutrients and plays a key role in redox reactions, electron transport, oxygen transport, and energy metabolism. An excess or deficiency of iron will influence normal physiological processes. On one hand, an excess of iron leads to ferroptosis, a process that is accompanied by the accumulation of lipid hydroperoxides. On the other hand, iron deficiency affects the synthesis of hemoglobin, causing anemia with tissue damage (Li et al., 2023b). Following SCI, the level of iron increases significantly, thus leading to ferroptosis via the Fenton reaction. Moreover, compared to other cells and tissues, neurons and glial cells exhibit high oxidative metabolic activity, high ROS metabolites, and low antioxidant capacity, thus suggesting that neurons are more sensitive to ferroptosis (Tao et al., 2024). This explains why ferroptosis inhibitors represent a potential treatment for SCI. In addition to ferroptosis, iron is also found to be elevated in B cells after SCI; this induces pyroptosis in B cells via translocase of the outer membrane 20 (Tom20)/B cell leukemia/lymphoma-2 associated X apoptosis regulator (BAX)/caspase/gasdermin E pathway. Since a deficiency of B cells will lead to immunosuppression, the elevated levels of iron will aggravate the inflammation response after SCI (Wu et al., 2023). Although the disorder of copper and iron metabolism both occur after SCI, the interplay between these two processes remains inadequately understood. It has been reported that copper is closely related with iron. However, copper can influence the transportation of iron. For example, the activation of ceruloplasmin (CP), a principal copper-binding protein, can promote the release of iron from cells, leading to an increasing concentration of iron in the serum, while copper deficiency can block iron transport, leading to iron deficiency. Hephaestin (HEPH), a multi-copper iron oxidase which encoded by *HEPH* gene, also takes part in the transportation of iron from enterocytes. In addition, copper also takes part in the physiological functionality of iron. For example, copper serves as an essential component in cytochrome oxidase, affecting the role of iron in erythropoiesis. On the other hand, iron may also antagonize copper metabolism. Research indicates that high-dose iron supplementation can result in copper depletion. Additionally, the upregulation of HIF-2ɑ caused by a low iron state is known to induce the mRNA transcription of *CTR1* and *ATP7A* (Li et al., 2023b; Jiayi et al., 2024). As iron and copper levels are upregulated after SCI, we hypothesize that the elevation of copper after SCI is one the reasons for the increasing concentration of iron. Moreover, the potential regulation of copper metabolism by iron after SCI also needs to be investigated.

Zinc, an essential trace element, plays an essential part in the regulation of inflammation, as well as homeostasis, oxidative stress, cell cycle progression, DNA replication, DNA damage repair, apoptosis, and aging (Wen et al., 2021b). Zinc is closely linked to the growth and maturation of neurons and energy metabolism, and is involved in a variety of CNS diseases (Ge et al., 2021a). However, an excess of zinc will lead to excitotoxicity, inducing oxidative stress and impairing the generation of cellular energy (Wen et al., 2021b). Following SCI, the serum levels of zinc are reduced, while the levels of zinc in the spinal cord increase gradually. The reduced level of zinc after SCI deteriorates the inflammatory response which leads to the inhibition of axonal regeneration and the exacerbation of motor function. This is because zinc deficiency can upregulate nuclear translocation of nuclear factor kappa-light-chain-enhancer of activated B cells (NF-κB), thereby suppressing macrophage polarization to pro-inflammatory phenotypes via the damage-associated molecular pattern (DAMPs)/Toll-like receptor 4 (TLR4)/NF-κB pathway and the subsequent expression of pro-inflammatory cytokines. An increase in tumor necrosis factor alpha (TNF-ɑ) following NF-κB, zinc deficiency also leads to apoptosis (Kijima et al., 2023). Therefore, the acute zinc concentration in the serum can be used to predict the severity of SCI, while the serum zinc concentration 12 hours after SCI can accurately predict the functional prognosis (Wen et al., 2021b). Nowadays, zinc supplementation is regarded as a potential therapeutic strategy for the future treatment of SCI. Since zinc has antioxidative effects, zinc supplementation after SCI can upregulate glutathione peroxidase 4 (GPX4), SOD, and GSH via the nuclear factor erythroid 2-related factor (NRF)/heme oxygenase-1 pathway, alleviating lipid peroxides, MDA and ROS (Ge et al., 2021a). This inhibits ferroptosis in neurons and effectively reduces neural damage after SCI. Recently, zinc-organic framework-based aggregation-induced emission-active nanozymes (Zn@MOF-TPD) have been produced to alleviate oxidative stress and inflammation, and promote motor recovery. The zinc in these nanozymes upregulates MMP-9 by inactivating endopeptidases to promote scar formation, inhibit axonal growth, and delay functional recovery (Zheng et al., 2024). Copper has similar functions to zinc. Both of copper and zinc participate in neurotransmitter synthesis, myelination, and the prevention of ROS formation by acting as cofactors for various enzymes or functional proteins. Although it is widely considered that copper and zinc exert antagonistic functions since they compete for the same sites of many metal-binding proteins, research has found that sub-lethal concentrations of copper will exacerbate zinc-induce neurotoxicity in vascular dementia with senile dementia (Kawahara et al., 2022, 2024). However, whether there is a potential link between elevated copper and decreased zinc levels after SCI has yet to be determined.

Selenium is crucial for immune responses and neurological repair processes, and it also serves as an antioxidant that reduces oxidative stress. However, excess selenium can lead to neural apoptosis. It has been reported that selenium concentrations decrease after SCI, along with an increase in Se-binding protein 1 (SELENBP1). SELENBP1 is a poorly characterized parameter of Se metabolism, transport, and intracellular accumulation, but it plays a critical role in specific physiological functions, potentially during cell differentiation, protein degradation, intra-Golgi vesicular transport, cell motility and redox modulation. Due to its close association with the degree of neurological impairment in SCI, SELENBP1 has been proposed as a biomarker to predict the likelihood of neurological recovery (Seelig et al., 2021). Additionally, selenium has been widely explored as a treatment for SCI. For example, zeolitic imidazolate framework-8 (ZIF-8) capped selenium nanoparticles (SeNPs@ZIF-8) have been produced to scavenge ROS, exert anti-inflammatory properties, and promote the differentiation of macrophages into the M2 phenotypes. In addition, these nanoparticles can bind to ferrostatin 1 to further alleviate lipid perioxidation (Zhou et al., 2024). Only a limited body of information exits about the potential link between copper and selenium. However, it has been reported that copper is a required cofactor for the activation of SELENBP1, thus indicating that a copper deficiency will suppress the activation of SELSNBP1 (Philipp et al., 2023).

### Potential role of copper in the regulation of inflammation following spinal cord injury

The inflammatory response following SCI is a multifaceted process that involves various cell types and a multitude of inflammatory cytokines. The pathological consequences of inflammation in the context of SCI are closely linked to the temporal progression of the injury. Although inflammation has beneficial effects, including pathogen elimination and wound healing, extensive immune cell infiltration mainly results in neural degeneration (Garcia et al., 2016). These immune cells are guided to the lesion site from the periphery via cytokines and chemokines released by microglia, astrocytes, and peripherally derived macrophages present at the lesion site (Mortazavi et al., 2015). Overall, ischemia, oxidative damage, edema, and glutamate excitotoxicity after SCI promote the upregulation of cytokines/chemokines, which are produced by resident microglia, astrocytes, peripherally derived immune cells, and endothelial cells. These cytokines/chemokines then induce the migration and infiltration of monocytes, peripherally derived macrophages, and lymphocytes into the lesion site and can continue to produce additional inflammatory mediators and cytokines/chemokines, which aggravate immune infiltration and ultimately lead to cell death.

In the early hours post-SCI, a variety of factors contribute to further cellular dysfunction and death, including cell permeabilization, pro-apoptotic signaling and ischemia. In addition, the intracellular calcium dysregulation in neurons and glia induces mitochondrial dysfunction and subsequent cell death. During this phase, there is an upregulation in the release of pro-inflammatory cytokines, including IL-1β, TNF-α and IL-6, from microglia and astrocytes. The activation of microglia is induced by TLRs which are capable of recognizing the DAMPs released from damaged tissues. Although the number of microglia at the injury site and in the vicinity of the injury is decreased due to apoptosis and mechanical impact, these cells remain the primary source of inflammatory mediators. Microglia contain three distinct types of inflammasomes: the nucleotide-binding, oligomerization domain-like receptor family pyrin domain containing 3 (NLRP3), the neuronal apoptosis inhibitory protein (NAIP)/NLR family caspase activation and recruitment domain-containing protein 4 (NLRC4), and absent in melanoma 2 (AIM2). The NLRP3 inflammasome can be activated by ROS. This type of NLRP activation leads to the recruitment of apoptosis-associated speck-like protein (ASC). This protein can transform pro-caspase-1 to caspase-1, which promotes the generation of IL-1β and IL-18. These cytokines, in turn, upregulate IL-6, which plays a critical role in initiating inflammatory responses and serves as a potent recruiter of immune cells following SCI. Furthermore, IL-6 enhances vascular permeability, induces neuronal apoptosis, and plays an essential role in the differentiation of neural stem cells into astrocytes. Microglia also produce TNF-ɑ, which works together with IL-1β as well as IL-6 to induce inducible nitric oxide synthase (iNOS) via the NF-κB pathway. Subsequently, iNOS can produce a large amount of nitric oxide, which induces demyelination and apoptosis. In addition to microglia, these inflammatory mediators can also be released by macrophages, astroglia, and neurons. These pro-inflammatory cytokines are presumed to be the predominant players early in the injury timeline, and remain at high levels in the first week post-injury. Within the initial hours following injury, there is also an invasion of neutrophils at the injury site. The upregulation of thrombin, histamine, and ROS can stimulate endothelial cells to express P-selectin which facilitates the adhesion and migration of neutrophils. Subsequently, the neutrophils that enter the injury site can phagocytize cellular debris and summon macrophages to the injured tissue. From days 1 to 7 post-injury, there is a significant upregulation of chemokines, which recruit monocytes, T cells, and dendritic cells to the injury site (Orr and Gensel, 2018; Hellenbrand et al., 2021).

Macrophages are classified into several subsets, including M1 (classical activation), M2 (alternative activation), regulatory, tumor-associated, and myeloid-derived suppressor macrophages, among others (Tang et al., 2019; Pan et al., 2020). M1-like macrophages exhibit cytotoxic properties and are capable of eliminating adjacent cells and inhibiting cellular proliferation, thereby limiting their regenerative potential following SCI; M2-like macrophages can promote cell proliferation and tissue growth, which may be associated with functional recovery after SCI (Ding et al., 2021; Zhao et al., 2023). During the initial week following injury, the populations of M1 and M2 in the damaged tissues were relatively evenly distributed. However, there is a notable increase in the M1 population over time, with peak macrophage activation occurring between 7 and 14 days postinjury (Orr and Gensel, 2018; Hellenbrand et al., 2021). Activated macrophages can not only phagocytose cell debris but also clean myelin debris to prevent further axonal degeneration and provide a pro-axonal regeneration environment since myelin debris, which contains axonal growth inhibitors, prevents axonal regeneration and myelin regeneration (Van Broeckhoven et al., 2021). In addition, myelin debris can act as an inflammatory stimulator to induce complement receptor 3-dependent inflammatory responses via the focal adhesion kinase (FAK)/phosphatidylinositol 3-kinase (PI3K)/threonine kinase (Akt) signaling pathway (Sun et al., 2010).

Currently, several studies have confirmed the link between inflammation and copper. Rice et al. (2001) demonstrated that copper is a more proinflammatory metal than manganese (Mn), nickel (Ni), iron (Fe), and zinc (Zn) are in the context of inducing pulmonary inflammation in rats. In addition, copper has been shown to increase the expression of IL-1β, IL-6, and TNF-ɑ, as well as the expression of the nicotinamide adenine dinucleotide phosphate (NADPH) oxidase and GPx genes (Teles et al., 2011). In addition, the increased uptake of copper by macrophages can combat certain infectious agents related to the transportation of copper into phagosomes via ATP7A (White et al., 2009). In contrast, copper deficiency can decrease the secretion of IL-2 in T cells (Gombart et al., 2020). It can also affect the activity of neutrophils, natural killer cells, and monocytes. Collectively, these findings indicate that excessive copper accumulation at the injury site can lead to inflammation, underscoring the critical role of copper throughout various stages of the immune response. It is also widely recognized that exposure to copper enhances the production of ROS via a Fenton-like reaction in the presence of hydrogen peroxide (H_2_O_2_). Given that ROS are considered key initiators of the inflammation process in tissue (Sies and Jones, 2020), this may be a critical step in copper-induced inflammation.

This observed process may be attributed to the activation of the NLRP3 inflammasome, as evidenced by the increased levels of NLRP3 following exposure to CuCl_2_, whereas treatment with a copper chelator results in the suppression of NLRP3 (Dong et al., 2021). Although a study by Zhou et al. (2022) utilizing BV2 cells revealed that copper can induce microglia-mediated neuroinflammation through NF-κB activation mediated by ROS, another study suggested that NLRP3 may be the specific target of copper since copper chelators inhibit canonical NLRP3 but not AIM2, NLRC4, and NLRP1 inflammasomes or NF-κB–dependent priming (Deigendesch et al., 2018). In addition, copper enhances the effect of amyloid-β (Aβ) peptides on microglial activation in Alzheimer’s disease. The combination of copper and Aβ can promote the activation of microglia through NF-κB and mitochondria-derived ROS, which leads to the release of TNF-ɑ and nitric oxide. Notably, Aβ or copper alone induced neither the release of soluble neurotoxic factors by microglia nor microglia-mediated neurotoxicity (Yu et al., 2015). As Aβ is also elevated after SCI (Kobayashi et al., 2010), we speculate that the neuroinflammatory response caused by copper may involve Aβ.

**Figure 2:**
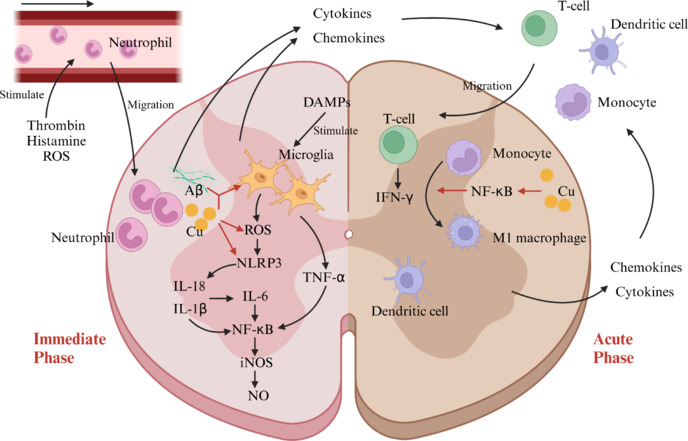
The role of copper in inflammation after SCI. The potential links between copper and inflammation can be categorized into the following categories: 1) copper may activate microglia via the activation of NLRP3 and ROS; 2) the combination of copper and Aβ can promote the activation of microglia through NF-κB and mitochondria-derived ROS; and 3) copper can regulate M1 polarization via the TLR4/NF-κB signaling pathway. Created with BioRender.com. Aβ: Amyloid-β peptide; DAMP: damage-associated molecular pattern; IL: interleukin; INF: interferon; iNOS: inducible nitric oxide synthase; NF-κB: nuclear factor kappa-light-chain-enhancer of activated B cells; NLRP3: nucleotide-binding, oligomerization domain-like receptor family pyrin domain containing 3; NO: nitric oxide; ROS: reactive oxygen species; SCI: spinal cord injury; TLR4: Toll-like receptor 4; TNF-α: tumor necrosis factor alpha.

Copper also participates in the activation of M1 polarization. Xiao et al. (2023) designed a microenvironment-responsive Cu^2+^-polydopamine network coating on an electrospun poly(ε-caprolactone) nanofibrous dressing that is capable of releasing copper into the acidic infection microenvironment. Researchers reported that the number of M1-positive cells increased after the application of copper, suggesting that M1 macrophage polarization can be induced by copper. Furthermore, the activation of M1 polarization may be related to the concentration of copper used since copper concentrations lower than 10 μM promote the expression of M2-related genes, whereas higher concentrations of Cu^2+^ (100 μM) stimulate proinflammatory marker expression (Díez-Tercero et al., 2021). Despite the substantial evidence supporting the involvement of copper in macrophage polarization, the precise mechanisms remain inadequately understood. According to previous studies, the process of M1 macrophage polarization is induced mainly by lipopolysaccharide and interferon, which induce macrophage polarization through the TLR4/NF-κB signaling pathway and Janus kinase (JAK)/signal transducer and activator of transcription (STAT) signaling pathway, respectively (Wang et al., 2021a). These findings lead to the hypothesis that copper may induce M1 polarization through these two pathways. Elevated copper can promote the expression of CTR1 and ATP7A, which demonstrates that elevated copper can induce the uptake of copper from the external environment into macrophages. This increases the expression of NF-κB and then facilitates M1 polarization (Huang et al., 2019b), which shows that copper can regulate M1 polarization via the TLR4/NF-κB signaling pathway. In addition, in the context of Wilson’s disease, excessive accumulation of the toxic metal copper in testicular tissue has been shown to stimulate the upregulation of inflammatory factors via the TLR4/NF-κB signaling pathway, which further corroborates the relationship between copper and M1 polarization. In addition, Solier et al. (2023) identified a pool of chemically reactive Cu^2+^ ions in the mitochondria of inflammatory monocyte-derived macrophages caused by CD44-mediated hyaluronate-bound metal uptake. The chemically reactive pool of copper in mitochondria characterizes the inflammatory state of macrophages. Therefore, CD44 may also take part in the induction of M1 polarization by elevated copper. The increased migration of neutrophils can also be observed after copper treatment (Hu et al., 2023). This result might be because copper can promote the deformability of neutrophils (Wang et al., 2021b; **Figure 2**).

Recently, MMP-3 was shown to mediate neuroinflammation after SCI. MMP-3 is inducibly expressed by damaged neurons, astrocytes, oligodendrocytes, microglia/macrophages, and vascular endothelial cells and subsequently activates various growth factors, cell adhesion molecules, cytokines, chemokines, and receptors other than the extracellular matrix. Dysregulated MMP-3 stimulates excessive inflammatory responses (Zhang et al., 2023b). Researchers have reported that the inflammation induced by Nano-CuO is associated mainly with the expression of MMP-3, while MMP-3 knockdown significantly alleviates acute and chronic pulmonary inflammation (Zhang et al., 2024). However, the mechanism of this process in SCI still needs further study.

Capturing copper is considered a new route for treating inflammatory disease. Solier et al. (2023) designed a metformin dimer that inactivates mitochondrial copper to oppose macrophage activation and dampen inflammation *in vivo*. Monoisoamyl 2,3-dimercaptosuccinic acid, a copper chelator, is also used to chelate copper to reduce inflammation. These findings suggest that copper-induced inflammation is a promising therapeutic target for SCI (Patwa et al., 2022).

In summary, elevated copper plays an important role in the development of inflammation. These processes include the activation of microglia, the promotion of M1 macrophage polarization, the facilitation of neutrophil migration, and the activation of monocytes, lymphocytes, and natural killer cells. In addition, copper can also act on inflammatory regulators to modulate the inflammatory response at the site of injury. These findings provide important clues for exploring the potential links between SCI and elevated copper.

### Potential role of copper in the regulation of oxidative stress following spinal cord injury

Oxidative stress is caused by an imbalance between the production of ROS and antioxidant defense when the body is stimulated by internal or external factors (Liu et al., 2025). Oxidative stress occurs at various phases of SCI. Previous studies have reported that markers of oxidative stress specific to lipid and protein oxidation increase after SCI (Liu et al., 2020; Ge et al., 2021b; Gao et al., 2023). The inhibition or scavenging of ROS production during SCI has also been demonstrated to contribute to neurological functional recovery after SCI (Shimizu et al., 2018; Zhou et al., 2018). The increased levels of ROS following SCI is mainly due to ischemia and the excessive production of free radicals (Scheijen et al., 2022). Here, we discuss the effect of copper on oxidative stress in SCI.

ROS is a molecule or molecular fragment that contains one or more unpaired electrons containing oxygen, including hydrogen peroxide, superoxide, singlet oxygen, and the hydroxyl radical (Herb and Schramm, 2021; Teleanu et al., 2022). The mechanism of action of transition metal ions participating in oxidative stress may involve the formation of hydroxyl radicals, superoxide ions and other forms of ROS (Tarin et al., 2023; Teschke and Eickhoff, 2024).

Copper mainly participates in the production of hydroxyl radical (OH). In the presence of bio-reducing agents, such as ascorbic acid or GSH, copper ions (Cu^2+^) can undergo reduction to cuprous ions (Cu^+^) via the Haber-Weiss reaction which catalyzes the generation of active ·OH via the Fenton reaction (Jomova et al., 2022). It is important to consider that the rate constant for the reaction of Cu^+^ with H_2_O_2_ is several orders of magnitude greater than that for Fe^2+^ (Halliwell and Gutteridge, 1985), as shown in Equations 1 and 2.

*O_2_^–^ + Cu^2+^ → O_2_ + Cu^+^      (1)

Cu^+^ + H_2_O_2_ → Cu^2+^ + ·OH + OH^–^      (2)

•OH is the most powerful oxidizing free radical that can be produced in biological systems (Teleanu et al., 2022; Yu et al., 2023) and can cause oxidative damage by extracting hydrogen from amino containing carbon to form carbon-centered protein radicals, and extracting hydrogen from unsaturated fatty acids to form lipid radicals (Stadtman, 1990; Gaetke and Chow, 2003). ·OH can also lead to various modifications of DNA bases, DNA strand breakage and changes in calcium and sulfhydryl homeostasis (Valko et al., 2006).

The effect of different concentrations of Cu^+^ on human erythrocytes and lymphocytes was investigated by Husain and Mahmood (2019) who demonstrated that the incubation of erythrocytes with copper chloride, a Cu^2+^ compound, enhanced the production of ROS and nitrogen in a Cu^2+^ concentration-dependent manner.

Under normal circumstances, the main source of free radicals in cells is the “leakage” of electrons from the electron transport chain in the mitochondria and endoplasmic reticulum into molecular oxygen, thus producing super-oxides (Cheeseman and Slater, 1993). The participation of transition metal ions such as copper can significantly enhance these autoxidation reactions (Cheeseman and Slater, 1993). Pourahmad and O’Brien (2000) previously compared the molecular cytotoxic mechanism of liver cell death induced by CuCl_2_ with cadmium chloride (CdCl_2_), an environmental toxin. These authors found that Cu^2+^-induced cytotoxicity was the result of mitochondrial ROS formation in a manner that was independent of the cytoplasmic ROS formation caused by the redox cycle.

Furthermore, the process of oxidative stress increases significantly during environmental stress, such as exposure to ultra violet, ionizing radiation, chemotherapeutics, inflammatory cytokines, environmental toxins or heat (Finkel and Holbrook, 2000). Gamma radiation from DNA solutions containing Cu cause changes in the conformation of DNA in oligonucleotides and natural and synthetic DNA. The changes in DNA conformation induced by ionizing radiation are significant because they depend on copper ion concentrations in a highly non-linear manner at low copper concentrations, which are not observed in the absence of copper ions (Trumbore et al., 2001). This means that the presence of copper ions in response to external stimuli will exacerbate the oxidative damage to DNA caused by ionizing radiation.

In order to cope with the harmful effects of ROS, the human body possesses multiple antioxidant systems. The first class of antioxidant defense systems developed against oxidative damage are those that prevent the occurrence of ROS, and those that block and trap the free radicals that are formed (Cheeseman and Slater, 1993). One form of preventative antioxidant defense is the removal of peroxides that react with transition metal ions to produce active free radicals. This includes the hydrogen peroxide and lipid hydroperoxides produced during the lipid peroxidation process. The role of catalase and GPx is to safely break down peroxides.

The second system of natural antioxidant defense is the repair process that removes damaged biomolecules before they accumulate and before their presence causes changes in either cell metabolism or vitality (Cheeseman and Slater, 1993). This system mainly consists of enzymatic and non-enzymatic antioxidants. Enzymatic antioxidants mainly include SOD, catalase and GPx, which can catalyze the disproportionation or reduction reaction of ROS, thereby reducing the concentration of ROS. Non-enzymatic antioxidants mainly include vitamin C, vitamin E, GSH and melatonin. These substances can react with ROS to remove its activity directly, or fight ROS damage by modulating signaling pathways.

Ossola et al. (1997) investigated the effects of Cu on oxidative stress in the liver and found that the administration of copper sulfate (CuSO_4_) may promote oxidative stress in the liver, as evidenced by a reduction in the activity of catalase and GPx, as well as increased SOD activity. Zhang et al. (2000) showed that Cu may suppress Cu-Zn SOD and GPx activities, and also resulted in high MDA levels in the liver. In human blood cells, Husain and Mahmood (2019) showed that the incubation of erythrocytes with copper chloride (Cu^2+^ compounds) decreased GSH and total thiol content, altered antioxidant enzyme activities and decreased antioxidant capacity, also in a manner that was dependent on Cu^2+^ concentration.

Several studies provide evidence to support that elevated Cu^2+^ concentrations may promote ROS-induced oxidative damage. For example, Cu has been reported to induce DNA strand breaks and base oxidation (Brezova et al., 2003). DNA-bound Cu^+^ predominantly mediates DNA base modifications, while unbound Cu^+^ mainly mediates the generation of apparent strand breaks (Drouin et al., 1996). Hydrazine was shown to induce DNA damage in the presence of Cu^2+^, while hydroxyl radical scavengers and SOD were unable to inhibit combined hydrazine and Cu^2+^ damage to DNA. The combination of hydrazine and Cu^2+^ frequently induces piperidine destabilization sites at thymine residues, especially GTC sequences. The incubation of human lymphocytes with Cu^2+^ has been shown to induce DNA damage, as determined by a sensitive comet assay (Husain and Mahmood, 2019).

Elevated copper levels can increase lipids oxidation products and disrupts the integrity and functionality of cell membranes. Elevated levels of mitochondrial lipid peroxidation products have been detected in a rat model of copper overload (Sokol et al., 1990). It is well-known that the oxidation of low-density lipoprotein (LDL) promotes atherosclerosis by enhancing macrophage-to-foam cell conversion and by contributing to vasoconstrictive and pro-thrombotic properties (Haidari et al., 2001). One of the most common techniques for initiating LDL oxidation *in vitro* is incubation with Cu^2+^. Cu is a powerful catalyst for LDL oxidation and may be involved in the oxidative modification of LDL into its atherosclerotic form (Steinberg, 1997). Free copper ions are not the only form responsible for LDL oxidation. CP, which contains seven copper atoms per molecule, can serve as a source of free copper (Harris, 1992) and is known to participate in LDL oxidation (Lynch and Frei, 1995; Mukhopadhyay and Fox, 1998). It is also considered that high-density lipoprotein may be more susceptible to Cu-induced oxidation than LDL. At low Cu concentrations, high-density lipoprotein is more sensitive to oxidation due to increased tocopherol-mediated peroxidation. At high Cu concentrations, high-density lipoprotein has a higher concentration of Cu bound to lipoprotein lipids, thus increasing its oxidizing capacity (Raveh et al., 2000).

**Figure 3:**
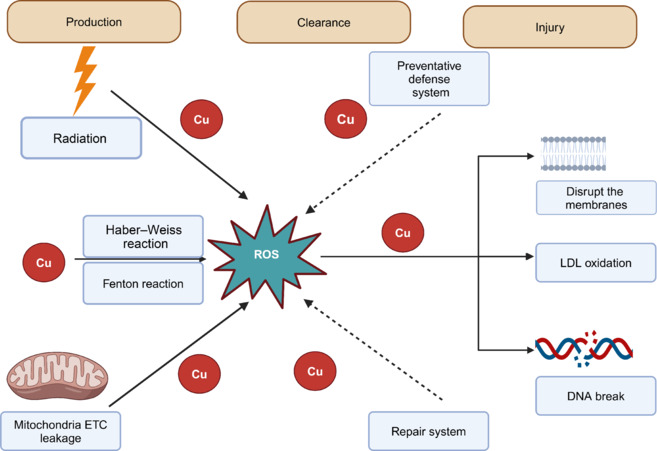
The role of copper in oxidative stress after SCI. Copper is involved in three processes—free radical generation, antioxidation, and oxidative damage—during oxidative stress. First, copper generates free radicals mainly through the Haber‒Weiss reaction and the Feton reaction. It can also promote ROS production processes such as ultraviolet irradiation and mitochondrial ETC leakage. Second, the antioxidant system can be weakened for preventative defense and the repair of two antioxidant systems. Finally, copper is involved in oxidative damage to DNA, the cell membrane and lipid oxidation. Created with BioRender.com. ETC: Electron transfer chain; LDL: low-density lipoprotein; ROS: reactive oxygen species; SCI: spinal cord injury.

An excess of Cu induces peroxidative damage to membrane lipids via reactions between oxygen and lipid radicals (Powell, 2000). In a previous study, Husain and Mahmood (2019) demonstrated that Cu induces lipid peroxidation in erythrocytes leading to erythrocyte membrane damage and the reduced activity of plasma membrane-bound enzymes, as determined by electron microscopy. Cu also causes lipid peroxidation in mitochondria, including the inner membrane (cristae) of liver cells (Britton, 1996). Cu also causes peroxidation of lysosomal membranes in hepatocytes (Sokol et al., 1990; Bremner, 1998; **Figure 3**).

The consequences of copper accumulation in Wilson’s disease are usually attributed to copper-induced oxygen radical-mediated damage (Sayre et al., 2000; White et al., 2001). Wilson’s disease is phenotypically variable, with predominantly hepatic or neurological manifestations. Patients with Wilson’s disease show evidence of morphological changes in the hepatic lysosome, an increase in lysosome brittleness, a reduction in membrane fluidity, alterations in the composition of membrane fatty acids and an increased lysosome pH value (Myers et al., 1993).

### Potential role of copper in the regulation of programmed cell death following spinal cord injury

Cell death is an important strategy for an organism to maintain intrinsic homeostasis, but can also be a pathological response of cells to changes in the external environment. Cell death was initially proposed by Vogt (1842). Later, Kerr et al. (1972) distinguished cell death into necrosis and apoptosis based on cell morphology. Necrosis is a passive form of cell death which is identified as swollen cells with swollen organelles, in which a cell ruptures and releases its contents to the extracellular environment, thus causing peripheral inflammation. In contrast, apoptosis is an active PCD which involves nuclear and cytoplasmic condensation, followed by cell fragmentation and phagocytosis by phagocytes (Fricker et al., 2018; Shi et al., 2021). Over recent years, cell death has been sub-divided further, and various forms of PCD have begun to appear in major research fields, thus enhancing our understanding of disease etiology and its mechanisms. PCD is a process of active cell death mediated by cell signaling under the activation, expression, and regulation of a series of genes, including autophagy, apoptosis, pyroptosis, ferroptosis and cuproptosis.

Numerous studies have proven that copper can induce cell death. As early as 1978, researchers found that normal cells died at medium copper concentrations > 30 μg/mL, thus suggesting that copper overload induces the onset of cell death (Chan et al., 1978). Since then, the potential mechanisms of copper overload have been revealed progressively, including ROS accumulation, proteasome inhibition and mitochondrial dysfunction (Chen et al., 2023). In addition to this, researchers have found that copper regulates the onset and progression of PCD via various pathways, reinforcing the important role of copper ions in cell death. Here, we try to elaborate the possible mechanistic link between copper and SCI in terms of copper-mediated PCD.

#### Cuproptosis

Copper plays an important role as a cofactor for several enzymes that influence growth, metabolism, and regulatory functions related to oxidative stress. The accumulation of copper also potentially contributes to a series of cellular dysfunctions and results in cell death.

Cuproptosis is a newly identified form of copper-dependent cell death and is considered to be highly correlated with mitochondrial respiration and lipoic acid pathways. Cuproptosis is distinct from other known cell death pathways due to the fact that it is not affected by the cleavage or activation of caspase-3 activity, BAX and B cell leukemia/lymphoma-2 antagonist/killer 1 (BAK1), pan-caspase inhibitors, or inhibitors of other known cell death mechanisms, including ferroptosis (ferrostatin-1), necroptosis (necrostatin-1) and oxidative stress (N-acetyl cysteine). Tsvetkov et al. (2022) identified the mechanism of cuproptosis, and proposed that copper toxicity was highly correlated with mitochondrial activity; this represented a key milestone in our understanding. There is growing concern regarding the potential health risks associated with exposure to the typical levels of copper found in everyday life, particularly through sources such as drinking water and food, with children being especially vulnerable. Furthermore, mutations in the ATPase, copper transporting, beta polypeptide gene (ATP7B) are also considered to be major risk factors for copper toxicity (de Romaña et al., 2011).

**Figure 4:**
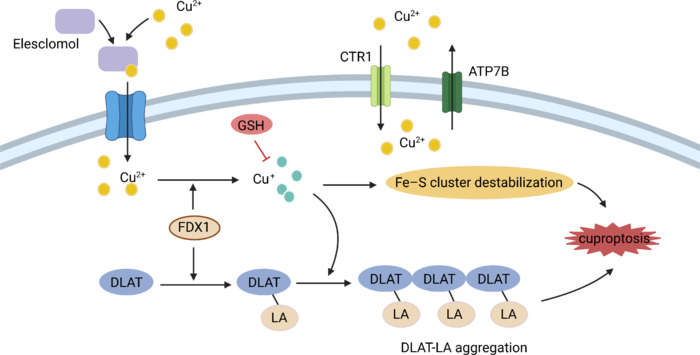
Signaling regulation of cuproptosis after SCI. In cuproptosis, elesclomol binds copper (Cu^2+^) in the extracellular environment and transports it through the plasma membrane. In addition, CTR1 can also transport copper into cells. In the intracellular compartment, Cu^2+^ is subsequently reduced to Cu^+^ via FDX1, which destabilizes Fe–S cluster proteins, ultimately leading to cell death. Additionally, copper binds to lipoyl moieties such as lipid-acylated DLATs in the TCA cycle, enhancing lipoylated protein aggregation and Fe–S cluster protein reduction, which triggers proteotoxic stress and cuproptosis. Created with BioRender.com. ATP7B: ATPase copper transporting beta; CTR1: copper transporter 1; DLAT: dihydrolipoamide S-acetyltransferase; FDX1: ferredoxin 1; Fe-S: iron-sulfur; LA: lipid acylation; SCI: spinal cord injury; TCA: tricarboxylic acid.

While copper chelators can save cells from cuproptosis, inhibitors of apoptosis, necroptosis, ROS-induced cell death or ferroptosis do not (Tsvetkov et al., 2022). Elesclomol (ES), a copper ionophore, binds copper (Cu^2+^) in the extracellular environment and transports it through the plasma membrane or mitochondrial membrane structure of a cell. Then, in the intracellular compartment, Cu^2+^ is reduced to Cu^+^ via cuproptosis-related gene ferredoxin 1 (*FDX1*), which is associated with ES sensitivity and causes destabilization of the iron-sulfur (Fe–S) cluster proteins, ultimately leading to cell death. However, the exact mechanism of how Fe–S cluster proteins trigger cuproptosis is still unclear. Furthermore, FDX1 is also an upstream regulator of protein lipoylation. The knock-out of either *FDX1* or lipoylation-related enzymes, such as mitochondrial enzyme dihydrolipoamide S-acetyltransferase (DLAT), can rescue cells from copper toxicity. The binding of copper to lipoyl moiety like lipid-acylated DLAT in the tricarboxylic acid (TCA) cycle enhances the aggregation of lipoylated protein and the reduction of iron-sulfur cluster protein, thus triggering proteotoxic stress and cell death. Protein lipoicylation is a post-translational modification known to occur only in metabolism-related molecules involved in the process of initiating the TCA cycle (Li et al., 2022a; Tang et al., 2022a; Tsvetkov et al., 2022). The overexpression of copper importers (*SLC31A1*) or the knockdown of copper exporters (*ATP7A/B*) has been shown to lead to excessive intracellular copper levels, disturbing normal copper homeostasis and resulting in cuproptosis (Xie et al., 2023). In addition, ES promotes the degradation of ATP7A, which leads to cell death due to copper retention and oxidative stress (Gao et al., 2021; **Figure 4**).

Research has shown that cuproptosis plays an important part in various diseases. Salsabili et al. (2019) found that the plasma level of copper was higher in male patients with SCI than in a healthy group, thus indicating that cuproptosis may play an important role in SCI (Salsabili et al., 2009). However, very few studies have investigated the relationship between cuproptosis and SCI. In patients with amyotrophic lateral sclerosis (ALS), the level of copper is significantly increased in spinal cord tissue, especially in the region most affected by disease. In the spinal cords of mice, an improvement in the amount of non-SOD1 copper was obviously related to disease development. In a recent study, DLD, a multifunctional oxidoreductase known to positively regulate cuproptosis, was suggested to influence the peripheral immune microenvironment and induce the polarization of M2 macrophages, further exacerbating systemic immunosuppression following SCI and adversely impacting the prognosis of SCI (Li et al., 2023a). Collectively, these studies provide evidence for the important role played by cuproptosis in the metabolic and oxidative processes of SCI.

#### Apoptosis

Apoptosis, the most common mechanism of PCD, mainly serves to eliminate superfluous cells as well as irreparably damaged cells and plays a crucial role in maintaining tissue homeostasis and integrity. In the early stage of apoptosis, cells show chromatin condensation, blistering of the plasma membrane, and generation of apoptotic bodies (Ketelut-Carneiro and Fitzgerald, 2022). Under electron microscopy, the structural integrity of the plasma membrane and the structural integrity of the organelles remain intact. By altering the normally asymmetrical distribution of phosphatidylserine across the plasma membrane, cells signal phagocytosis (Izadi et al., 2021). The pathways generated by apoptosis can be categorized into intrinsic and extrinsic pathways, which ultimately lead to the activation of cysteine-asparaginase. The extrinsic pathway is mediated by FasL (Fas ligand)/Fas (Fas cell surface death receptor), where Fas binds to FasL via the death domain (FADD) (Xue et al., 2023a). When procaspase-8 becomes hydrolyzed into active caspase-8, the recruited adaptor protein containing death-effector structural domains can interact with the death-effector structural domains of procaspases 8–10, thereby aggregating into death complexes (Ouyang et al., 2012). Activated caspase-8 can directly activate the effector caspase-3/7 or activate the BH3-only protein BAX-like BH3 protein (BID) into truncated BID (tBID) to indirectly induce cell death via a pathway that is intrinsic to apoptosis (Bedoui et al., 2020). The intrinsic pathway is dependent mainly on the release of proapoptotic factors from mitochondria, a mitochondrial pathway triggered by mitochondrial damage signaling, leading to cell death by promoting DNA fragmentation (Abbaszadeh et al., 2020). In this process, the B-cell leukemia/lymphoma-2 (BCL-2) protein family regulates the mitochondrial membrane potential. For example, the activation of its family members BAX and BAK1 oligomerization decreases the mitochondrial membrane potential and creates a pore in the outer mitochondrial membrane, which in turn leads to the release of cytochrome c (Xue et al., 2023a). The release of cytochrome c affects energy production in mitochondria on the one hand, and on the other hand, cytochrome c, which enters the cytoplasm, stimulates a cell-damaging protein proteolytic cascade reaction (Westphal et al., 2014). In the cytoplasm, cytochrome c recruits apoptotic protease-activating factor 1, promotes apoptotic protease-activating factor 1 oligomerization, and binds procaspase-9 to form apoptosomes (Ouyang et al., 2012). Then, caspase-9 is activated, which promotes the downstream proteolytic activation of caspase-3/7 and induces apoptosis (Bedoui et al., 2020).

Apoptosis plays a key role in secondary injury after SCI (Wang et al., 2019), especially the extensive apoptosis of neurons and oligodendroglia, which may be one of the causes of long-term neurological deficits after SCI (Shi et al., 2021). In addition, after SCI, activated astrocytes release C–C motif ligand 2, which acts on microglia and neuronal cells through the sEV pathway, causing the apoptosis of neuronal cells and the activation of microglia. In turn, activated microglia can release IL-1β, further aggravating neuroinflammation and neuronal apoptosis (Rong et al., 2021). The occurrence of apoptosis after SCI is associated with the activation of intrinsic and extrinsic pathways, accompanied by the activation of upstream and downstream components of the caspase-3 apoptotic pathway (Springer et al., 1999), decreased BCL-2 expression, elevated Bax expression, matrix swelling/rupture of the mitochondrial outer membrane, and inflammatory responses. P53, an important protein regulating the transcription of extrinsic (Fas) and intrinsic (BCL-2 proapoptotic family members), increases the translocation of BAX from the cytoplasm to the mitochondria, the release of cytochrome c and the expression of apoptotic protease activating factor 1, which is also significantly elevated after SCI (Abbaszadeh et al., 2020). However, whether copper is involved in the activation of apoptosis in these environments remains unclear.

**Figure 5:**
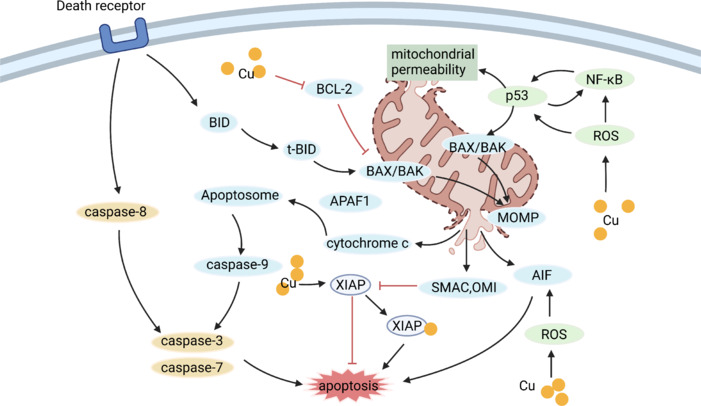
The role of copper in apoptosis after SCI. Copper can regulate apoptosis in the following ways: 1) Copper can induce the transportation of BCL-2 to mitochondria, leading to the release of cytochrome C and ultimately to apoptosis. 2) Excessive copper treatment elevates the level of ROS, which causes the release of AIF, leading to apoptosis through a caspase-independent pathway. 3) The NF-κB pathway and excessive ROS induced by excessive copper upregulate p53 levels, and activated p53 can induce NF-κB in turn. Upregulated p53 not only can interact with BAX to regulate apoptosis but can also directly induce mitochondrial permeability and apoptosis. 4) Copper can bind XIAP directly, which destabilizes XIAP, ultimately leading to apoptosis. Created with BioRender.com. AIF: Apoptosis-inducing factor; APAF1: apoptotic protease-activating factor 1; BAK: B-cell leukemia/lymphoma-2 antagonist/killer 1; BAX: B-cell leukemia/lymphoma-2 associated X apoptosis regulator; BCL-2: B-cell leukemia/lymphoma-2; BID: BAX-like BH3 protein; MOMP: mitochondrial outer membrane permeabilization; NF-κB: nuclear factor-κB; ROS: reactive oxygen species; SCI: spinal cord injury; SMAC: second mitochondrial-derived activator of caspases; t-BID: truncated BID; XIAP: X-linked inhibitor of apoptosis.

Currently, excessive copper has been shown to result in apoptosis since Bax is upregulated and BCL-2 is downregulated after copper treatment in splenocyte and thymocyte death, accompanied by changes in the mitochondrial transmembrane potential. These findings indicate that copper-induced apoptosis involves a mitochondrial caspase-dependent pathway (Mitra et al., 2013). Copper also induces BCL-2 translation to mitochondria in human neuroblastoma SH-SY5Y cells, which leads to the release of cytochrome C and ultimately to apoptosis (Lu et al., 2025). In addition, the increase in ROS following excessive copper treatment causes the release of apoptosis-inducing factor (AIF), which also leads to apoptosis through a caspase-independent pathway (Mitra et al., 2013). Recently, p53-induced apoptosis has been found in a variety of neurodegenerative disorders, such as Parkinson’s, Alzheimer’s, and Huntington’s diseases, and plays an important role in copper-mediated apoptosis (Levenson, 2005). Copper can activate both p53-dependent and p53-independent pathways. In the p53-dependent pathway, p53 can activate proapoptotic genes such as BAX. P53 can also interact with BAX and BCL-2 to regulate apoptosis. In addition, p53 can directly induce mitochondrial permeability and apoptosis, which is independent of proapoptotic genes. However, the role of p53 is influenced by the cell type, the presence of growth factors or oncogenes, the intensity of the stress signals, and the cellular level of p53 (Mitra et al., 2013). Thus, whether it plays the same role in SCI still needs further study (Levenson, 2005). Additionally, the NF-κB pathway and excessive ROS induced by excessive copper can also upregulate p53, and activated p53 induces NF-κB, which is involved in p53-induced apoptosis (Zhao et al., 2018). X-linked inhibitor of apoptosis (XIAP), which can inhibit apoptosis, can promote the ubiquitination and degradation of copper metabolism MURR domain 1 (COMMD1), inhibiting the efflux of copper from cells. Copper can bind XIAP directly, which destabilizes XIAP, ultimately leading to apoptosis (Mitra et al., 2013). A recent report in copper-treated spleens and thymuses also provided evidence that the increase in CD8^+^ T cells induced by copper can lead to apoptosis, possibly through the Fas signaling pathway (Mitra et al., 2013). In addition, some researchers have also utilized nanocopper to observe apoptosis in podocytes and reported that nanocopper can affect the oxidative‒antioxidant balance, leading to increased generation of ROS and MDA, resulting in cytotoxicity and the induction of apoptosis. In contrast, pretreatment with the ROS scavenger N-(2-mercaptopropionyl)-glycine inhibited the podocyte apoptosis induced by nanocopper (Xu et al., 2012; **Figure 5**).

These findings highlight the essential role of copper in apoptosis, but the specific mechanisms of copper-induced apoptosis in SCI remain unknown because of limited research. Therefore, further investigations are needed to explore the role of elevated copper post-SCI.

#### Pyroptosis

Pyroptosis is an inflammatory form of cell death triggered by microbial infections or host factors (Zheng et al., 2025). The process of pyroptosis is initiated by activation of the inflammasome, which further activates a different set of caspases as compared to apoptosis, for example, caspase-1/4/5 in humans and caspase-11 in mice (Burdette et al., 2021). Activated caspases then activate gasdermin D (GSDMD), which may form pores in the membranes of cells and promote the release of various DAMP molecules from the cell, such as high migration rate group protein B1, ATP and DNA (Al Mamun et al., 2021) and also release cytokines such as IL-1β and IL-18 (Evavold et al., 2018; Xiao et al., 2018).

Current evidence suggests that pyroptosis plays a critical role in SCI and represents a potential novel therapeutic target for SCI therapy. Xu et al. (2021) demonstrated that the expression of NLRP3/GSDMD was significantly higher in samples from SCI patients compared to the normal blood samples. These authors also reported that all inflammasome-associated genes were detectable in the SCI group and that the transcript levels of IL-1β, IL-18, caspase-1, GSDMD, NLRP3, and AIM2, were all upregulated. Furthermore, there is evidence to suggest that the expression of pyroptosis-related genes peaked on day 7 after SCI with a marked increase in the levels of GSDMD protein. Xiong et al. (2022) also demonstrated that the microglia is the main cell type in which pyroptosis occurred. Recently, researchers and clinicians have paid significant attention to the possibility of treating SCI by interfering with the pathway of pyroptosis. Li et al. (2024a) reported that by inhibiting the pathway of NLRP3/caspase-1/IL-1β, the secretome of dental pulp stem cells were able to minimize pyroptosis in the microglia. Researchers have also been able to inhibit pyroptosis by inhibiting NLRP3 (Liu et al., 2024b).

**Figure 6:**
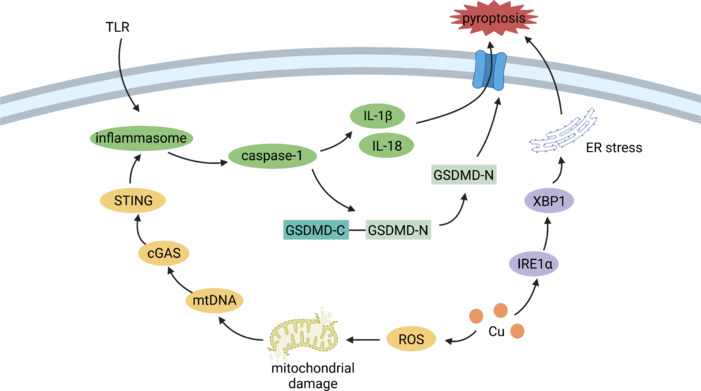
The role of copper in pyroptosis after SCI. Copper can regulate pyroptosis in the following ways: 1) Copper overload leads to excessive ROS, which causes mitochondrial damage and mtDNA cytoplasmic leakage. The accumulation of cytosolic mtDNA then leads to an increase in IL-1β and IL-18 through the cGAS-STING-NLRP3 pathway, ultimately causing pyroptosis. 2) Excess copper induces ER stress via the IRE1α/XBP1 pathway and ultimately contributes to pyroptosis. Created with BioRender.com. cGAS: Cyclic GMP-AMP synthase; ER: endoplasmic reticulum; GSDMD: activated gasdermin D; IL: interleukin; IRE1α: inositol-requiring protein 1α; mtDNA: mitochondrial DNA; NLRP3: nucleotide-binding, oligomerization domain-like receptor family pyrin domain containing 3; ROS: reactive oxygen species; SCI: spinal cord injury; STING: stimulator of interferon genes; XBP1: X-box-binding protein 1.

Numerous studies have proven the potential relationship between copper and pyroptosis. For example, the excessive uptake of copper can lead to pyroptosis in the spleen (Quan et al., 2025). Copper is also used to inhibit tumor growth and metastasis because it is able to induce pyroptosis by producing O_2_^–^· and highly toxic ·OH in cells (Zhang et al., 2023e; Zhu et al., 2024a). In astrocytes, an overload of copper can increase the expression of IL-1β and inflammatory factors, accompanied by the elevation of GSDMD-N and cleaved caspase-1, ultimately leading to pyroptosis. Furthermore, researchers have shown that Mito-Tempo can inhibit the generation of ROS and significantly alleviate copper-induced pyroptosis (Shi et al., 2024). This is because copper overload can lead to excessive ROS which causes mitochondrial damage and the cytoplasmic leakage of mitochondrial DNA (mtDNA). The accumulation of cytosolic mtDNA acts as a DAMP or pathogen-associated molecular pattern, leading to the increase of IL-1β and IL-18 via the cyclic GMP-AMP synthase (cGAS)-stimulator of interferon genes (STING)-NLRP3 pathway, finally inducing pyroptosis (Shi et al., 2024). Therefore, ROS may be a contributor to copper-induced pyroptosis. Furthermore, copper-induced pyroptosis may also act via the endoplasmic reticulum stress pathway. It has been reported that an elevation in the intake of copper can increase the mRNA levels of endoplasmic reticulum stress related genes and protein levels via the inositol-requiring protein 1ɑ (IRE1ɑ)/X-box-binding protein 1 (XBP1) pathway, and ultimately contribute to pyroptosis (Liao et al., 2022). In Alzheimer’s disease, copper-induced pyroptosis is known to be related to the promotion of Aβ accumulation via the NLRP3/Caspase-1/GSDMD pathway (Zhu et al., 2024b; **Figure 6**). However, it is still unclear whether copper can cause pyroptosis in SCI.

#### Ferroptosis

Ferroptosis is an iron-dependent mode of cell death characterized by lipid peroxidation and iron accumulation accompanied by morphological changes such as mitochondrial atrophy and increased membrane density (Feng et al., 2024). Under physiological conditions, Fe^3+^ in normal blood enters the cell via iron transferrin receptor 1 on the cell membrane. Then, Fe^3+^ is degraded to highly reactive Fe^2+^ in the presence of six-transmembrane epithelial antigen of prostate 3 (STEAP3) (Chen et al., 2020c). The transport of Fe^2+^ in the cell into the iron pool is mediated by divalent metal transporter protein (DMT1), with most of the Fe^2+^ bound to ferritin and stored intracellularly, and the remainder of the Fe^2+^ is pumped out of the cell bound to transport proteins (Hu et al., 2021). When intracellular iron is overloaded, the oversynthesis of Fe^2+^ triggers the Fenton reaction, which induces lipid peroxidation and ultimately ferroptosis. In particular, intracellular lipid peroxidation is mediated mainly by long-chain acetyl-coenzyme A synthetase long chain family member 4 (ACSL4), lysophosphatidylcholine acyl-transferase 3 (LPCTA3), and lipoxygenase (ALOX), which catalyze the formation of peroxides from polyunsaturated fatty acids, resulting in damage to lipid bilayers and affecting membrane function. In particular, free polyunsaturated fatty acids must be activated or incorporated into phospholipids for ferroptosis to occur (Chen et al., 2020c). Ferroptosis is also regulated by GPX4. Solute carrier family 3 member 2 (SLC3A2) and solute carrier family 7 member 11 (SLC7A11) together form the glutamate/cystine reverse transport system Xc^–^ on the cell membrane, which facilitates transmembrane translocation of cystine into the cells, which serves as a substrate for glutathione synthesis. GSH, in turn, maintains the activity and expression of GPX4 and converts lipid peroxides into nontoxic lipohydrols to inhibit ferroptosis (Hu et al., 2021). In addition, the mevalonate (MVA) pathway also plays an essential role in ferroptosis regulation. MVA generated in the cholesterol production pathway can further form isopentyl pyrophosphate, which produces farnesyl pyrophosphate. Isopentyl pyrophosphate and farnesyl pyrophosphate can bypass the cholesterol synthesis pathway and produce noncholesterol products, such as coenzyme Q10 (CoQ10), by the action of pyrophosphate synthase to scavenge free radicals. In a subsequent study, it was found to eliminate lipid peroxidation since CoQ10 can be converted into the lipophilic antioxidant CoQ10-H_2_ by the action of ferroptosis suppressor protein 1. CoQ10-H_2_ can remove phospholipid hydroperoxides (PL-OOH), thereby terminating the lipid oxidation chain reaction and inhibiting ferroptosis (Chen et al., 2020c).

Research has demonstrated that ferroptosis is one of the causes of severe outcomes secondary to injury after SCI. Yao et al. (2019) reported that the inhibition of ferroptosis with deferoxamine increased neuronal survival after SCI, inhibited glial cell proliferation, and promoted the recovery of hindlimb function. Ge et al. (2022b) also utilized ferritin-1 to scavenge hydrogen peroxide free radicals, which promoted myelin sheath formation and reduced white matter damage after SCI. This inhibitor also inhibited the proliferation of reactive astrocytes and activation of microglia and promoted the survival of oligodendrocyte progenitor cells, resulting in neuroprotection.

**Figure 7:**
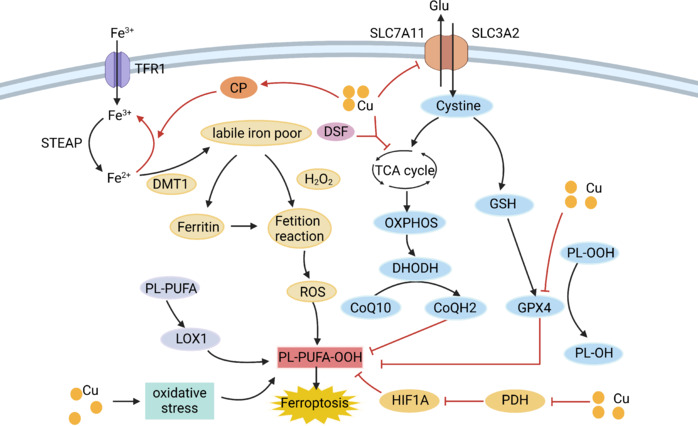
The role of copper in ferroptosis after SCI. Copper can regulate ferroptosis in the following ways: 1) Copper can induce autophagy-dependent GPX4 degradation and promote ferroptosis; 2) DSF/Cu can disrupt mitochondrial homeostasis, trigger lipid peroxidation, and ultimately lead to ferroptosis; 3) copper accumulation increases the concentration of CP, which converts Fe^2+^ divalent iron to Fe^3+^, thus inhibiting lipid peroxidation and ferroptosis; 4) copper promotes the stabilization of HIF-1A by inhibiting PHD and increasing the expression of lipid metabolism-related genes, leading to ferroptosis suppression; and 5) copper can induce oxidative stress via the Fenton reaction, which induces lipid peroxidation and ultimately ferroptosis. Created with BioRender.com. CoQ: Coenzyme Q; CP: ceruloplasmin; DHODH: dihydroorotate dehydrogenase; DMT1: divalent metal transporter protein; DSF: disulfiram; GPX4: glutathione peroxidase 4; GSH: glutathione; H_2_O_2_: hydrogen peroxide; HIF: hypoxia inducible factor; LOX: lipoxygenases; OXPHOS: oxidative phosphorylation; PHD: prolyl-4-hydroxylase domain; PL-OH: hydroxyl polyethylene glycol-modified liposomes; PL-OOH: phospholipid hydroperoxides; PL-PUFA: phospholipid-bound polyunsaturated fatty acids; PL-PUFA-OOH: lipid peroxides; ROS: reactive oxygen species; SCI: spinal cord injury; SLC3A2: solute carrier family 3 member 2; SLC7A11: solute carrier family 7 member 11; STEAP: six-transmembrane epithelial antigen of prostate; TCA: tricarboxylic acid; TFR1: iron transferrin receptor 1.

Various studies have demonstrated that copper can influence the development of ferroptosis through multiple pathways. For example, copper can induce autophagy-dependent GPX4 degradation and thus promote ferroptosis (Xue et al., 2023b). Gao et al. (2021) reported that the copper chelator ES can lead to the degradation of ATP7A, which in turn leads to an increase in the intracellular concentration of copper ions, causing the accumulation of ROS, which in turn promotes the degradation of solute carrier family 7 member 11, thereby promoting ferroptosis. In addition, disulfiram (DSF)/Cu can disrupt mitochondrial homeostasis, trigger lipid peroxidation, and ultimately lead to ferroptosis (Ren et al., 2021). In addition, excess copper induces oxidative stress through the Fenton reaction, which causes lipid peroxidation (Jiayi et al., 2024). However, copper may inhibit ferroptosis to some extent. In a study on copper and antiradiation therapy in hepatocellular carcinoma, researchers reported that copper accumulation could increase the concentration of CP, which converts Fe^2+^ divalent iron to Fe^3+^, thus inhibiting lipid peroxidation and ferroptosis and significantly increasing the expression of CP after SCI (Wu et al., 2018; Yang et al., 2022). Copper also promotes the stabilization of HIF-1A by inhibiting the prolyl-4-hydroxylase domain, and HIF-1A can upregulate lipid metabolism-related genes, leading to ferroptosis suppression (Xue et al., 2023a). This dual mechanism of copper in ferroptosis may involve the concentration of copper, the cell type or the cellular sensitivity to copper and iron (**Figure 7**). However, the regulation of ferroptosis by elevated copper after SCI remains unknown.

#### Autophagy

Autophagy is the main intracellular degradation system that degrades substances in the cytoplasm through lysosomes; autophagic substrates can be endogenous, such as damaged mitochondria or nuclear fragments, or exogenous, such as viruses or bacteria (Galluzzi et al., 2017; Ko and Chen, 2025). The degradation of substances during cellular damage may help maintain cellular homeostasis; thus, autophagy is believed to play a protective role in tissue damage repair.

Lysosomal function in neurons has been reported to be impaired on day 1 after SCI, as lysosomes are essential for the degradation function of autophagy, and impaired lysosomal function implies that autophagy may not function normally in SCI (Zhou et al., 2020b). The blockage of the autophagic pathway may be due to the accumulation of ROS, elevated levels of BCL-2 proapoptotic proteins, and decreased levels of 70-kDa heat shock protein chaperone protein after SCI, resulting in lysosomal damage, which in turn causes endoplasmic reticulum stress and ultimately cell death. It is now known that the copper ion concentration increases after SCI; thus, we speculate that the production of ROS and lipid peroxides during the process of autophagic flux blockade may be related to the increase in the copper ion concentration.

The activation of autophagic pathways after SCI may be related to the regulation of the central PI3K/Akt/mammalian target of rapamycin (mTOR)/adenosine monophosphate-activated protein kinase (AMPK) signaling pathway. In one study, after SCI, the AMPK-mTOR and AMPK/S-phase kinase-associated protein 2 (SKP2)/coactivator-associated arginine methyltransferase 1 (CARM1) signaling pathways were activated, which in turn activated transcription factor EB and transcription factor E3 to mitigate autophagy flux disruption, reduce endoplasmic reticulum stress, and promote the recovery of function after SCI (Zhou et al., 2020b). CuSO_4_ can induce cellular autophagy dependent on the Akt/AMPK/mTOR pathway by inducing the downregulation of p-Akt (Ser473)/Akt, p-mTOR/mTOR, and p-ULK1 (Ser757)/unc-51-like autophagy activating kinase 1 (ULK1) and subsequently the upregulation of phosphorylated AMP-activated protein kinase alpha (p-AMPKα)/AMPKα and p-ULK1 (Ser555)/ULK1. Among the abovementioned proteins, AMPK is a key energy sensor that maintains cellular homeostasis by regulating cellular metabolism. Under glucose starvation, AMPK inhibits the activation of the mammalian target of rapamycin complex 1, thereby inhibiting the phosphorylation of Ser757, which in turn interacts with the mammalian autophagy-initiating kinase ULK1 and promotes autophagy by directly activating ULK1 through the phosphorylation of Ser317 and Ser777 of ULK777. In contrast, mTOR, a central cell-growth regulator that integrates growth factors and nutrient signals, plays an inhibitory role in autophagy. Under nutrient-sufficient conditions, high mTOR activity is increased, and Ulk1 activation is inhibited by disrupting the interaction between ULK1 and AMPK through the phosphorylation of ULK1 at Ser757 (Kim et al., 2011). Therefore, we speculate that the occurrence of autophagy induced by the central PI3K/Akt/mTOR/AMPK signaling pathway after SCI may be associated with elevated concentrations of copper ions.

Furthermore, in mechanical SCI, activated JNK promotes the phosphorylation of BCL-2 at several sites (S70, S87, and T69) and contributes to the release of Beclin-1, which plays an important role in autophagy (Abbaszadeh et al., 2020). In one study, copper usage was shown to increase JNK activation and Beclin-1 expression by promoting ROS overexpression in cells (Trejo-Solís et al., 2012). Therefore, we speculate that copper can influence autophagy through the Jun NH2-terminal kinase 1 (JNK1)/BCL-2/Beclin-1 signaling pathway.

As previously noted, excessive intracellular copper not only triggers cuproptosis but also facilitates apoptosis, necrosis, pyroptosis, and ferroptosis. This phenomenon is considered a significant intersection between cuproptosis and other forms of PCD. Furthermore, we propose that cuproptosis may influence apoptosis, necrosis, pyroptosis, and ferroptosis through alternative pathways while also being subject to regulation by these other forms of cell death. Given that mitochondria, autophagy, and GSH are involved in the mechanisms underlying all these types of cell death, they may serve as critical hubs linking cuproptosis with other cell death pathways.

### Crosstalk between cuproptosis and other forms of cell death

#### Mitochondria

Mitochondria play a pivotal role in cellular energy production, along with a range of physiological and pathological processes. The mitochondria also participate in the biosynthesis of lipids, amino acids and nucleic acids, the generation of ATP, inflammation and cell death (Zong et al., 2016). Since PCD involves mitochondrial energy metabolism processes, the targeting of mitochondria is considered as a potential treatment with which to regulate cell death. It is also thought that these pathways of cell death do not operate in isolation, but rather there is crosstalk between cell death modalities, thus establishing a coordinated network with mitochondria as a central hub. Therefore, by influencing the functionality of mitochondria, cuproptosis may involve potential crosstalk with other forms of cell death.

Mitochondria play a central role in the initiation of apoptotic cell death, a process that is predominantly driven by mitochondrial outer membrane permeabilization (MOMP). In apoptotic cells, MOMP is stimulated by the pro-apoptotic effectors BAK and BAX, which accumulate in the mitochondria during mitochondrial apoptosis and induce the release of cytochrome c, second mitochondria-derived activators of caspases (SMAC), and Omi (Wei et al., 2001; Bock and Tait, 2020). In contrast, healthy cells are protected by anti-apoptotic BCL-2 proteins, which inhibit MOMP by binding to and neutralizing activated BAX and BAK (Gaumer et al., 2000; Suraweera et al., 2022). In apoptotic cells, the release of cytochrome c leads to the activation of caspase-9, ultimately resulting in apoptosis (Suraweera et al., 2022; Vringer and Tait, 2023). This is also accompanied by substantial remodeling of the mitochondrial crista (Harrington et al., 2023). Furthermore, the release of SMAC and Omi can also promote the activation of caspase-9 by inhibiting XIAP, a protein that blocks caspase-9 activation (Winkler et al., 2013). Numerous studies have reported that pro-apoptosis can influence mitofusin-1 and mitofusin-2, proteins known to be involved in mitochondrial fission and fusion. This is why apoptosis leads to mitochondrial fission (Brooks et al., 2007). Conversely, dynamin-related protein 1 is also a direct activator of BAX (Jenner et al., 2022). Mitochondrial fission can promote minority MOMP, which is triggered by non-lethal apoptotic stress, such as DNA damage, hypoxia or high glycolytic rates, while fusion can suppress it, thus suggesting that dysfunctional mitochondria can also signal death (Glover et al., 2024). MOMP is also reported to be associated with inflammation under conditions of caspase-9 deficiency (Vringer and Tait, 2023). Ca^2+^ also participates in the regulation of apoptosis as Ca^2+^ homeostasis determines cell susceptibility to apoptotic stimuli (Hajnóczky et al., 2006; Morciano et al., 2016).

Previous researchers applied enforced mitophagy to deplete mitochondria to investigate necroptosis and found that the forced activation of receptor-interacting protein kinase 3 (RIPK3) by chemically induced dimerization also induced the activation of mixed lineage kinase domain-like protein (MLKL), known as the executioner mechanism of necroptosis; these findings suggested that the onset of necroptosis may not be dependent on mitochondria (Bock and Tait, 2020). However, mitochondrial swelling and disruption were observed in TNF-induced and RIPK3-mediated cell death, thus suggesting a potential link between mitochondria and necroptosis (Glover et al., 2024). It has been reported that the ROS produced by mitochondria can promote the activation of receptor-interacting protein kinase 1 (PIPK1), which further leads to the formation of the necrosome. In contrast, activated PIPK3 can increase ROS generation in a feed-forward manner. In addition, the ROS released by damaged mitochondria can promote necroptosis. RIPK3 also engages with the mitochondrial pyruvate dehydrogenase complex in a manner that is dependent on MLKL and is reported to stimulate mitochondrial ROS production to promote necroptosis (Yang et al., 2018). Collectively, these findings indicate that the dysfunction of mitochondria can influence the process of necroptosis.

Pyroptosis is a pro-inflammatory lytic form of PCD. Little is known about the relationship between pyroptosis and mitochondria. Nevertheless, extensive crosstalk exists between pyroptosis and mitochondrial apoptosis. For example, MOMP can be upregulated by GSDMD, a key executor that triggers pyroptosis and can eventually lead to apoptosis by activating caspase-3 (Bock and Tait, 2020). GSDMA can also impair oxidative phosphorylation and elicit the release of mitochondrial proteins and mtDNA to induce inflammatory responses by targeting mitochondria and ultimately accelerating pyroptosis (Miao et al., 2023). The release of caspase-1 can induce mitochondrial apoptosis (Tsuchiya et al., 2019). In addition, mitochondrial apoptosis can also influence NLRP3 which leads to pyroptosis via caspase-1 (Bock and Tait, 2020).

In ferroptosis, the morphological alterations in the mitochondria include a reduced volume, increased density of the bilayer membrane, rupture of the outer mitochondrial membrane, and the disappearance of mitochondrial cristae. The dysfunction of mitochondria caused by ferroptosis can lead to reduced ATP synthesis, DNA damage, and the reduced synthesis of heme. Ferroptosis can occur via three major pathways, iron accumulation, lipid peroxidation (LPO), and cystine depletion. As mitochondria is a major contributor to the intracellular production of ROS, excessive ROS is one of the routes that can cause LPO, thus indicating the vital role of mitochondria in ferroptosis. Glutaminolysis, which serves as an anaplerotic pathway for the mitochondrial TCA cycle, can disrupt the pathway of cysteine deprivation-induced ferroptosis, leading to LPO. Dihydroorotate dehydrogenase is a free radical-scavenging antioxidant that plays a vital role in the TCA cycle and can reduce ubiquinone to ubiquinol and inhibit the production of lipid peroxides (Liu and Chen, 2024). This suggests that disruption of the TCA cycle could influence the progression of ferroptosis.

Copper plays a key role in mitochondrial respiration, enzyme activity, Fe metabolism and the maintenance of protein function. Research has shown that diseases or pathological conditions resulting from copper metabolism tend to exhibit a close association with mitochondrial metabolism, thus indicating a relationship between mitochondria and cuproptosis. In cuproptosis, the morphological alterations in mitochondria include shrinkage and membrane rupture. Cuproptosis leads to the upregulation of copper uptake into mitochondria. Excessive copper can bind directly to the thiooctylation component of the mitochondrial TCA cycle, leading to the abnormal aggregation of dihydrolipoamide DLAT and the destabilization of Fe-S cluster proteins which induce dysfunctionality in the mitochondria and eventually cell death. Researchers have found that the use of ferroptosis inducers (sorafenib and erastin) can enhance cuproptosis in primary liver cancer cells. However, these inducers played different roles. In ferroptosis, sorafenib and erastin can inhibit system Xc^–^ which influences the import of cystine, leading to the reduction of GSH and LPO. Sorafenib and erastin can cause cuproptosis by upregulating protein lipoylation, a process related to the degradation of FDX1 protein (Tian et al., 2023).

Given the important role of mitochondria in various types of programmed cell death, we can hypothesize that the mitochondrial damage induced by other PCDs can influence the development of cuproptosis. Similarly, the accumulation of lipoylated proteins and the destabilization of Fe-S cluster proteins in cuproptosis can affect the energy metabolism of mitochondria, which can then influence PCDs. Thus, mitochondria can serve as an important hub for cuproptosis and other PCDs.

#### Autophagy

Autophagy plays an important role in physiological and pathological processes and is regarded as an indispensable biological function by eliminating damaged organelles and macromolecules. A number of studies have shown that autophagy is involved in the regulation of PCD. Dysfunctional mitochondria can also trigger autophagy. This suggests that autophagy may represent a crosstalk mechanism between cuproptosis and other forms of PCD.

Several studies have confirmed the interplay between autophagy and apoptosis. Apoptosis and autophagy can be stimulated by the same inducers, such as ROS, ceramide, an elevation of cytosolic Ca^2+^, p19 alternative reading frame (p19ARF), p53, BH3-only proteins and members of the death-associated protein kinases family. At low levels of exposure, autophagy is induced to maintain cellular homeostasis and suppress apoptosis by turning over depolarizing or damaged mitochondria, thus precluding intrinsic apoptosis. However, beyond a specific threshold, autophagy further promotes apoptosis to protect cells (Maiuri et al., 2007; Harrington et al., 2023). Some of the proteins which play an essential part in autophagy have been reported to regulate apoptosis. BAX interacting factor-1 serves as a multifunctional protein that not only activates BAX to promote apoptosis, but also participates in the formation of autophagosomes. In neuronal cells, the expression of BAX interacting factor-1 can facilitate autophagy and inhibit apoptosis (Zhu et al., 2024c). In addition, Beclin 1, which is also a key regulatory protein of the autophagic pathway, interacts with the pathway of apoptosis. Following the inhibition of Beclin 1, the amount of apoptotic cells is increased, thus confirming the transformation from autophagy to apoptosis. The overexpression of Beclin 1 can induce apoptosis by downregulating BCL-2 and upregulating caspase-9 and caspase-3. This suggests that the upregulation or downregulation of autophagy can both facilitate apoptosis (Prerna and Dubey, 2022) and that this can be caused by the transformation from autophagy to apoptosis. Studies have investigated how this form of transformation might operate. A previous study showed that autophagy can induce apoptosis via cathepsin D (CTSD), a lysosomal aspartic protease of the pepsin superfamily. The maturation of CTSD can be suppressed by blocking autophagy via 3-methyladenine and inhibiting the expression of Atg1, Atg4, Atg5, Atg7, Atg14 and Syx17 which are essential for the formation and maturation of the autophagosome, thus suggesting the crucial role of autophagy in the maturation of CTSD. Subsequently, the mature form of CTSD is released into the cytosol to play a role in pro-apoptosis by stimulating BID (Di et al., 2021).

Autophagy can be activated in necrosis as an adaptive response to ATP depletion to replenish energy reserves to help counteract the necrotic process. However, the relationship between autophagy and necroptosis is complex. In fact, autophagy can exert a dual effect on necrosis. On the one hand, autophagy can inhibit the progression of necroptosis and prevent cells from necrotic death. The inhibition of autophagy promotes the process of necroptosis by triggering a bioenergetic crisis (Long and Ryan, 2012). This may be related to the degradation of pro-necroptotic factors induced by autophagy. Furthermore, Beclin-1 also acts as a target to inhibit the oligomerization of MLKL, thus leading to the suppression of necroptosis (Seo et al., 2020). On the other hand, research has demonstrated that autophagy can lead to necroptosis. Goodall et al. (2016) reported that the autophagy machinery can facilitate the activation of the necrosome leading to the phosphorylation of MLKL to cause cells to undergo necroptosis. This process requires p62, but this is independent of the light chain 3-interacting region (LIR) domain that binds light chain 3 (LC3). In addition, cellular inhibitor of apoptosis protein 1/2 (cIAP1/2), the E3 ligases that block RIPK1 from forming complex IIa with FADD, and caspase-8 can also be diminished by autophagy to promote necroptosis (Hou et al., 2010). The assembly of necrosomes also requires autophagosomal membrane protein ATG5 to provide docking sites. This dual role is also evident in terms of the effect of necrosis on autophagy (Zhang et al., 2023c). Necroptosis has also been reported to promote autophagy, as ROS generated during necroptosis may cause the induction of autophagy, the process that degrades damaged organelles and proteins (Gong et al., 2019). In ischemia-induced neuronal and astrocytic cell death, RIPK1 has been reported to take part in activating the autophagic-lysosomal pathway. However, RIPK1 can inhibit autophagy by activating NF-κB. MLKL can also disrupt the integrity of autolysosome membranes to block autophagy (Zhang et al., 2023c).

Mitophagy can help to regulate the process of pyroptosis. NLRP3 and caspase-1, the key regulators of pyroptosis, play important roles in regulating the balance between mitophagy and pyroptosis. NOD-like receptor family member of NLRP3 has been shown to bind with LC3 to induce mitophagy. In addition, NLRP3 and caspase-1 can induce mitochondrial damage, thus leading to PRKN-dependent mitophagy (Su et al., 2023). The activation of NLRP3 may be caused by the release of cathepsin B induced by autophagy (Wan et al., 2023). This conversion of pyroptosis to mitophagy may represent a compensatory pathway to clear pyroptotic cells via mitophagy.

Ferroptosis is now regarded as an autophagy-dependent form of cell death as autophagy is involved in seven processes of ferroptosis, as follows. (1) Autophagy can degrade ferritin which leads to the release of irons (Gao et al., 2016; Hou et al., 2016). The accumulation of irons is one of the sources of ferroptosis. The knockdown of nuclear receptor coactivator 4 (*NCOA4*) or autophagy-related genes (*ATGs*), which are the key targets of autophagy, can suppress the erastin-induced degradation of ferritin (Mancias et al., 2014; Hou et al., 2016). (2) Autophagy can also manifest as lipophagy. By degrading intracellular lipid droplets, the LPO in ferroptosis can be upregulated (Liu and Czaja, 2013). (3) Beclin 1, which can influence the process of autophagy, can inhibit system Xc^–^ to restrain the generation of GPX4 and ultimately cause LPO (Song et al., 2018b). (4) Dysfunctional lysosomes can also contribute to ferroptosis (Gao et al., 2016; Torii et al., 2016; Zhou et al., 2020a). (5) Sequestosome 1-mediated autophagy has been shown to inhibit SLC40A1 which plays a role in the importation of iron, thus leading to an excess intracellular iron and ultimately ferroptosis. (6) Copper can induce ferroptosis via tax1-binding protein 1 (TAX1BP1)-induced autophagy. TAX1BP1, which serves as a receptor of autophagy, can promote the degeneration of GPX4. The knock of *TAX1BP1* can block copper-induced ferroptosis and also diminish cell sensitivity to ferroptosis (Xue et al., 2023b). (7) Clockophagy, the selective degradation of the core circadian clock protein aryl hydrocarbon receptor nuclear translocator-like (ARNTL) by autophagy, can also promote ferroptosis. ARNTL has also been shown to suppress the transcription of *Egln2* and activate the transcription of hypoxia-inducible factor 1A to restrain ferroptosis. Thus, the autophagy of ARNTL can upregulate ferroptosis (Yang et al., 2019b).

Cuproptosis can also trigger autophagy. An intracellular excess of copper can regulate the AMPK-mTOR pathway to induce autophagy by upregulating the expression of autophagy-related genes such as ATG5, sequestosome 1, and microtubule-associated protein 1 light chain 3 (Yang et al., 2021). Interestingly, reducing intracellular copper by inhibiting SLC31A1 can also lead to autophagy in pancreatic cancer cells (Yu et al., 2019), although this requires further investigation. FDX1 also exhibits a positive relationship with autophagy marker genes (ATG5, ATG12 and Beclin-1), thus suggesting potential crosstalk between cuproptosis and autophagy (Lu et al., 2022). Furthermore, autophagy was inhibited in hepatocellular carcinoma cells by high expression levels of DLAT (Liu and Chen, 2024).

#### Glutathione

GSH as a ubiquitous tripeptide can maintain cellular homeostasis via multiple mechanisms. The most well-known role of GSH is its antioxidant ability to diminish ROS and RNS (Georgiou-Siafis and Tsiftsoglou, 2023; Lapenna, 2023). Furthermore, GSH can also combine with unstable cysteine and toxic electrophilic compounds.

GSH is regarded as a common hub for cuproptosis and ferroptosis. However, GSH acts in different ways. In cuproptosis, GSH can combine with excessive copper which can alleviate the negative impact of the aggregation of lipoylated proteins. GSH can also be imported into cells by SLC25A39. The blockade of SLC25A39 can diminish the transportation of GSH which leads to cells being sensitive to cuproptosis (Liu et al., 2024a). During ferroptosis, GSH, serving as an antioxidant, can upregulate the expression of GPX4 to reduce LPO. Thus, GSH can inhibicuproptosis and ferroptosis, thus validating its essential role in the interplay between cuproptosis and ferroptosis (Liu and Chen, 2024).

GSH can also regulate other forms of cell death as an antioxidant. For example, copper-induced apoptosis was previously associated with a reduction in GSH (Yang et al., 2019a). This may have been caused by elevated ROS levels following the depletion of GSH which subsequently induced the activation of intrinsic apoptosis (Sun et al., 2021). The elevation of ROS has also been shown to activate the NLRP3 inflammasome in glaucoma, leading to the release of IL-1β and the induction of pyroptosis. In this study, researchers also found that the inhibition of necroptosis via the RIP1/RIP3/MLKL pathway following the administration of GSK872 and Nec-1 led to a reduction in ROS production, thus demonstrating the relationship between necroptosis and ROS (Liu et al., 2022a). In glioma cells, shikonin-induced necroptosis can be inhibited by MnTBAP, which is known to scavenge mitochondrial superoxide. In contrast, necroptosis was enhanced in glioma cells following the administration of rotenone to increase ROS. The generation of ROS was regulated by RIP1 or RIP3; RIP1 upregulated ROS by targeting the NADPH oxidase 1 NOX1 and the small GTPase RAC1. On the other hand, the elevated levels of ROS after RIP3 was caused by activation of the mitochondrial protein GLUD1 (Lu et al., 2017). In tumor cells, pyroptosis induced by the depletion of GSH could be used as a new form of immunotherapy for cancer. These researchers designed a smart tumor microenvironmental ROS/GSH dual-responsive nano-prodrug (M-chlorophenylpiperazine) with high loading levels of paclitaxel and the photosensitizer purpurin 18. This nano-prodrug was shown to cause the depletion of GSH and enhance the activity of ROS, thus leading to GSDMD-induced pyroptosis (Xiao et al., 2021).

Overall, GSH has different roles in cuproptosis and other PCDs. In cuproptosis, GSH acts primarily as a copper chelator, whereas in other PCDs, GSH acts primarily as an antioxidant. Although copper-induced ROS are not necessarily required for cuproptosis, they can influence other forms of PCD. The excessive levels of ROS caused by other PCDs can also lead to the depletion of GSH, which in turn affects cuproptosis.

## Biomarkers Related to Cuproptosis and Copper Homeostasis

Currently, a novel study has revealed the relationship between the expression of cuproptosis-related genes and acute SCI (ASCI). DLD and MTF1 are significantly upregulated in ASCI patients, whereas glutaminase (GLS), lipoic acid synthetase (LIAS), LIPT1, and FDX1 are significantly downregulated in ASCI patients. DLD and MTF1 are likely the key genes involved in the effects of cuproptosis on ASCI because their expression is significantly upregulated in ASCI. In addition, the study also revealed that DLD, LIAS, and GLS are independent risk factors for ASCI and can be used to diagnose ASCI accurately. Among these cuproptosis-related genes, DLD is the only gene to be regarded as a potential biomarker in ASCI because of its essential role in the development of ASCI, and it can also be used to predict the neurological function of ASCI patients and assist with diagnosis and treatment (Li et al., 2023a). In addition to ASCI, cuproptosis-related genes also serve as biomarkers in different diseases. There are a few common biomarkers associated with cuproptosis.

FDX1 is a key enzyme that regulates cuproptosis by influencing the process of protein lipoylation. In addition, it also participates in iron‒sulfur protein synthesis as a mitochondria-associated protein. It encodes a small iron‒sulfur protein that participates in mitochondrial cytochrome reduction, Fe‒S cluster biosynthesis, and the synthesis of various steroid hormones (Sheftel et al., 2010). Recent studies have shown that FDX1 may serve as a prognostic marker for glioma. FDX1 expression is closely related to overall survival and progression-free survival in patients with low-grade glioma. FDX1 may play a role by regulating redox homeostasis, nutritional stress and the assembly process of oxygensulfur cluster complexes. In addition, it can also upregulate the expression of myeloid-derived suppressor cells, CD4^+^ T central memory T cells, macrophages, and active dendritic cells, which prevent tumor cells from escaping the surveillance of the immune system (Lu et al., 2022; Ye et al., 2022). FDX1 has also been mentioned as a prognostic marker and potential therapeutic target in hepatocellular carcinoma (Zhao et al., 2022). In addition, in adrenocortical carcinoma, kidney renal clear cell carcinoma, head and neck squamous cell carcinoma, mesothelioma, and thyroid carcinoma, FDX1 also serves as an independent prognostic factor and a potential prognostic marker (Zhang et al., 2022a). However, there is no evidence indicating the expression of FDX1 in SCI, which can be used as an important direction for future research on cuproptosis in SCI.

The LIAS participates in energy metabolism and the antioxidant response. It can synthesize α-lipoic acid, which is an essential cofactor for the pyruvate dehydrogenase complex and α-ketoglutarate dehydrogenase complex. Both the pyruvate dehydrogenase complex and the α-ketoglutarate dehydrogenase complex play important roles in energy formation (Yi et al., 2009). In cuproptosis, LIAS is regulated by FDX1 and then links the lipoyl moiety to DLAT. This further leads to DLAT oligomerization and proteotoxic stress (Tsvetkov et al., 2022). Currently, LIAS is considered a novel biomarker for prognostic prediction and the immune response in several cancer diseases. In cholangiocarcinoma, hepatocellular carcinoma, lung adenocarcinoma and lung squamous cell carcinoma, the expression of LIAS is upregulated, whereas it is downregulated in invasive breast carcinoma, colon adenocarcinoma, kidney renal clear cell carcinoma, kidney renal papillary cell carcinoma, prostate adenocarcinoma, rectum adenocarcinoma, thyroid carcinoma, and uterine corpus endometrial carcinoma. In kidney renal clear cell carcinoma and breast cancer, high expression of LIAS is associated with good survival. In contrast, higher expression of LIAS is associated with worse survival in lung cancer patients (Cai et al., 2022).

GLS plays a vital role in energy metabolism and promotes cell growth. It can convert glutamine to glutamate, which can be used to produce adenosine triphosphate (ATP) via the TCA cycle or for the synthesis of other amino acids and lipids (Song et al., 2018a). According to Liu’s report (Liu et al., 2022c), GLS is negatively correlated with cuproptosis. GLS is regarded as a protective factor against acute myocardial infarction, and it can effectively distinguish acute myocardial infarction from stable coronary heart disease, which makes it a promising therapeutic target for acute myocardial infarction. In breast cancer, GLS can be used as a diagnostic biomarker, and it can also be used for a more efficient classification of molecular subtypes in breast cancer (Zhang et al., 2023a). High GLS expression is also associated with decreased progression-free survival in prostate cancer patients treated with radiotherapy (Mukha et al., 2021).

LIPT1 is a gene that can encode components of the lipoic acid pathway, which plays a vital role in the process of cuproptosis. It can transfer lipoate to the E2 subunits of 2-ketoacid dehydrogenase complexes and regulate the oxidative and reductive metabolism of glutamine. Deficiency of LIPT1 can diminish lipoylation of the E2 subunits, which affects the biosynthesis and function of lipoic acid and fatty acylation (Liu et al., 2022b). Defects in LIPT1 may also lead to lactic acidosis, shock, and psychomotor regression (Stowe et al., 2018). LIPT1 is reportedly upregulated in osteoarthritis and can also be used to predict the possibility of the occurrence of osteoarthritis (Wang et al., 2023b). LIPT1 also serves as a potential prognostic biomarker for uterine corpus endometrial carcinoma, as high expression of LIPT1 leads to poor prognosis (Chen, 2022). In addition, in breast cancer, kidney renal clear cell carcinoma, ovarian cancer, and gastric cancer, high expression of LIPT1 is also associated with a favorable prognosis (Liu et al., 2022b).

DLD is one of the catalytic components of the pyruvate dehydrogenase complex, and it is also a key enzyme of α-ketoglutarate dehydrogenase, branched-chain α-keto acid dehydrogenase, α-ketoadipate dehydrogenase, and glycine decarboxylase complexes (Duarte et al., 2021). DLD is involved in the TCA cycle by regulating the decarboxylation of pyruvate, which indicates its vital role in mitochondrial metabolism (Yang et al., 2023). DLD acts as a positive regulator of cuproptosis, enhancing copper-related cell death. In addition to ASCI, high expression of DLD is related to the risk of primary Sjögren’s syndrome and rheumatoid arthritis (Zhang et al., 2022b; Jiang et al., 2023).

DLAT participates in the synthesis of the pyruvate dehydrogenase complex, which plays an important role in glucose metabolism and the TCA cycle (Tsvetkov et al., 2022). It plays a role in the conversion of pyruvate to acetyl-coenzyme A. The overexpression of DLAT could inhibit the production of acetyl-coenzyme A. In cuproptosis, copper can induce the oligomerization of DLAT, which causes an increase in insoluble DLAT and ultimately leads to cell death (Chen et al., 2022). The DLAT is regarded as a biomarker in hepatocellular carcinoma since it is closely related to the prognosis of hepatocellular carcinoma patients. The upregulation of DLAT is associated with poor outcomes for patients (Bai et al., 2022).

SLC31A1, which is also a cuproptosis-related gene, plays an important role in regulating intracellular copper homeostasis. It can encode a copper transporter that can affect the uptake of copper (Zimnicka et al., 2011; Maryon et al., 2013b). SLC31A1 is considered the most promising cuproptosis-related gene in breast cancer and serves as a biomarker that indicates poor patient prognosis (Li et al., 2022b). SLC31A1 also acts as a biomarker for prognostic prediction in adrenocortical carcinoma, bladder urothelial carcinoma, breast invasive carcinoma, kidney renal clear cell carcinoma, brain lower grade glioma, mesothelioma, and skin cutaneous melanoma. In addition, it can also be used to guide the pathological staging of adrenocortical carcinoma, kidney renal clear cell carcinoma, ovarian serous cystadenocarcinoma, and thyroid carcinoma (Zhang et al., 2023d).

## Copper Metabolism

Copper is an essential trace element in living organisms that functions as an essential cofactor to ensure the normalization of biochemical processes in the body. In biological systems, copper has two oxidation states, cuprous (Cu^+^) and cupric (Cu^2+^), and is found mainly in the Cu^2+^ form (Chen et al., 2023). In the presence of oxygen or other electron acceptors, Cu^+^ is readily oxidized to Cu^2+^. This redox activity of Cu^2+^ has been harnessed for catalysis by a plethora of enzymes whose activities are critical to a broad range of cellular biochemical and regulatory functions in organisms (Kim et al., 2008).

**Figure 8:**
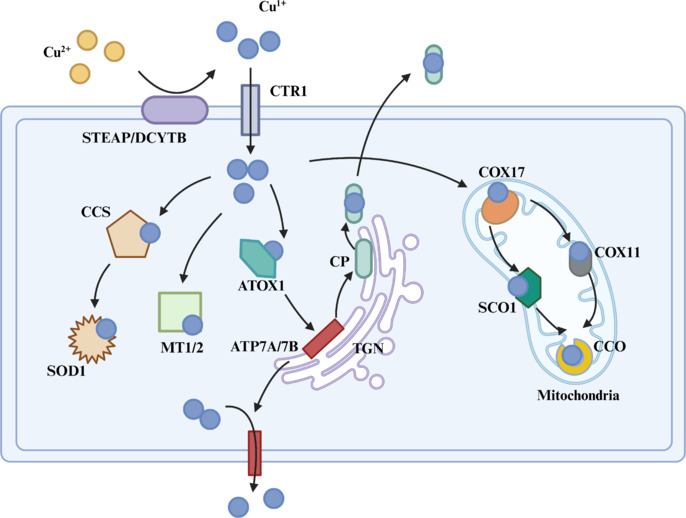
Copper metabolism after SCI at the molecular level. Extracellular Cu^2+^ is reduced by the reductase STEAP or DCYTB to Cu^+^ and then transported into the cell by CTR1. After entering the cell, copper can be stored in MT1/2 or be shuttled to specific proteins by binding to specialized chaperones. The chaperone CCS delivers copper to SOD1 to detoxify ROS. The chaperone COX17 transports copper to either SCO1 or COX11 and delivers it to CCO to activate the activity of enzymes in the respiratory chain. ATOX1 is responsible for transferring copper to ATP7A and ATP7B in the TGN, which facilitates the synthesis of CP. When the amount of intracellular copper increases, ATP7A/7B translocates from the TGN to vesicular compartments and fuses with the plasma membrane to export copper. Created with BioRender.com. ATOX1: Antioxidant protein 1; ATP7A: ATPase copper transporting alpha; ATP7B: ATPase copper transporting beta; CCO: cytochrome c oxidase; CCS: copper chaperone for superoxide dismutase; COX11: cytochrome c oxidase copper chaperone 11; COX17: cytochrome C oxidase copper chaperone 17; CP: ceruloplasmin; CTR1: copper transporter 1; DCYTB: duodenal cytochrome b; MT: metallothionein; ROS: reactive oxygen species; SCI: spinal cord injury; SCO1: cytochrome c oxidase; SOD1: superoxide dismutase 1; STEAP: six-transmembrane epithelial antigen of prostate; TGN: trans-Golgi network.

The body absorbs copper primarily from legumes, mushrooms, chocolate, nuts and seeds, and the liver (all > 2.4 μg/g) (Collins and Klevay, 2011). Absorption occurs mainly in the upper small intestine (Collins and Klevay, 2011), where copper enters intestinal epithelial cells, and this process is mediated mainly by copper transport protein (CTR1), which is located on the apical membrane and in intracellular vesicular compartments (Nose et al., 2006). In addition, intestinal cells can also take up copper partly via DMT1 (a new metal-ion transporter in the rat) (Ilyechova et al., 2019). Since only Cu^+^ can be transported, Cu^2+^ is reduced to Cu^+^ in the presence of prostate six-transmembrane epithelial antigen of the prostate and duodenal cytochrome B (Knutson, 2007). Following absorption by the gastrointestinal tract, copper is secreted into the bloodstream via ATP7A and bound to soluble chaperones (Wang et al., 2012), with approximately 40%–70% of copper ions bound to CP in the nonexchangeable form, approximately 10%–15% of copper ions bound to human serum albumin, and approximately 5%–15% bound to transcuprein/macroglobulin (Linder, 2016). In this process, the inherited defect in ATP7A leads to impaired copper ion transport within the intestinal epithelium, which in turn causes copper deficiency (Myint et al., 2018). Upon reaching the liver, hepatocytes mediate the uptake of copper via CTR1. Copper in the liver can be delivered by Cu chaperones to specific proteins or chelated by MT and GSH, thus preventing it from causing cell damage (Xue et al., 2023a). Among the Cu chaperones, by binding to cytochrome C oxidase copper chaperone 17 (COX17), it can be delivered to SCO1 and/or SCO2 (mitochondrial copper-binding proteins) for subsequent incorporation into cytochrome c oxidase to affect mitochondrial metabolism. By binding to copper chaperones for CCS, it can be delivered to SOD1, which acts as an antioxidant. The binding of copper to antioxidant protein 1 can shuttle copper to Cu-transporting ATPases, copper-transporting ATP7A and transmembrane copper-transporting ATPase 2 (ATP7B) in the TGN (Członkowska et al., 2018). In addition to excreting excess copper into the bile, ATP7B also activates the synthesis of functional CP by packaging six Cu molecules into apoceruloplasmin, which is then secreted into the plasma (Członkowska et al., 2018). In the presence of increased intracellular copper concentrations, ATP7A and ATP7B can be translocated from the TGN to vesicular compartments and fuse with the plasma membrane to export copper. For example, in the liver, ATP7B mobilizes Cu in secretory vesicles to the bile to maintain copper homeostasis in cells (Chen et al., 2020b; **Figure 8**).

## Potential Sources of Copper

The causes of elevated copper concentrations after SCI are not fully understood. Recent studies have indicated that the elevation of copper after SCI may be associated with the inability of copper ions to be effectively consumed. Previously, researchers investigated the serum concentrations of copper in patients suffering from traumatic SCI and found that patients showed a decreasing trend of copper which recovered effectively after SCI within the first 24 hours whereas the levels of copper increased in the non-remission group, leading researchers to speculate that effective copper depletion may be beneficial for the recovery from nerve injury (Seelig et al., 2020). The assumption of existence of ongoing beneficial copper consuming processes is also supported by Klevay (2013). In a previous study, we also observed an elevation of serum copper ions in a model of myocardial ischemia-reperfusion. In this study, the elevated level of serum copper was caused by the loss of copper content in the heart (He and James Kang, 2013). This provides stronger support that the elevated level of copper in the serum is accompanied by intracellular copper deficiency post-SCI. Based on the myocardial ischemia-reperfusion model, the mechanism underlying the elevation of copper may be produced by ischemia, one of the destructive events associated with SCI. Therefore, we hypothesized that the elevation of copper after SCI is also related to local ischemia. Based on previous studies, we proposed the following conjecture.

Neurons are a likely source of the increased levels of copper; this is because copper can be released from neurons in response to stimulation of the glutamatergic NMDAR (Schlief et al., 2005; Schlief and Gitlin, 2006) which become hyperexcitable after SCI (Gu et al., 2023). Research has demonstrated that the activation of NMDA results in the rapid and reversible trafficking of ATP7A to the somato-dendritic and axonal compartments of cultured hippocampal neurons. This may be related to the calcium-dependent biochemical cascade because it was inhibited by the chelation of intracellular Ca^2+^ while the blockade of voltage-gated calcium channels and voltage-gated sodium channels has no effect (Schlief and Gitlin, 2006). Later, Dodani et al. (2011b) reported that neuronal cells transport significant pools of copper from their somatic cell bodies to extended outer processes when activated by depolarization, thus reinforcing the theory described above. This conjecture has also been mentioned in other studies. Liu et al. (2021) developed a novel and surface-enhanced Raman scattering probe (CuSP), for real-time tracking and the accurate quantification of extracellular concentrations of Cu^+^ and Cu^2+^ simultaneously in the cerebral cortex of live animals. With the use of MK-801, a widely used non-competitive antagonist of NMDARs, these authors found that the extracellular concentrations of copper in the cortex during ischemia were reduced when compared to those of untreated mice. Collectively, these observations suggest that the activation of NMDARs ascribed to the depolarization of neurons resulted in the release of copper from neurons. Furthermore, researchers also reported that the change in pH and the burst of ROS after ischemia can destroy CP structures to release copper ions *in vitro*. Furthermore, the generation of ROS can induce the conversion of Cu^+^ to Cu^2+^ which may lead to cytotoxicity, thus triggering cell death. In addition, copper homeostasis in the spinal cord is also regulated by a biological barrier; the destruction of the barrier between the serum and spinal cord may also lead to the transfer of copper from serum to the spinal cord. Astrocytes have been referred to as a “copper sponge” and are responsible for the large amount of copper in the spinal cord. The dysfunctionality of astrocytes may also represent a route for the elevation of copper after SCI. One possibility is that copper ions are released directly from injured astrocytes. Copper cannot be effectively transported to other cells, thus leading to the accumulation of copper. In addition, it has been reported that inflammation can increase the concentration of copper; this is because the production of copper in hepatocytes is stimulated by pro-inflammatory ILs (Wang et al., 2023a).

**Figure 9:**
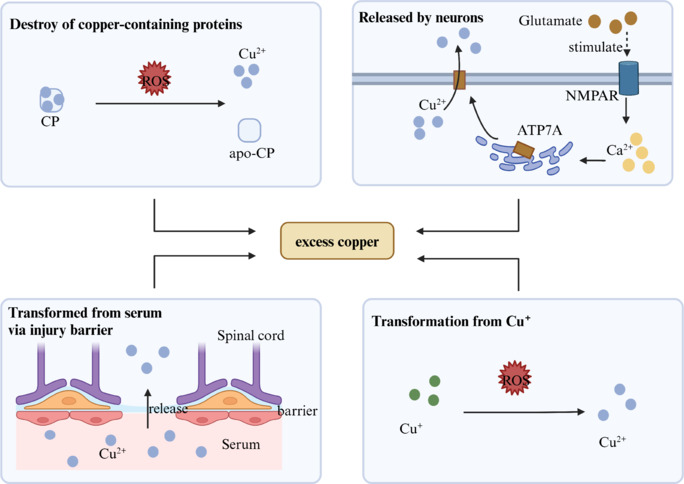
The potential source of elevated copper after SCI. After SCI, the elevated copper concentration could be due to the following routes: 1) transportation from neurons because of the activation of NMDARs; 2) release from the destroyed CP because of the decrease in ROS generation and pH upon ischemia; 3) conversion of Cu^+^ to Cu^2+^ accompanied by the generation of ROS; and 4) destruction of the barrier between the serum and the spinal cord. Created with BioRender.com. ATP7A: ATPase copper transporting alpha; CP: ceruloplasmin; NMDAR: N-methyl-D-aspartic acid receptor; PH: acid‒base balance; ROS: reactive oxygen species; SCI: spinal cord injury.

Therefore, we hypothesize that, several routes are responsible for the elevation of copper following SCI: transportation from neurons due to the activation of NMDARs, the release of copper from CP destruction due to ROS generation and pH reduction upon ischemia, the conversion of Cu^+^ to Cu^2+^ accompanied by the generation of ROS, the destruction of the barrier between the serum and spinal cord, the dysfunction of astrocytes, and inflammation. Impaired cellular function caused by intracellular copper deficiency and cytotoxicity caused by the elevation of copper in the serum ultimately promotes inflammation, oxidative stress and cell death (**Figure 9**).

## Potential Therapeutic Strategies

At present, the clinical management of copper dysregulation disorders, including various cancers and neurodegenerative diseases (including Wilson’s, Alzheimer’s and Parkinson’s diseases), provides a foundation based on safe, effective and rational approaches. In these diseases, copper chelators and copper ionophores have been identified as potential agents for the regulation of copper homeostasis. While chelating agents bind copper directly in blood and tissues and facilitate its excretion, copper ionophores can deliver copper into cells. Zinc salts have also been used to reduce the intestinal uptake of copper and act by inducing MT. Given that the serum levels of copper are elevated following SCI, strategies aimed at reducing the serum levels of copper, such as the administration of copper chelators, may prove to be effective therapeutic interventions. Conversely, previous studies have indicated that copper supplementation can mitigate neurological damage after SCI when administered prophylactically 7 days prior to injury or fifteen minutes after injury (Tural et al., 2021; Garcia et al., 2022). This suggests that prophylactic copper supplementation before SCI or early after SCI may contribute to a reduction in the severity of injury. Consequently, further investigation is now warranted to determine the optimal timing for copper supplementation versus depletion following SCI, as well as to establish effective control over the concentrations of copper. In this context, we will next discuss several pharmacological agents that can modulate copper homeostasis.

### Copper chelators

Copper chelators include D-penicillamine (D-pen), trientine and tetrathiomolybdate (TM). These agents can reduce copper concentration by chelating copper ions. However, it should be noted that brain damage caused by copper toxicity is irreversible with copper chelators (Weiss et al., 2013). Therefore, the timeliness of using copper chelators is of significant importance.

D-pen is the U.S. Food and Drug Administration-approved medication currently employed for the long-term management of rheumatoid arthritis and Wilson’s disease (Kumar et al., 2021; Tang et al., 2022b). D-pen is notable for being the first oral drug introduced for the treatment of Wilson’s disease, with various studies substantiating its efficacy (Członkowska and Litwin, 2017; Socha et al., 2022). It has also has been claimed that D-pen may exert positive effects on some patients with Alzheimer’s disease (Squitti et al., 2017). D-pen has been demonstrated to exert significant antitumor effects in preclinical trials, although clinical trials have not yet yielded exciting results. D-pen has been used in Phase II trials for the treatment of glioblastoma by anti-angiogenesis. As a copper chelator, D-pen promotes the excretion of copper from urine by chelating copper, thus reducing the serum levels of copper ions after SCI, thereby reducing oxidative damage (Squitti et al., 2017). In addition, D-pen also acts as an immunosuppressive drug to inhibit macrophage activity, reduce IL-1, and suppress T-cell activity for the treatment of rheumatoid arthritis (Kumar et al., 2021). Therefore, D-pen may alleviate the excessive inflammatory response following SCI. However, the excessive excretion of copper can lead to the frantic mobilization and redistribution of copper in tissues, thus exacerbating neurological symptoms (Squitti et al., 2017). Furthermore, the deficiency of copper caused by D-pen can lead to angiogenesis disorders, a strategy that is primarily utilized for the treatment of cancer (Brem et al., 2005). As adequate blood supply is one of the conditions for the recovery of neurological function, the use of D-pen may inhibit the recovery of neurological function after SCI (Mirastschijski et al., 2013; Ge et al., 2022a). Furthermore, copper also plays an essential role in the formation of myelin (Klevay, 2013). The overuse of copper chelators may influence the functional recovery of SCI. It has been reported that the overzealous treatment of Wilson’s disease with D-pen can cause demyelination of the CNS (Narayan and Kaveer, 2006). Furthermore, the use of D-pen can lead to adverse effects, including bone marrow toxicity, elastosis cutis, nephrotoxicity or lupus-like syndrome (Weiss et al., 2013). Therefore, the efficacy of D-pen for SCI, as well as the timing and concentration of its administration, still requires further research.

Trientine is similar to D-pen and can also form stable complexes with copper, reducing the concentration of copper in the body by urinary excretion. Although trientine is better tolerated than D-pen, the rate of neurological deterioration with trientine is slightly higher than D-pen; this may be related to the rapid mobilization of copper in the brain following the administration of chelators (Weiss et al., 2013; Kamlin et al., 2024).

TM is an alternative to D-pen and is regarded to have a better safety profile (Weiss et al., 2013). TM may represent a promising alternative as it is fast-acting and can restore a normal copper balance within several weeks compared to the several months required for other copper chelators or zinc (Brewer et al., 2009). Compared to the frequent and serious adverse events associated with D-pen and trientine treatment (Kumar et al., 2021), TM has a significantly lower rate of neurological worsening, potentially due to its ability to mobilize copper stores and further elevate copper in the brain (Brewer et al., 1987; Bandmann et al., 2015). However, TM also has demonstrated antiangiogenic characteristics and the ability to reduce proangiogenic cytokines, including vascular endothelial growth factor, IL-6, IL-8, and basic fibroblast growth factor which is widely used in cancer treatment (Pan et al., 2002). TM has been used in Phase II clinical studies for patients with high risk of recurrence for breast cancer and in preclinical models of lung metastasis as it can cause copper depletion to reduce cell proliferation, blood vessel formation, tumor growth, and motility (NCT00195091) (Chan et al., 2020). TM was also used in the Phase II trial for esophageal cancer (NCT00176800) (Schneider et al., 2013). Whether copper chelators are beneficial for the treatment of SCI has yet to be elucidated.

### Cu ionophores

Cu ionophores include ES and DSF. In the hypothesis described above, the ineffective utilization of copper is one of the reasons underlying neurological dysfunction. Therefore, copper ionophores, which can transform copper into cells may provide a potential treatment option for SCI.

ES, a small, highly lipophilic Cu^2+^-binding molecule, restores mitochondrial function by delivering the copper to cuproenzymes, such as cytochrome c oxidase in brain mitochondria. This mechanism could alleviate the neurodegenerative aspects of MD (Guthrie et al., 2020). Thus, ES holds promise for the treatment of SCI by transforming copper into cells that are in a state of copper deficiency. Based on previous research, it is indicated that copper is essential for the functionality of ES while ES alone has little therapeutic effect. The elevated levels of copper in the serum after SCI led us to hypothesize that the treatment of SCI may be conducted independently utilizing ES (Guthrie et al., 2020). Although its potential therapeutic benefits are noteworthy, the associated risks cannot be overlooked. For example, previous research has demonstrated that ES can inhibit cell growth, induce apoptosis, and damage DNA. These effects may be associated with the duration and concentration of ES treatment. As ES can transform copper into mitochondria, the excessive copper may lead to the induction of oxidative stress. It is also shown that the ES that is released from the cell following the transport of copper into the cell is capable of facilitating the further influx of copper into the cell. Moreover, Cu^2+^ transformed into cells by ES may be reduced to more toxic Cu^+^ (Hasinoff et al., 2015). These findings clearly depict the adverse events that can be caused by ES. Therefore, whether ES can be used to treat SCI still needs further investigation. Currently, ES has completed clinical Phase I, Phase II (NCT00084214) (O’Day et al., 2009), and Phase III (NCT00522834) (O’Day et al., 2013) for melanoma, Phase II clinical trials for recurrent or persistent platinum-resistant ovarian, fallopian tube or primary peritoneal cancer, and primary and recurrent primary peritoneal cancer (NCT00888615) (Monk et al., 2018), as well as Phase II clinical trials for soft tissue sarcoma (NCT00087997). Collectively, these studies indicate that ES is safe but does not provide significant efficacy.

DSF, a drug used for the treatment of alcohol dependence and approved by the U.S. Food and Drug Administration, has been scientifically validated for its safety and potential for the targeted therapy of tumors (Zhou et al., 2023). In recent research, DSF was shown to act as a potential anti-inflammatory agent by inhibiting multiple inflammatory reactions and regulating inflammation-related targets. In the context of cerebral ischemia, researchers found that the total amount of copper ions decreased following DSF administration, while copper ions were increased in the transient middle cerebral artery occlusion model (Yang et al., 2024). This preserves mitochondrial structural integrity as copper can lead to mitochondrial morphological damage and dysfunction, thus attenuating inflammatory responses and promoting neurological recovery. DSF plays its role mainly by inhibiting the expression of FDX that can help convert Cu^2+^ to the more toxic Cu^+^ (Yang et al., 2024). This provides a possible therapeutic target for some diseases associated with ischemia-reperfusion injury which are also involved in SCI, thus suggesting the potential therapeutic value of DSF in SCI. However, it has also been reported that DSF and its major metabolite (diethyldithiocarbamate) can bind to copper to trigger the production of ROS which may aggravate oxidative stress post-SCI. DSF can also induce apoptosis, and reduce angiogenesis. These all represent negative effects on the recovery of SCI (Li et al., 2020a). Based on these effects, DSF is widely used in the prevention and treatment of various types of cancers (Li et al., 2024b). Currently, DSF has completed Phase I clinical trials for solid tumors (NCT00742911) (Kelley et al., 2021), early Phase I (NCT01907165) (Huang et al., 2016), Phase I and Phase II (NCT02770378) (Halatsch et al., 2021) clinical trials for glioblastoma, Phase II clinical trials for recurrent glioblastoma (NCT03034135) (Huang et al., 2019a), Phase II and Phase III clinical trials for non-small cell lung cancer (NCT00312819) (Nechushtan et al., 2015), and Phase I clinical trials (NCT02963051) for prostate cancer (Zhang et al., 2022c).

### Copper nano-complexes

According to the results of a previous study, prophylactic copper supplementation or early copper supplementation may serve as a viable therapeutic intervention; adult female Sprague-Dawley rats received SCI followed by daily doses of copper (6.5 mg/kg) (Garcia et al., 2022). This concentration is adequate for diets, and can also prevent rats from diabetes or impaired glucose tolerance (Rhee et al., 2004). However, there is no evidence relating to the appropriate concentration of copper that should be used in clinical research; this requires further investigation.

Although it is possible to modulate copper homeostasis by copper supplementation, it appears that positive effects can be gained by taking oral medicine. However, this practice is limited by the inadequate absorption of oral medicines and the inherent constraints of the blood–brain barrier (BBB). In addition, because of their metallic toxicity, the widespread usage of medicines is limited. The development of copper nano-complexes provides an effective solution to this problem. One of the advantages of copper nano-complexes is that they are not transported in an ionic state, thus mitigating the toxic impairment caused by copper ions to normal cells. These copper nano-complexes can also effectively across the BBB to improve the efficiency of delivery (Zhu et al., 2024c). Nowadays, a variety of copper nano-complexes have been developed to be used for the treatment of cancer. In glioma cells, copper oxide nanoparticles can induce glioma cytotoxicity injury (Joshi et al., 2016) by accumulating copper in cells. Nitrobenzene-cysteine-Cu^2+^ nano-complexes have been developed to reduce cancerous and precancerous cells by anti-angiogenesis (Kang et al., 2023). Due to the presence of the BBB, the efficiency of transportation remains a significant problem. The combination of thermosensitive liposomal and Cu-Fe bimetal was developed to treat breast cancer in mice by inducing cuproptosis in tumor cells (Chen et al., 2024). In this study, researchers used live neutrophils to design MetaCell, a cellular Trojan horse that can internalize thermosensitive liposomal bimetallic Fe-Cu MOFs. Compared to other cells, neutrophils can skillfully evade clearance mechanisms and permeate biological barriers; consequently, neutrophils could represent a potential tool to effectively deliver copper through the BBB. Thus, there are several options to improve copper utilization and transportation efficiency in neural cells post-SCI. Furthermore, some nanomaterials can detect micromolar changes in copper concentration (Patel et al., 2011), and could be used to detect micro-changes of copper in different cells post-SCI.

### Copper-based scaffolds/biomaterial strategies

Implantable scaffolds have been developed to be applied in SCI. These can promote cell proliferation, cell differentiation, cell attachment, and the reconstruction of nerve circuits and blood vessels. These scaffolds exert minimal toxicity to cells and induce minimal inflammation. In addition to providing mechanical support at injury sites, they can also deliver molecules, gases, and liquids (Shen et al., 2022). Hydrogels are one of the most widely used biomaterials for scaffolds. The mechanical properties of hydrogels is similar to those of the spinal cord and can promote the formation of a normal anatomical structure. In addition, hydrogels possess a three-dimensional network structure formed by various crosslinking reactions, thus creating a suitable candidate for cell growth and consistent drug delivery. Copper-based hydrogels are widely used for tissue regeneration because they play an essential role in the proliferation, adhesion, and differentiation of stem cells (Shen et al., 2022; Xue et al., 2022). Furthermore, the copper used in hydrogels can form coordination bonds, thus improving mechanical strength and tensile properties (Xue et al., 2022). Researchers have produced a hyaluronan-copper hydrogel that can accelerate infectious wound healing; these effects are related to the excellent antibacterial, pro-healing, and proangiogenic properties of copper (Qian et al., 2022). In addition to its role in bone repair, copper also can be used for chondrogenic differentiation (Xue et al., 2022). As copper can produce H_2_O_2_ via the Fenton reaction, a Cu^2+^-loaded ionic gel (D-IL-Gel) has been designed which exhibits efficient antibacterial and wound healing performance (Jia and Wu, 2023). CuS nanoparticles can also be introduced into the hydrogel network structure to produce hybrid hydrogels, which exhibit outstanding antibacterial properties (Yang et al., 2025). However, copper-containing hydrogels can also induce mitochondrial dysfunction, apoptosis, and lipid peroxidation. Consequently, these hydrogels can be used for cancer treatment as they can induce the generation of excess ROS (Cabral et al., 2023). Therefore, it is important to design hydrogels that can control the amount of copper released into the injury site. Because of the different pathological processes in different SCI phases, regulating the timing of copper release is very important; this is a crucial aspect of future research.

### Other potential treatments

N-methyl-D-aspartate (NMDA) antagonists, such as memantine, which has been approved by the U.S. Food and Drug Administration for Alzheimer’s disease treatment (Li et al., 2020b), can also be used to relieve neuropathic pain after SCI (Rafe et al., 2024). In addition, by binding to copper, the cellular prion protein can induce the inhibition of NMDARs, minimizing excitotoxicity susceptibility and promoting neuroprotection (Li et al., 2020b). On the basis of the potential links between copper and calcium homeostasis mentioned above, chelators of intracellular Ca^2+^, such as BAPTA, are also possible treatments for decreasing the release of copper. In the past, BAPTA (1,2‐bis(o‐aminophenoxy)ethane‐N,N,N’,N’‐tetraacetic acid) has long been recognized as a neuroprotective agent because of its ability to inhibit neuronal apoptosis and ROS generation (Kang et al., 2021).

As mentioned above, ROS have a significant effect on elevated copper levels by destroying the structure of CP to release copper ions *in vitro* and inducing the conversion of Cu^+^ to Cu^2+^. This finding also explains why the use of ROS inhibitors may promote motor recovery from SCI.

Therefore, the potential treatments can be summarized as follows: 1) use copper chelators to reduce the concentration of copper in serum; 2) use copper ionophores to deliver copper into cells, which may restore the function of copper-containing proteins; 3) inhibit multiple inflammatory reactions; 4) inhibit the conversion of Cu^2+^ to the more toxic Cu^+^; 5) use an NMDA antagonist to decrease the release of copper; 6) use ROS inhibitors to protect copper-containing proteins from destruction; and 7) use appropriate copper supplementation to counteract cellular copper deficiency.

## Conclusion

SCI is a neurological disease that not only causes significant physical and psychological trauma to patients but also imposes an additional financial burden. Enhancing neuroplasticity and promoting axonal regeneration following SCI are novel strategies. One possible therapeutic strategy that could avoid further patient deterioration is supplementation with vitamin E or trace elements, such as zinc, selenium, and copper. Of these, copper is an essential trace element that plays a fundamental role in the biochemistry of the CNS, including neuronal firing, synaptic plasticity and myelin formation. Copper also acts as a coenzyme for many proteins and plays an important role in mitochondrial respiration, antioxidant defense and biosynthesis. Many neurological disorders are accompanied by copper deficiency. Very few studies have investigated the role of copper in SCI, although a recent study demonstrated that the serum levels of copper were elevated post-SCI. This highlighted a potential link between copper and SCI, although many questions remain unanswered. For example, we need to understand exactly why the concentration of copper ions increase in the serum post-SCI. We also need to investigate how the elevation of copper ions after SCI may subsequently influence the pathological process of SCI. By answering these questions, we may be able to identify new treatment options.

However, due to the restricted sample size and the fact that most existing research studies are confined to animal studies, the precise mechanisms by which copper influences SCI, the underlying causes for the elevated copper levels following SCI, and the impact of copper on SCI in humans remain unclear. We can only speculate on possible links from similar models, and some biochemical processes that already have been demonstrated. The elevation of copper ions also occurrs in some models of ischemia, such as a model of myocardial ischemia-reperfusion and a model of cerebral ischemia, thus providing important reference information for future research. These models have confirmed that the elevation of serum copper ions after local ischemia is accompanied by a deficiency in the intracellular concentration of copper ions. From this, we deduced that the elevation of copper ions post-SCI might be related to the ineffective utilization of copper ions in cells. This could not only impair normal cellular function but also lead to the potential cytotoxicity of copper ions released into the serum. Such an increase in serum copper levels may further exacerbate SCI through three major pathways: inflammation, oxidative stress, and cell death. In a model of cerebral ischemia, researchers using a novel SERS probe (CuSP) discovered three mechanisms contributing to increased copper concentrations: (1) copper transport from neurons due to NMDARs activation; (2) release of copper from CP disrupted by ROS generation and decreased pH during ischemia; and (3) the oxidation of Cu^+^ to Cu^2+^, accompanied by ROS production. As ischemic processes are also a feature of SCI, it is plausible that these mechanisms contribute to elevated copper ions after SCI. However, whether these mechanisms apply to SCI remains to be fully elucidated and requires further investigation. Based on the sources and consequences of copper dysregulation, we propose several therapeutic strategies: copper chelators to reduce the concentration of copper in serum; copper ionophores to deliver copper into cells to restore the functionality of copper-containing proteins, the inhibition of multiple inflammatory reactions, and inhibition of the conversion of Cu^2+^ to the more toxic Cu^+^; NMDA antagonists to reduce the release of copper; ROS inhibitors to protect the copper-containing proteins from destruction; and appropriate copper supplementation to counteract cellular copper deficiency.

## Data Availability

*Not applicable*.

## References

[R1] Abbaszadeh F, Fakhri S, Khan H (2020). Targeting apoptosis and autophagy following spinal cord injury: therapeutic approaches to polyphenols and candidate phytochemicals. Pharmacol Res.

[R2] Ahuja CS, Nori S, Tetreault L, Wilson J, Kwon B, Harrop J, Choi D, Fehlings MG (2017). Traumatic spinal cord injury-repair and regeneration. Neurosurgery.

[R3] Al Mamun A, Wu Y, Monalisa I, Jia C, Zhou K, Munir F, Xiao J (2021). Role of pyroptosis in spinal cord injury and its therapeutic implications. J Adv Res.

[R4] Alizadeh A, Dyck SM, Karimi-Abdolrezaee S (2019). Traumatic spinal cord injury: an overview of pathophysiology, models and acute injury mechanisms. Front Neurol.

[R5] An Y, Li S, Huang X, Chen X, Shan H, Zhang M (2022). The role of copper homeostasis in brain disease. Int J Mol Sci.

[R6] Anjum A, Yazid MD, Fauzi Daud M, Idris J, Ng AMH, Selvi Naicker A, Ismail OHR, Athi Kumar RK, Lokanathan Y (2020). Spinal cord injury: pathophysiology, multimolecular interactions, and underlying recovery mechanisms. Int J Mol Sci.

[R7] Arredondo M, Muñoz P, Mura CV, Nùñez MT (2003). DMT1, a physiologically relevant apical Cu1+ transporter of intestinal cells. Am J Physiol Cell Physiol.

[R8] Aschner M (1996). The functional significance of brain metallothioneins. FASEB J.

[R9] Ashraf A, Michaelides C, Walker TA, Ekonomou A, Suessmilch M, Sriskanthanathan A, Abraha S, Parkes A, Parkes HG, Geraki K, So PW (2019). Regional distributions of iron, copper and zinc and their relationships with glia in a normal aging mouse model. Front Aging Neurosci.

[R10] Bai WD, Liu JY, Li M, Yang X, Wang YL, Wang GJ, Li SC (2022). A novel cuproptosis-related signature identified DLAT as a prognostic biomarker for hepatocellular carcinoma patients. World J Oncol.

[R11] Bandmann O, Weiss KH, Kaler SG (2015). Wilson’s disease and other neurological copper disorders. Lancet Neurol.

[R12] Barański B (1986). Effect of maternal cadmium exposure on postnatal development and tissue cadmium, copper and zinc concentrations in rats. Arch Toxicol.

[R13] Bedoui S, Herold MJ, Strasser A (2020). Emerging connectivity of programmed cell death pathways and its physiological implications. Nat Rev Mol Cell Biol.

[R14] Bhattacharjee A, Ghosh S, Chatterji A, Chakraborty K (2020). Neuron-glia: understanding cellular copper homeostasis, its cross-talk and their contribution towards neurodegenerative diseases. Metallomics.

[R15] Bietar B, Lehmann C, Stadnyk AW (2021). Effects of CNS injury-induced immunosuppression on pulmonary immunity. Life (Basel).

[R16] Bietar B, Zhou J, Lehmann C (2021). Utility of intestinal intravital microscopy for the study of CNS injury-induced immunodepression syndrome (CIDS). Clin Hemorheol Microcirc.

[R17] Bock FJ, Tait SWG (2020). Mitochondria as multifaceted regulators of cell death. Nat Rev Mol Cell Biol.

[R18] Bongarzone ER, Pasquini JM, Soto EF (1995). Oxidative damage to proteins and lipids of CNS myelin produced by in vitro generated reactive oxygen species. J Neurosci Res.

[R19] Bonnemaison ML, Duffy ME, Mains RE, Vogt S, Eipper BA, Ralle M (2016). Copper, zinc and calcium: imaging and quantification in anterior pituitary secretory granules. Metallomics.

[R20] Brem S, Grossman SA, Carson KA, New P, Phuphanich S, Alavi JB, Mikkelsen T, Fisher JD, New Approaches to Brain Tumor Therapy CNS Consortium (2005). Phase 2 trial of copper depletion and penicillamine as antiangiogenesis therapy of glioblastoma. Neuro Oncol.

[R21] Bremner I (1998). Manifestations of copper excess. Am J Clin Nutr.

[R22] Brewer GJ, Terry CA, Aisen AM, Hill GM (1987). Worsening of neurologic syndrome in patients with Wilson’s disease with initial penicillamine therapy. Arch Neurol.

[R23] Brewer GJ, Askari F, Dick RB, Sitterly J, Fink JK, Carlson M, Kluin KJ, Lorincz MT (2009). Treatment of Wilson’s disease with tetrathiomolybdate: V. Control of free copper by tetrathiomolybdate and a comparison with trientine. Transl Res.

[R24] Brezova V, Valko M, Breza M, Morris H, Telser J, Dvoranova D, Kaiserova K, Varecka L, Mazur M, Leibfritz D (2003). Role of radicals and singlet oxygen in photoactivated DNA cleavage by the anticancer drug camptothecin: an electron paramagnetic resonance study. J Phys Chem B.

[R25] Britton RS (1996). Metal-induced hepatotoxicity. Semin Liver Dis.

[R26] Brooks C, Wei Q, Feng L, Dong G, Tao Y, Mei L, Xie ZJ, Dong Z (2007). Bak regulates mitochondrial morphology and pathology during apoptosis by interacting with mitofusins. Proc Natl Acad Sci U S A.

[R27] Bulcke F, Dringen R (2016). Handling of copper and copper oxide nanoparticles by astrocytes. Neurochem Res.

[R28] Burdette BE, Esparza AN, Zhu H, Wang S (2021). Gasdermin D in pyroptosis. Acta Pharm Sin B.

[R29] Cabral FV, Santana BM, Lange CN, Batista BL, Seabra AB, Ribeiro MS (2023). Pluronic F-127 hydrogels containing copper oxide nanoparticles and a nitric oxide donor to treat skin cancer. Pharmaceutics.

[R30] Cai Y, He Q, Liu W, Liang Q, Peng B, Li J, Zhang W, Kang F, Hong Q, Yan Y, Peng J, Xu Z, Bai N (2022). Comprehensive analysis of the potential cuproptosis-related biomarker LIAS that regulates prognosis and immunotherapy of pan-cancers. Front Oncol.

[R31] Chan N (2017). Influencing the tumor microenvironment: a phase II study of copper depletion using tetrathiomolybdate in patients with breast cancer at high risk for recurrence and in preclinical models of lung metastases. Clin Cancer Res.

[R32] Chan WY, Garnica AD, Rennert OM (1978). Cell culture studies of Menkes kinky hair disease. Clin Chim Acta.

[R33] Cheeseman KH, Slater TF (1993). An introduction to free radical biochemistry. Br Med Bull.

[R34] Chen GH, Lv W, Xu YH, Wei XL, Xu YC, Luo Z (2020). Functional analysis of MTF-1 and MT promoters and their transcriptional response to zinc (Zn) and copper (Cu) in yellow catfish Pelteobagrus fulvidraco. Chemosphere.

[R35] Chen J, Jiang Y, Shi H, Peng Y, Fan X, Li C (2020). The molecular mechanisms of copper metabolism and its roles in human diseases. Pflugers Arch.

[R36] Chen K, Zhou A, Zhou X, He J, Xu Y, Ning X (2024). Cellular trojan horse initiates bimetallic Fe-Cu MOF-mediated synergistic cuproptosis and ferroptosis against malignancies. Sci Adv.

[R37] Chen Q, Wang Y, Yang L, Sun L, Wen Y, Huang Y, Gao K, Yang W, Bai F, Ling L, Zhou Z, Zhang X, Xiong J, Zhai R (2022). PM2.5 promotes NSCLC carcinogenesis through translationally and transcriptionally activating DLAT-mediated glycolysis reprograming. J Exp Clin Cancer Res.

[R38] Chen X, Cai Q, Liang R, Zhang D, Liu X, Zhang M, Xiong Y, Xu M, Liu Q, Li P, Yu P, Shi A (2023). Copper homeostasis and copper-induced cell death in the pathogenesis of cardiovascular disease and therapeutic strategies. Cell Death Dis.

[R39] Chen Y (2022). Identification and validation of cuproptosis-related prognostic signature and associated regulatory axis in uterine corpus endometrial carcinoma. Front Genet.

[R40] Chen Y, Liu S, Li J, Li Z, Quan J, Liu X, Tang Y, Liu B (2020). The latest view on the mechanism of ferroptosis and its research progress in spinal cord injury. Oxid Med Cell Longev.

[R41] Choi BS, Zheng W (2009). Copper transport to the brain by the blood-brain barrier and blood-CSF barrier. Brain Res.

[R42] Collins JF, Klevay LM (2011). Copper. Adv Nutr.

[R43] Colombo E, Triolo D, Bassani C, Bedogni F, Di Dario M, Dina G, Fredrickx E, Fermo I, Martinelli V, Newcombe J, Taveggia C, Quattrini A, Comi G, Farina C (2021). Dysregulated copper transport in multiple sclerosis may cause demyelination via astrocytes. Proc Natl Acad Sci U S A.

[R44] Członkowska A, Litwin T (2017). Wilson disease - currently used anticopper therapy. Handb Clin Neurol.

[R45] Członkowska A, Litwin T, Dusek P, Ferenci P, Lutsenko S, Medici V, Rybakowski JK, Weiss KH, Schilsky ML (2018). Wilson disease. Nat Rev Dis Primers.

[R46] D’Ambrosi N, Rossi L (2015). Copper at synapse: Release, binding and modulation of neurotransmission. Neurochem Int.

[R47] de Romaña DL, Olivares M, Uauy R, Araya M (2011). Risks and benefits of copper in light of new insights of copper homeostasis. J Trace Elem Med Biol.

[R48] Deigendesch N, Zychlinsky A, Meissner F (2018). Copper regulates the canonical NLRP3 inflammasome. J Immunol.

[R49] Di YQ, Han XL, Kang XL, Wang D, Chen CH, Wang JX, Zhao XF (2021). Autophagy triggers CTSD (cathepsin D) maturation and localization inside cells to promote apoptosis. Autophagy.

[R50] Dietz V, Fouad K (2014). Restoration of sensorimotor functions after spinal cord injury. Brain.

[R51] Díez-Tercero L, Delgado LM, Bosch-Rué E, Perez RA (2021). Evaluation of the immunomodulatory effects of cobalt, copper and magnesium ions in a pro inflammatory environment. Sci Rep.

[R52] Ding Y, Zhang D, Wang S, Zhang X, Yang J (2021). Hematogenous macrophages: a new therapeutic target for spinal cord injury. Front Cell Dev Biol.

[R53] Dodani SC, Leary SC, Cobine PA, Winge DR, Chang CJ (2011). A targetable fluorescent sensor reveals that copper-deficient SCO1 and SCO2 patient cells prioritize mitochondrial copper homeostasis. J Am Chem Soc.

[R54] Dodani SC, Domaille DW, Nam CI, Miller EW, Finney LA, Vogt S, Chang CJ (2011). Calcium-dependent copper redistributions in neuronal cells revealed by a fluorescent copper sensor and X-ray fluorescence microscopy. Proc Natl Acad Sci U S A.

[R55] Dong J, Wang X, Xu C, Gao M, Wang S, Zhang J, Tong H, Wang L, Han Y, Cheng N, Han Y (2021). Inhibiting NLRP3 inflammasome activation prevents copper-induced neuropathology in a murine model of Wilson’s disease. Cell Death Dis.

[R56] Drouin R, Rodriguez H, Gao SW, Gebreyes Z, O’Connor TR, Holmquist GP, Akman SA (1996). Cupric ion/ascorbate/hydrogen peroxide-induced DNA damage: DNA-bound copper ion primarily induces base modifications. Free Radic Biol Med.

[R57] Duarte IF, Caio J, Moedas MF, Rodrigues LA, Leandro AP, Rivera IA, Silva MFB (2021). Dihydrolipoamide dehydrogenase, pyruvate oxidation, and acetylation-dependent mechanisms intersecting drug iatrogenesis. Cell Mol Life Sci.

[R58] Evavold CL, Ruan J, Tan Y, Xia S, Wu H, Kagan JC (2018). The pore-forming protein gasdermin d regulates interleukin-1 secretion from living macrophages. Immunity.

[R59] Feng L, Sun J, Xia L, Shi Q, Hou Y, Zhang L, Li M, Fan C, Sun B (2024). Ferroptosis mechanism and Alzheimer’s disease. Neural Regen Res.

[R60] Finkel T, Holbrook NJ (2000). Oxidants, oxidative stress and the biology of ageing. Nature.

[R61] Fricker M, Tolkovsky AM, Borutaite V, Coleman M, Brown GC (2018). Neuronal cell death. Physiol Rev.

[R62] Fu X, Zhang Y, Jiang W, Monnot AD, Bates CA, Zheng W (2014). Regulation of copper transport crossing brain barrier systems by Cu-ATPases: effect of manganese exposure. Toxicol Sci.

[R63] Gaetke LM, Chow CK (2003). Copper toxicity, oxidative stress, and antioxidant nutrients. Toxicology.

[R64] Gaier ED, Eipper BA, Mains RE (2013). Copper signaling in the mammalian nervous system: synaptic effects. J Neurosci Res.

[R65] Gaier ED, Eipper BA, Mains RE (2014). Pam heterozygous mice reveal essential role for Cu in amygdalar behavioral and synaptic function. Ann N Y Acad Sci.

[R66] Galluzzi L (2017). Molecular definitions of autophagy and related processes. EMBO J.

[R67] Gao M, Monian P, Pan Q, Zhang W, Xiang J, Jiang X (2016). Ferroptosis is an autophagic cell death process. Cell Res.

[R68] Gao P, Yi J, Chen W, Gu J, Miao S, Wang X, Huang Y, Jiang T, Li Q, Zhou W, Zhao S, Wu M, Yin G, Chen J (2023). Pericyte-derived exosomal miR-210 improves mitochondrial function and inhibits lipid peroxidation in vascular endothelial cells after traumatic spinal cord injury by activating JAK1/STAT3 signaling pathway. J Nanobiotechnology.

[R69] Gao W, Huang Z, Duan J, Nice EC, Lin J, Huang C (2021). Elesclomol induces copper-dependent ferroptosis in colorectal cancer cells via degradation of ATP7A. Mol Oncol.

[R70] Garcia E, Aguilar-Cevallos J, Silva-Garcia R, Ibarra A (2016). Cytokine and growth factor activation in vivo and in vitro after spinal cord injury. Mediators Inflamm.

[R71] Garcia E, Hernández-Ayvar F, Rodríguez-Barrera R, Flores-Romero A, Borlongan C, Ibarra A (2022). Supplementation with vitamin e, zinc, selenium, and copper re-establishes T-cell function and improves motor recovery in a rat model of spinal cord injury. Cell Transplant.

[R72] Gaumer S, Guénal I, Brun S, Théodore L, Mignotte B (2000). Bcl-2 and Bax mammalian regulators of apoptosis are functional in Drosophila. Cell Death Differ.

[R73] Ge EJ (2022). Connecting copper and cancer: from transition metal signalling to metalloplasia. Nat Rev Cancer.

[R74] Ge H, Xue X, Xian J, Yuan L, Wang L, Zou Y, Zhong J, Jiang Z, Shi J, Chen T, Su H, Feng H, Hu S (2022). Ferrostatin-1 alleviates white matter injury via decreasing ferroptosis following spinal cord injury. Mol Neurobiol.

[R75] Ge MH, Tian H, Mao L, Li DY, Lin JQ, Hu HS, Huang SC, Zhang CJ, Mei XF (2021). Zinc attenuates ferroptosis and promotes functional recovery in contusion spinal cord injury by activating Nrf2/GPX4 defense pathway. CNS Neurosci Ther.

[R76] Ge X (2021). Exosomal miR-155 from M1-polarized macrophages promotes EndoMT and impairs mitochondrial function via activating NF-κB signaling pathway in vascular endothelial cells after traumatic spinal cord injury. Redox Biol.

[R77] Georgiou-Siafis SK, Tsiftsoglou AS (2023). The key role of GSH in keeping the redox balance in mammalian cells: mechanisms and significance of GSH in detoxification via formation of conjugates. Antioxidants (Basel).

[R78] Glover HL, Schreiner A, Dewson G, Tait SWG (2024). Mitochondria and cell death. Nat Cell Biol.

[R79] Gombart AF, Pierre A, Maggini S (2020). A review of micronutrients and the immune system-working in harmony to reduce the risk of infection. Nutrients.

[R80] Gong Y, Fan Z, Luo G, Yang C, Huang Q, Fan K, Cheng H, Jin K, Ni Q, Yu X, Liu C (2019). The role of necroptosis in cancer biology and therapy. Mol Cancer.

[R81] Goodall ML, Fitzwalter BE, Zahedi S, Wu M, Rodriguez D, Mulcahy-Levy JM, Green DR, Morgan M, Cramer SD, Thorburn A (2016). The autophagy machinery controls cell death switching between apoptosis and necroptosis. Dev Cell.

[R82] Gromadzka G, Tarnacka B, Flaga A, Adamczyk A (2020). Copper dyshomeostasis in neurodegenerative diseases-therapeutic implications. Int J Mol Sci.

[R83] Grubman A, White AR (2014). Copper as a key regulator of cell signalling pathways. Expert Rev Mol Med.

[R84] Gu C, Kong F, Zeng J, Geng X, Sun Y, Chen X (2023). Remote ischemic preconditioning protects against spinal cord ischemia-reperfusion injury in mice by activating NMDAR/AMPK/PGC-1α/SIRT3 signaling. Cell Biosci.

[R85] Guthrie LM, Soma S, Yuan S, Silva A, Zulkifli M, Snavely TC, Greene HF, Nunez E, Lynch B, De Ville C, Shanbhag V, Lopez FR, Acharya A, Petris MJ, Kim BE, Gohil VM, Sacchettini JC (2020). Elesclomol alleviates Menkes pathology and mortality by escorting Cu to cuproenzymes in mice. Science.

[R86] Haidari M, Javadi E, Kadkhodaee M, Sanati A (2001). Enhanced susceptibility to oxidation and diminished vitamin E content of LDL from patients with stable coronary artery disease. Clin Chem.

[R87] Hajnóczky G, Csordás G, Das S, Garcia-Perez C, Saotome M, Sinha Roy S, Yi M (2006). Mitochondrial calcium signalling and cell death: approaches for assessing the role of mitochondrial Ca2+ uptake in apoptosis. Cell Calcium.

[R88] Halatsch ME (2021). A phase Ib/IIa trial of 9 repurposed drugs combined with temozolomide for the treatment of recurrent glioblastoma: CUSP9v3. Neurooncol Adv.

[R89] Halliwell B, Gutteridge JM (1985). The importance of free radicals and catalytic metal ions in human diseases. Mol Aspects Med.

[R90] Harrington JS, Ryter SW, Plataki M, Price DR, Choi AMK (2023). Mitochondria in health, disease, and aging. Physiol Rev.

[R91] Harris ED (1992). Copper as a cofactor and regulator of copper, zinc superoxide dismutase. J Nutr.

[R92] Hasinoff BB, Wu X, Yadav AA, Patel D, Zhang H, Wang DS, Chen ZS, Yalowich JC (2015). Cellular mechanisms of the cytotoxicity of the anticancer drug elesclomol and its complex with Cu(II). Biochem Pharmacol.

[R93] He W, James Kang Y (2013). Ischemia-induced copper loss and suppression of angiogenesis in the pathogenesis of myocardial infarction. Cardiovasc Toxicol.

[R94] Hellenbrand DJ, Quinn CM, Piper ZJ, Morehouse CN, Fixel JA, Hanna AS (2021). Inflammation after spinal cord injury: a review of the critical timeline of signaling cues and cellular infiltration. J Neuroinflammation.

[R95] Heller RA, Seelig J, Crowell HL, Pilz M, Haubruck P, Sun Q, Schomburg L, Daniel V, Moghaddam A, Biglari B (2021). Predicting neurological recovery after traumatic spinal cord injury by time-resolved analysis of monocyte subsets. Brain.

[R96] Herb M, Schramm M (2021). Functions of ROS in macrophages and antimicrobial immunity. Antioxidants (Basel).

[R97] Hilton JBW, Kysenius K, Liddell JR, Mercer SW, Paul B, Beckman JS, McLean CA, White AR, Donnelly PS, Bush AI, Hare DJ, Roberts BR, Crouch PJ (2024). Evidence for disrupted copper availability in human spinal cord supports Cu(II)(atsm) as a treatment option for sporadic cases of ALS. Sci Rep.

[R98] Horn N, Wittung-Stafshede P (2021). ATP7A-regulated enzyme metalation and trafficking in the Menkes disease puzzle. Biomedicines.

[R99] Hou W, Han J, Lu C, Goldstein LA, Rabinowich H (2010). Autophagic degradation of active caspase-8: a crosstalk mechanism between autophagy and apoptosis. Autophagy.

[R100] Hou W, Xie Y, Song X, Sun X, Lotze MT, Zeh HJ, Kang R, Tang D (2016). Autophagy promotes ferroptosis by degradation of ferritin. Autophagy.

[R101] Hu X, Xu Y, Xu H, Jin C, Zhang H, Su H, Li Y, Zhou K, Ni W (2021). Progress in understanding ferroptosis and its targeting for therapeutic benefits in traumatic brain and spinal cord injuries. Front Cell Dev Biol.

[R102] Hu Y, Jia K, Zhou Y, Chen L, Wang F, Yi X, Huang Y, Ge Y, Chen X, Liao D, Peng Y, Meng Y, Liu Y, Luo Q, Cheng B, Zhao Y, Lu H, Yuan W (2023). Rutin hydrate relieves neuroinflammation in zebrafish models: Involvement of NF-κB pathway as a central network. Fish Shellfish Immunol.

[R103] Huang J, Campian JL, Gujar AD, Tran DD, Lockhart AC, DeWees TA, Tsien CI, Kim AH (2016). A phase I study to repurpose disulfiram in combination with temozolomide to treat newly diagnosed glioblastoma after chemoradiotherapy. J Neurooncol.

[R104] Huang J, Chaudhary R, Cohen AL, Fink K, Goldlust S, Boockvar J, Chinnaiyan P, Wan L, Marcus S, Campian JL (2019). A multicenter phase II study of temozolomide plus disulfiram and copper for recurrent temozolomide-resistant glioblastoma. J Neurooncol.

[R105] Huang Q, Ouyang Z, Tan Y, Wu H, Liu Y (2019). Activating macrophages for enhanced osteogenic and bactericidal performance by Cu ion release from micro/nano-topographical coating on a titanium substrate. Acta Biomater.

[R106] Husain N, Mahmood R (2019). Copper(II) generates ROS and RNS, impairs antioxidant system and damages membrane and DNA in human blood cells. Environ Sci Pollut Res Int.

[R107] Ilyechova EY, Bonaldi E, Orlov IA, Skomorokhova EA, Puchkova LV, Broggini M (2019). CRISP-R/Cas9 mediated deletion of copper transport genes CTR1 and DMT1 in NSCLC cell line H1299. Biological and pharmacological consequences. Cells.

[R108] Izadi M, Ali TA, Pourkarimi E (2021). Over fifty years of life, death, and cannibalism: a historical recollection of apoptosis and autophagy. Int J Mol Sci.

[R109] Jaiser SR, Winston GP (2010). Copper deficiency myelopathy. J Neurol.

[R110] Jenner A, Peña-Blanco A, Salvador-Gallego R, Ugarte-Uribe B, Zollo C, Ganief T, Bierlmeier J, Mund M, Lee JE, Ries J, Schwarzer D, Macek B, Garcia-Saez AJ (2022). DRP1 interacts directly with BAX to induce its activation and apoptosis. EMBO J.

[R111] Jia C, Wu FG (2023). Antibacterial chemodynamic therapy: materials and strategies. BME Front.

[R112] Jiang M, Liu K, Lu S, Qiu Y, Zou X, Zhang K, Chen C, Jike Y, Xie M, Dai Y, Bo Z (2023). Verification of cuproptosis-related diagnostic model associated with immune infiltration in rheumatoid arthritis. Front Endocrinol (Lausanne).

[R113] Jiayi H, Ziyuan T, Tianhua X, Mingyu Z, Yutong M, Jingyu W, Hongli Z, Li S (2024). Copper homeostasis in chronic kidney disease and its crosstalk with ferroptosis. Pharmacol Res.

[R114] Jomova K, Hudecova L, Lauro P, Simunková M, Barbierikova Z, Malcek M, Alwasel SH, Alhazza IM, Rhodes CJ, Valko M (2022). The effect of Luteolin on DNA damage mediated by a copper catalyzed Fenton reaction. J Inorg Biochem.

[R115] Joshi A, Rastedt W, Faber K, Schultz AG, Bulcke F, Dringen R (2016). Uptake and toxicity of copper oxide nanoparticles in C6 glioma cells. Neurochem Res.

[R116] Kamlin COF, T MJ, J LH, N SA (2024). Trientine tetrahydrochloride, from bench to bedside: a narrative review. Drugs.

[R117] Kang J, Jeong H, Jeong M, Kim J, Park S, Jung J, An JM, Kim D (2023). In situ activatable nitrobenzene-cysteine-copper(II) nano-complexes for programmed photodynamic cancer therapy. J Am Chem Soc.

[R118] Kang KR, Kim J, Ryu B, Lee SG, Oh MS, Baek J, Ren X, Canavero S, Kim CY, Chung HM (2021). BAPTA, a calcium chelator, neuroprotects injured neurons in vitro and promotes motor recovery after spinal cord transection in vivo. CNS Neurosci Ther.

[R119] Kardos J, Héja L, Simon Á, Jablonkai I, Kovács R, Jemnitz K (2018). Copper signalling: causes and consequences. Cell Commun Signal.

[R120] Kawahara M, Tanaka KI, Kato-Negishi M (2022). Crosstalk of copper and zinc in the pathogenesis of vascular dementia. J Clin Biochem Nutr.

[R121] Kawahara M, Tanaka KI, Kato-Negishi M (2024). Zinc, copper, and calcium: a triangle in the synapse for the pathogenesis of vascular-type senile dementia. Biomolecules.

[R122] Kelley KC, Grossman KF, Brittain-Blankenship M, Thorne KM, Akerley WL, Terrazas MC, Kosak KM, Boucher KM, Buys SS, McGregor KA, Werner TL, Agarwal N, Weis JR, Sharma S, Ward JH, Kennedy TP, Sborov DW, Shami PJ (2021). A Phase 1 dose-escalation study of disulfiram and copper gluconate in patients with advanced solid tumors involving the liver using S-glutathionylation as a biomarker. BMC Cancer.

[R123] Kerr JF, Wyllie AH, Currie AR (1972). Apoptosis: a basic biological phenomenon with wide-ranging implications in tissue kinetics. Br J Cancer.

[R124] Ketelut-Carneiro N, Fitzgerald KA (2022). Apoptosis, pyroptosis, and necroptosis-oh my! The many ways a cell can die. J Mol Biol.

[R125] Kijima K (2023). Zinc deficiency impairs axonal regeneration and functional recovery after spinal cord injury by modulating macrophage polarization via NF-κB pathway. Front Immunol.

[R126] Kim BE, Nevitt T, Thiele DJ (2008). Mechanisms for copper acquisition, distribution and regulation. Nat Chem Biol.

[R127] Kim J, Kundu M, Viollet B, Guan KL (2011). AMPK and mTOR regulate autophagy through direct phosphorylation of Ulk1. Nat Cell Biol.

[R128] Kirkland Z, Villasmil RJ, Alookaran J, Ward MC, Stone D (2022). Copper deficiency myeloneuropathy following Roux-en-Y gastric bypass in a 72-year-old female. Cureus.

[R129] Klevay LM (2013). Myelin and traumatic brain injury: the copper deficiency hypothesis. Med Hypotheses.

[R130] Knutson MD (2007). Steap proteins: implications for iron and copper metabolism. Nutr Rev.

[R131] Ko SH, Chen L (2025). Autophagy regulation and function in axon regeneration: new insights from Caenorhabditis elegans. Neural Regen Res.

[R132] Kobayashi S, Sasaki T, Katayama T, Hasegawa T, Nagano A, Sato K (2010). Temporal-spatial expression of presenilin 1 and the production of amyloid-beta after acute spinal cord injury in adult rat. Neurochem Int.

[R133] Kuhn S, Gritti L, Crooks D, Dombrowski Y (2019). Oligodendrocytes in development, myelin generation and beyond. Cells.

[R134] Kumar V, Singh AP, Wheeler N, Galindo CL, Kim JJ (2021). Safety profile of D-penicillamine: a comprehensive pharmacovigilance analysis by FDA adverse event reporting system. Expert Opin Drug Saf.

[R135] Lapenna D (2023). Glutathione and glutathione-dependent enzymes: from biochemistry to gerontology and successful aging. Ageing Res Rev.

[R136] Levenson CW (2005). Trace metal regulation of neuronal apoptosis: from genes to behavior. Physiol Behav.

[R137] Li C, Wu C, Ji C, Xu G, Chen J, Zhang J, Hong H, Liu Y, Cui Z (2023). The pathogenesis of DLD-mediated cuproptosis induced spinal cord injury and its regulation on immune microenvironment. Front Cell Neurosci.

[R138] Li F, Sun X, Sun K, Kong F, Jiang X, Kong Q (2024). Lupenone improves motor dysfunction in spinal cord injury mice through inhibiting the inflammasome activation and pyroptosis in microglia via the nuclear factor kappa B pathway. Neural Regen Res.

[R139] Li H, Wang J, Wu C, Wang L, Chen ZS, Cui W (2020). The combination of disulfiram and copper for cancer treatment. Drug Discov Today.

[R140] Li P, Sun Q, Bai S, Wang H, Zhao L (2024). Combination of the cuproptosis inducer disulfiram and anti-PD-L1 abolishes NSCLC resistance by ATP7B to regulate the HIF-1 signaling pathway. Int J Mol Med.

[R141] Li SR, Bu LL, Cai L (2022). Cuproptosis: lipoylated TCA cycle proteins-mediated novel cell death pathway. Signal Transduct Target Ther.

[R142] Li X, Ma Z, Mei L (2022). Cuproptosis-related gene SLC31A1 is a potential predictor for diagnosis, prognosis and therapeutic response of breast cancer. Am J Cancer Res.

[R143] Li Y, Du Y, Zhou Y, Chen Q, Luo Z, Ren Y, Chen X, Chen G (2023). Iron and copper: critical executioners of ferroptosis, cuproptosis and other forms of cell death. Cell Commun Signal.

[R144] Li Z, Wang G, Zhong S, Liao X, Lai S, Shan Y, Chen J, Zhang L, Lu Q, Shen S, Huang H, Zhang Y, Zhang L, Jia Y (2020). Alleviation of cognitive deficits and high copper levels by an NMDA receptor antagonist in a rat depression model. Compr Psychiatry.

[R145] Liao J, Hu Z, Li Q, Li H, Chen W, Huo H, Han Q, Zhang H, Guo J, Hu L, Pan J, Li Y, Tang Z (2022). Endoplasmic reticulum stress contributes to copper-induced pyroptosis via regulating the IRE1α-XBP1 pathway in pig jejunal epithelial cells. J Agric Food Chem.

[R146] Linder MC (2016). Ceruloplasmin and other copper binding components of blood plasma and their functions: an update. Metallomics.

[R147] Liu J, Liu Z, Wang W, Tian Y (2021). Real-time tracking and sensing of Cu(+) and Cu(2+) with a single sers probe in the live brain: toward understanding why copper ions were increased upon ischemia. Angew Chem Int Ed Engl.

[R148] Liu J, Tang H, Chen F, Li C, Xie Y, Kang R, Tang D (2024). NFE2L2 and SLC25A39 drive cuproptosis resistance through GSH metabolism. Sci Rep.

[R149] Liu K, Czaja MJ (2013). Regulation of lipid stores and metabolism by lipophagy. Cell Death Differ.

[R150] Liu M, Li H, Yang R, Ji D, Xia X (2022). GSK872 and necrostatin-1 protect retinal ganglion cells against necroptosis through inhibition of RIP1/RIP3/MLKL pathway in glutamate-induced retinal excitotoxic model of glaucoma. J Neuroinflammation.

[R151] Liu N, Chen M (2024). Crosstalk between ferroptosis and cuproptosis: From mechanism to potential clinical application. Biomed Pharmacother.

[R152] Liu T, Ma Z, Liu L, Pei Y, Wu Q, Xu S, Liu Y, Ding N, Guan Y, Zhang Y, Chen X (2024). Conditioned medium from human dental pulp stem cells treats spinal cord injury by inhibiting microglial pyroptosis. Neural Regen Res.

[R153] Liu Y, Luo G, Yan Y, Peng J (2022). A pan-cancer analysis of copper homeostasis-related gene lipoyltransferase 1: Its potential biological functions and prognosis values. Front Genet.

[R154] Liu Y, Shen T, Li Q, Yu X, Liu Y, Zhou C, Han J, Zhu Y (2025). Various gases for the treatment of neuropathic pain: mechanisms, current status, and future perspectives. Med Gas Res.

[R155] Liu Z, Yao X, Jiang W, Li W, Zhu S, Liao C, Zou L, Ding R, Chen J (2020). Advanced oxidation protein products induce microglia-mediated neuroinflammation via MAPKs-NF-κB signaling pathway and pyroptosis after secondary spinal cord injury. J Neuroinflammation.

[R156] Liu Z, Wang L, Xing Q, Liu X, Hu Y, Li W, Yan Q, Liu R, Huang N (2022). Identification of GLS as a cuproptosis-related diagnosis gene in acute myocardial infarction. Front Cardiovasc Med.

[R157] Lofstedt J, Jakowski R, Sharko P (1988). Enzootic ataxia and caprine arthritis/encephalitis virus infection in a New England goat herd [corrected]. J Am Vet Med Assoc.

[R158] Long JS, Ryan KM (2012). New frontiers in promoting tumour cell death: targeting apoptosis, necroptosis and autophagy. Oncogene.

[R159] Lu B, Gong X, Wang ZQ, Ding Y, Wang C, Luo TF, Piao MH, Meng FK, Chi GF, Luo YN, Ge PF (2017). Shikonin induces glioma cell necroptosis in vitro by ROS overproduction and promoting RIP1/RIP3 necrosome formation. Acta Pharmacol Sin.

[R160] Lu H, Zhou L, Zhang B, Xie Y, Yang H, Wang Z (2022). Cuproptosis key gene FDX1 is a prognostic biomarker and associated with immune infiltration in glioma. Front Med (Lausanne).

[R161] Lu L, Zhang Y, Shi W, Zhou Q, Lai Z, Pu Y, Yin L (2025). The role of autophagy in copper-induced apoptosis and developmental neurotoxicity in SH-SY5Y cells. Toxics.

[R162] Lutsenko S, Washington-Hughes C, Ralle M, Schmidt K (2019). Copper and the brain noradrenergic system. J Biol Inorg Chem.

[R163] Lynch SM, Frei B (1995). Reduction of copper, but not iron, by human low density lipoprotein (LDL). Implications for metal ion-dependent oxidative modification of LDL. J Biol Chem.

[R164] Maher P (2018). Potentiation of glutathione loss and nerve cell death by the transition metals iron and copper: Implications for age-related neurodegenerative diseases. Free Radic Biol Med.

[R165] Maiuri MC, Zalckvar E, Kimchi A, Kroemer G (2007). Self-eating and self-killing: crosstalk between autophagy and apoptosis. Nat Rev Mol Cell Biol.

[R166] Mancias JD, Wang X, Gygi SP, Harper JW, Kimmelman AC (2014). Quantitative proteomics identifies NCOA4 as the cargo receptor mediating ferritinophagy. Nature.

[R167] Maryon EB, Molloy SA, Kaplan JH (2013). Cellular glutathione plays a key role in copper uptake mediated by human copper transporter 1. Am J Physiol Cell Physiol.

[R168] Maryon EB, Molloy SA, Ivy K, Yu H, Kaplan JH (2013). Rate and regulation of copper transport by human copper transporter 1 (hCTR1). J Biol Chem.

[R169] Matsushima GK, Morell P (2001). The neurotoxicant, cuprizone, as a model to study demyelination and remyelination in the central nervous system. Brain Pathol.

[R170] Miao R (2023). Gasdermin D permeabilization of mitochondrial inner and outer membranes accelerates and enhances pyroptosis. Immunity.

[R171] Mirastschijski U, Martin A, Jorgensen LN, Sampson B, Ågren MS (2013). Zinc, copper, and selenium tissue levels and their relation to subcutaneous abscess, minor surgery, and wound healing in humans. Biol Trace Elem Res.

[R172] Mitra S, Keswani T, Ghosh N, Goswami S, Datta A, Das S, Maity S, Bhattacharyya A (2013). Copper induced immunotoxicity promote differential apoptotic pathways in spleen and thymus. Toxicology.

[R173] Monk BJ, Kauderer JT, Moxley KM, Bonebrake AJ, Dewdney SB, Secord AA, Ueland FR, Johnston CM, Aghajanian C (2018). A phase II evaluation of elesclomol sodium and weekly paclitaxel in the treatment of recurrent or persistent platinum-resistant ovarian, fallopian tube or primary peritoneal cancer: an NRG oncology/gynecologic oncology group study. Gynecol Oncol.

[R174] Morciano G, Giorgi C, Balestra D, Marchi S, Perrone D, Pinotti M, Pinton P (2016). Mcl-1 involvement in mitochondrial dynamics is associated with apoptotic cell death. Mol Biol Cell.

[R175] Mortazavi MM, Verma K, Harmon OA, Griessenauer CJ, Adeeb N, Theodore N, Tubbs RS (2015). The microanatomy of spinal cord injury: a review. Clin Anat.

[R176] Mukha A (2021). GLS-driven glutamine catabolism contributes to prostate cancer radiosensitivity by regulating the redox state, stemness and ATG5-mediated autophagy. Theranostics.

[R177] Mukhopadhyay CK, Fox PL (1998). Ceruloplasmin copper induces oxidant damage by a redox process utilizing cell-derived superoxide as reductant. Biochemistry.

[R178] Mutti C, Bazzurri V, Tsantes E, Curti E, Parrino L, Granella F (2021). Copper deficiency-associated myelopathy in cryptogenic hyperzincemia: a case report. Acta Biomed.

[R179] Myers BM, Prendergast FG, Holman R, Kuntz SM, Larusso NF (1993). Alterations in hepatocyte lysosomes in experimental hepatic copper overload in rats. Gastroenterology.

[R180] Myint ZW, Oo TH, Thein KZ, Tun AM, Saeed H (2018). Copper deficiency anemia: review article. Ann Hematol.

[R181] Naeve GS, Vana AM, Eggold JR, Kelner GS, Maki R, Desouza EB, Foster AC (1999). Expression profile of the copper homeostasis gene, rAtox1, in the rat brain. Neuroscience.

[R182] Narayan SK, Kaveer N (2006). CNS demyelination due to hypocupremia in Wilson’s disease from overzealous treatment. Neurol India.

[R183] Nechushtan H, Hamamreh Y, Nidal S, Gotfried M, Baron A, Shalev YI, Nisman B, Peretz T, Peylan-Ramu N (2015). A phase IIb trial assessing the addition of disulfiram to chemotherapy for the treatment of metastatic non-small cell lung cancer. Oncologist.

[R184] Niciu MJ, Ma XM, El Meskini R, Ronnett GV, Mains RE, Eipper BA (2006). Developmental changes in the expression of ATP7A during a critical period in postnatal neurodevelopment. Neuroscience.

[R185] Nishihara E, Furuyama T, Yamashita S, Mori N (1998). Expression of copper trafficking genes in the mouse brain. Neuroreport.

[R186] Nose Y, Kim BE, Thiele DJ (2006). Ctr1 drives intestinal copper absorption and is essential for growth, iron metabolism, and neonatal cardiac function. Cell Metab.

[R187] O’Day S, Gonzalez R, Lawson D, Weber R, Hutchins L, Anderson C, Haddad J, Kong S, Williams A, Jacobson E (2009). Phase II, randomized, controlled, double-blinded trial of weekly elesclomol plus paclitaxel versus paclitaxel alone for stage IV metastatic melanoma. J Clin Oncol.

[R188] O’Day SJ, Eggermont AM, Chiarion-Sileni V, Kefford R, Grob JJ, Mortier L, Robert C, Schachter J, Testori A, Mackiewicz J, Friedlander P, Garbe C, Ugurel S, Collichio F, Guo W, Lufkin J, Bahcall S, Vukovic V, Hauschild A (2013). Final results of phase III SYMMETRY study: randomized, double-blind trial of elesclomol plus paclitaxel versus paclitaxel alone as treatment for chemotherapy-naive patients with advanced melanoma. J Clin Oncol.

[R189] Orr MB, Gensel JC (2018). Spinal cord injury scarring and inflammation: therapies targeting glial and inflammatory responses. Neurotherapeutics.

[R190] Ossola JO, Groppa MD, Tomaro ML (1997). Relationship between oxidative stress and heme oxygenase induction by copper sulfate. Arch Biochem Biophys.

[R191] Ouyang L, Shi Z, Zhao S, Wang FT, Zhou TT, Liu B, Bao JK (2012). Programmed cell death pathways in cancer: a review of apoptosis, autophagy and programmed necrosis. Cell Prolif.

[R192] Pan Q, Kleer CG, van Golen KL, Irani J, Bottema KM, Bias C, De Carvalho M, Mesri EA, Robins DM, Dick RD, Brewer GJ, Merajver SD (2002). Copper deficiency induced by tetrathiomolybdate suppresses tumor growth and angiogenesis. Cancer Res.

[R193] Pan Y, Yu Y, Wang X, Zhang T (2020). Tumor-associated macrophages in tumor immunity. Front Immunol.

[R194] Patel D, Kell A, Simard B, Xiang B, Lin HY, Tian G (2011). The cell labeling efficacy, cytotoxicity and relaxivity of copper-activated MRI/PET imaging contrast agents. Biomaterials.

[R195] Patwa J, Thakur A, Flora SJS (2022). Alpha lipoic acid and monoisoamyl-DMSA combined treatment ameliorates copper-induced neurobehavioral deficits, oxidative stress, and inflammation. Toxics.

[R196] Peters C, Muñoz B, Sepúlveda FJ, Urrutia J, Quiroz M, Luza S, De Ferrari GV, Aguayo LG, Opazo C (2011). Biphasic effects of copper on neurotransmission in rat hippocampal neurons. J Neurochem.

[R197] Philipp TM, Gernoth L, Will A, Schwarz M, Ohse VA, Kipp AP, Steinbrenner H, Klotz LO (2023). Selenium-binding protein 1 (SELENBP1) is a copper-dependent thiol oxidase. Redox Biol.

[R198] Pourahmad J, O’Brien PJ (2000). A comparison of hepatocyte cytotoxic mechanisms for Cu2+ and Cd2+. Toxicology.

[R199] Powell SR (2000). The antioxidant properties of zinc. J Nutr.

[R200] Prerna K, Dubey VK (2022). Beclin1-mediated interplay between autophagy and apoptosis: new understanding. Int J Biol Macromol.

[R201] Qian J, Ji L, Xu W, Hou G, Wang J, Wang Y, Wang T (2022). Copper-hydrazide coordinated multifunctional hyaluronan hydrogels for infected wound healing. ACS Appl Mater Interfaces.

[R202] Quan J, Chang X, Liu S, He T, Zhong G, Liu Z, Yu W (2025). Long-term copper exposure induced pyroptosis and inflammation of rat spleen through intestinal-splenic axis. Ecotoxicol Environ Saf.

[R203] Que EL, Domaille DW, Chang CJ (2008). Metals in neurobiology: probing their chemistry and biology with molecular imaging. Chem Rev.

[R204] Rafe MR, Saha P, Bello ST (2024). Targeting NMDA receptors with an antagonist is a promising therapeutic strategy for treating neurological disorders. Behav Brain Res.

[R205] Raveh O, Pinchuk I, Schnitzer E, Fainaru M, Schaffer Z, Lichtenberg D (2000). Kinetic analysis of copper-induced peroxidation of HDL, autoaccelerated and tocopherol-mediated peroxidation. Free Radic Biol Med.

[R206] Ren X, Li Y, Zhou Y, Hu W, Yang C, Jing Q, Zhou C, Wang X, Hu J, Wang L, Yang J, Wang H, Xu H, Li H, Tong X, Wang Y, Du J (2021). Overcoming the compensatory elevation of NRF2 renders hepatocellular carcinoma cells more vulnerable to disulfiram/copper-induced ferroptosis. Redox Biol.

[R207] Rhee YS, Burnham K, Stoecker BJ, Lucas E (2004). Effects of chromium and copper depletion on lymphocyte reactivity to mitogens in diabetes-prone BHE/cdb rats. Nutrition.

[R208] Rice TM, Clarke RW, Godleski JJ, Al-Mutairi E, Jiang NF, Hauser R, Paulauskis JD (2001). Differential ability of transition metals to induce pulmonary inflammation. Toxicol Appl Pharmacol.

[R209] Rong Y, Ji C, Wang Z, Ge X, Wang J, Ye W, Tang P, Jiang D, Fan J, Yin G, Liu W, Cai W (2021). Small extracellular vesicles encapsulating CCL2 from activated astrocytes induce microglial activation and neuronal apoptosis after traumatic spinal cord injury. J Neuroinflammation.

[R210] Salsabili N, Mehrsai AR, Jalaie S (2009). Concentration of blood and seminal plasma elements and their relationships with semen parameters in men with spinal cord injury. Andrologia.

[R211] Sayre LM, Perry G, Harris PL, Liu Y, Schubert KA, Smith MA (2000). In situ oxidative catalysis by neurofibrillary tangles and senile plaques in Alzheimer’s disease: a central role for bound transition metals. J Neurochem.

[R212] Scheijen EEM, Hendrix S, Wilson DM (2022). Oxidative DNA damage in the pathophysiology of spinal cord injury: seems obvious, but where is the evidence?. Antioxidants (Basel).

[R213] Schlief ML, Gitlin JD (2006). Copper homeostasis in the CNS: a novel link between the NMDA receptor and copper homeostasis in the hippocampus. Mol Neurobiol.

[R214] Schlief ML, Craig AM, Gitlin JD (2005). NMDA receptor activation mediates copper homeostasis in hippocampal neurons. J Neurosci.

[R215] Schmidt K, Ralle M, Schaffer T, Jayakanthan S, Bari B, Muchenditsi A, Lutsenko S (2018). ATP7A and ATP7B copper transporters have distinct functions in the regulation of neuronal dopamine-β-hydroxylase. J Biol Chem.

[R216] Schneider BJ, Lee JS, Hayman JA, Chang AC, Orringer MB, Pickens A, Pan CC, Merajver SD, Urba SG (2013). Pre-operative chemoradiation followed by post-operative adjuvant therapy with tetrathiomolybdate, a novel copper chelator, for patients with resectable esophageal cancer. Invest New Drugs.

[R217] Seelig J, Heller RA, Hackler J, Haubruck P, Moghaddam A, Biglari B, Schomburg L (2020). Selenium and copper status - potential signposts for neurological remission after traumatic spinal cord injury. J Trace Elem Med Biol.

[R218] Seelig J, Heller RA, Haubruck P, Sun Q, Georg Klingenberg J, Hackler J, Crowell HL, Daniel V, Moghaddam A, Schomburg L, Biglari B (2021). Selenium-binding protein 1 (SELENBP1) as biomarker for adverse clinical outcome after traumatic spinal cord injury. Front Neurosci.

[R219] Seo J, Seong D, Nam YW, Hwang CH, Lee SR, Lee CS, Jin Y, Lee HW, Oh DB, Vandenabeele P, Song J (2020). Beclin 1 functions as a negative modulator of MLKL oligomerisation by integrating into the necrosome complex. Cell Death Differ.

[R220] Sheftel AD, Stehling O, Pierik AJ, Elsässer HP, Mühlenhoff U, Webert H, Hobler A, Hannemann F, Bernhardt R, Lill R (2010). Humans possess two mitochondrial ferredoxins, Fdx1 and Fdx2, with distinct roles in steroidogenesis, heme, and Fe/S cluster biosynthesis. Proc Natl Acad Sci U S A.

[R221] Shen Y, Cao X, Lu M, Gu H, Li M, Posner DA (2022). Current treatments after spinal cord injury: cell engineering, tissue engineering, and combined therapies. Smart Med.

[R222] Shi W, Zhou Q, Lu L, Zhang Y, Zhang H, Pu Y, Yin L (2024). Copper induced cytosolic escape of mitochondrial DNA and activation of cGAS-STING-NLRP3 pathway-dependent pyroptosis in C8-D1A cells. Ecotoxicol Environ Saf.

[R223] Shi Z, Yuan S, Shi L, Li J, Ning G, Kong X, Feng S (2021). Programmed cell death in spinal cord injury pathogenesis and therapy. Cell Prolif.

[R224] Shimizu EN, Seifert JL, Johnson KJ, Romero-Ortega MI (2018). Prophylactic riluzole attenuates oxidative stress damage in spinal cord distraction. J Neurotrauma.

[R225] Sies H, Jones DP (2020). Reactive oxygen species (ROS) as pleiotropic physiological signalling agents. Nat Rev Mol Cell Biol.

[R226] Skjørringe T, Burkhart A, Johnsen KB, Moos T (2015). Divalent metal transporter 1 (DMT1) in the brain: implications for a role in iron transport at the blood-brain barrier, and neuronal and glial pathology. Front Mol Neurosci.

[R227] Socha P, Czlonkowska A, Janczyk W, Litwin T (2022). Wilson’s disease- management and long term outcomes. Best Pract Res Clin Gastroenterol.

[R228] Sokol RJ, Devereaux M, Mierau GW, Hambidge KM, Shikes RH (1990). Oxidant injury to hepatic mitochondrial lipids in rats with dietary copper overload. Modification by vitamin E deficiency. Gastroenterology.

[R229] Solier S (2023). A druggable copper-signalling pathway that drives inflammation. Nature.

[R230] Song M, Kim SH, Im CY, Hwang HJ (2018). Recent development of small molecule glutaminase inhibitors. Curr Top Med Chem.

[R231] Song X, Zhu S, Chen P, Hou W, Wen Q, Liu J, Xie Y, Liu J, Klionsky DJ, Kroemer G, Lotze MT, Zeh HJ, Kang R, Tang D (2018). AMPK-mediated BECN1 phosphorylation promotes ferroptosis by directly blocking system X(c)(-) activity. Curr Biol.

[R232] Springer JE, Azbill RD, Knapp PE (1999). Activation of the caspase-3 apoptotic cascade in traumatic spinal cord injury. Nat Med.

[R233] Squitti R, Salustri C, Rongioletti M, Siotto M (2017). Commentary: The case for abandoning therapeutic chelation of copper ions in Alzheimer’s disease. Front Neurol.

[R234] Stadtman ER (1990). Metal ion-catalyzed oxidation of proteins: biochemical mechanism and biological consequences. Free Radic Biol Med.

[R235] Steinberg D (1997). Low density lipoprotein oxidation and its pathobiological significance. J Biol Chem.

[R236] Stowe RC, Sun Q, Elsea SH, Scaglia F (2018). LIPT1 deficiency presenting as early infantile epileptic encephalopathy, Leigh disease, and secondary pyruvate dehydrogenase complex deficiency. Am J Med Genet A.

[R237] Su L, Zhang J, Gomez H, Kellum JA, Peng Z (2023). Mitochondria ROS and mitophagy in acute kidney injury. Autophagy.

[R238] Sun X, Wang X, Chen T, Li T, Cao K, Lu A, Chen Y, Sun D, Luo J, Fan J, Young W, Ren Y (2010). Myelin activates FAK/Akt/NF-kappaB pathways and provokes CR3-dependent inflammatory response in murine system. PLoS One.

[R239] Sun Y, Qiao Y, Liu Y, Zhou J, Wang X, Zheng H, Xu Z, Zhang J, Zhou Y, Qian L, Zhang C, Lou H (2021). ent-Kaurane diterpenoids induce apoptosis and ferroptosis through targeting redox resetting to overcome cisplatin resistance. Redox Biol.

[R240] Suraweera CD, Banjara S, Hinds MG, Kvansakul M (2022). Metazoans and intrinsic apoptosis: an evolutionary analysis of the Bcl-2 family. Int J Mol Sci.

[R241] Tang D, Chen X, Kroemer G (2022). Cuproptosis: a copper-triggered modality of mitochondrial cell death. Cell Res.

[R242] Tang PM, Nikolic-Paterson DJ, Lan HY (2019). Macrophages: versatile players in renal inflammation and fibrosis. Nat Rev Nephrol.

[R243] Tang S, Bai L, Hou W, Hu Z, Chen X, Zhao J, Liang C, Zhang W, Duan Z, Zheng S (2022). Comparison of the effectiveness and safety of d-penicillamine and zinc salt treatment for symptomatic Wilson disease: a systematic review and meta-analysis. Front Pharmacol.

[R244] Tao J, Zhou J, Zhu H, Xu L, Yang J, Mu X, Fan X (2024). Tetramethylpyrazine inhibits ferroptosis in spinal cord injury by regulating iron metabolism through the NRF2/ARE pathway. Front Pharmacol.

[R245] Tarin M, Babaie M, Eshghi H, Matin MM, Saljooghi AS (2023). Elesclomol, a copper-transporting therapeutic agent targeting mitochondria: from discovery to its novel applications. J Transl Med.

[R246] Teleanu DM, Niculescu AG, Lungu II, Radu CI, Vladâcenco O, Roza E, Costăchescu B, Grumezescu AM, Teleanu RI (2022). An Overview of Oxidative Stress, Neuroinflammation, and Neurodegenerative Diseases. Int J Mol Sci.

[R247] Teles M, Mackenzie S, Boltaña S, Callol A, Tort L (2011). Gene expression and TNF-alpha secretion profile in rainbow trout macrophages following exposures to copper and bacterial lipopolysaccharide. Fish Shellfish Immunol.

[R248] Teschke R, Eickhoff A (2024). Wilson disease: copper-mediated cuproptosis, iron-related ferroptosis, and clinical highlights, with comprehensive and critical analysis update. Int J Mol Sci.

[R249] Tian Z, Jiang S, Zhou J, Zhang W (2023). Copper homeostasis and cuproptosis in mitochondria. Life Sci.

[R250] Tindel NL, Marcillo AE, Tay BK, Bunge RP, Eismont FJ (2001). The effect of surgically implanted bullet fragments on the spinal cord in a rabbit model. J Bone Joint Surg Am.

[R251] Torii S, Shintoku R, Kubota C, Yaegashi M, Torii R, Sasaki M, Suzuki T, Mori M, Yoshimoto Y, Takeuchi T, Yamada K (2016). An essential role for functional lysosomes in ferroptosis of cancer cells. Biochem J.

[R252] Travaglia A, La Mendola D, Magrì A, Nicoletti VG, Pietropaolo A, Rizzarelli E (2012). Copper, BDNF and its N-terminal domain: inorganic features and biological perspectives. Chemistry.

[R253] Travaglia A, Arena G, Fattorusso R, Isernia C, La Mendola D, Malgieri G, Nicoletti VG, Rizzarelli E (2011). The inorganic perspective of nerve growth factor: interactions of Cu2+ and Zn2+ with the N-terminus fragment of nerve growth factor encompassing the recognition domain of the TrkA receptor. Chemistry.

[R254] Trejo-Solís C, Jimenez-Farfan D, Rodriguez-Enriquez S, Fernandez-Valverde F, Cruz-Salgado A, Ruiz-Azuara L, Sotelo J (2012). Copper compound induces autophagy and apoptosis of glioma cells by reactive oxygen species and JNK activation. BMC Cancer.

[R255] Trumbore CN, Ehrlich RS, Myers YN (2001). Changes in DNA conformation induced by gamma irradiation in the presence of copper. Radiat Res.

[R256] Tsuchiya K, Nakajima S, Hosojima S, Thi Nguyen D, Hattori T, Manh Le T, Hori O, Mahib MR, Yamaguchi Y, Miura M, Kinoshita T, Kushiyama H, Sakurai M, Shiroishi T, Suda T (2019). Caspase-1 initiates apoptosis in the absence of gasdermin D. Nat Commun.

[R257] Tsvetkov P, Coy S, Petrova B, Dreishpoon M, Verma A, Abdusamad M, Rossen J, Joesch-Cohen L, Humeidi R, Spangler RD, Eaton JK, Frenkel E, Kocak M, Corsello SM, Lutsenko S, Kanarek N, Santagata S, Golub TR (2022). Copper induces cell death by targeting lipoylated TCA cycle proteins. Science.

[R258] Tural K, Ozden O, Bilgi Z, Kubat E, Ermutlu CS, Merhan O, Tasoglu I (2021). The protective effect of betanin and copper on spinal cord ischemia-reperfusion injury. J Spinal Cord Med.

[R259] Valko M, Rhodes CJ, Moncol J, Izakovic M, Mazur M (2006). Free radicals, metals and antioxidants in oxidative stress-induced cancer. Chem Biol Interact.

[R260] Van Broeckhoven J, Sommer D, Dooley D, Hendrix S, Franssen A (2021). Macrophage phagocytosis after spinal cord injury: when friends become foes. Brain.

[R261] Vogel FS, Evans JW (1961). Morphologic alterations produced by copper in neural tissues with consideration of the role of the metal in the pathogenesis of Wilson’s disease. J Exp Med.

[R262] Vogt KC (1842). Untersuchungen über die entwicklungsgeschichte der geburtshelferkroete (alytes obstetricans).

[R263] Vringer E, Tait SWG (2023). Mitochondria and cell death-associated inflammation. Cell Death Differ.

[R264] Wan S, Zhang G, Liu R, Abbas MN, Cui H (2023). Pyroptosis, ferroptosis, and autophagy cross-talk in glioblastoma opens up new avenues for glioblastoma treatment. Cell Commun Signal.

[R265] Wang C, Ma C, Gong L, Guo Y, Fu K, Zhang Y, Zhou H, Li Y (2021). Macrophage polarization and its role in liver disease. Front Immunol.

[R266] Wang C, Zhang L, Ndong JC, Hettinghouse A, Sun G, Chen C, Zhang C, Liu R, Liu CJ (2019). Progranulin deficiency exacerbates spinal cord injury by promoting neuroinflammation and cell apoptosis in mice. J Neuroinflammation.

[R267] Wang H, Lv G, Lian S, Wang J, Wu R (2021). Effect of copper, zinc, and selenium on the migration of bovine neutrophils. Vet Sci.

[R268] Wang H, Zhang R, Shen J, Jin Y, Chang C, Hong M, Guo S, He D (2023). Circulating level of blood iron and copper associated with inflammation and disease activity of rheumatoid arthritis. Biol Trace Elem Res.

[R269] Wang W, Chen Z, Hua Y (2023). Bioinformatics prediction and experimental validation identify a novel cuproptosis-related gene signature in human synovial inflammation during osteoarthritis progression. Biomolecules.

[R270] Wang Y, Zhu S, Hodgkinson V, Prohaska JR, Weisman GA, Gitlin JD, Petris MJ (2012). Maternofetal and neonatal copper requirements revealed by enterocyte-specific deletion of the Menkes disease protein. Am J Physiol Gastrointest Liver Physiol.

[R271] Wang Y, Li D, Xu K, Wang G, Zhang F (2025). Copper homeostasis and neurodegenerative diseases. Neural Regen Res.

[R272] Wei MC, Zong WX, Cheng EH, Lindsten T, Panoutsakopoulou V, Ross AJ, Roth KA, MacGregor GR, Thompson CB, Korsmeyer SJ (2001). Proapoptotic BAX and BAK: a requisite gateway to mitochondrial dysfunction and death. Science.

[R273] Weiss KH, Thurik F, Gotthardt DN, Schäfer M, Teufel U, Wiegand F, Merle U, Ferenci-Foerster D, Maieron A, Stauber R, Zoller H, Schmidt HH, Reuner U, Hefter H, Trocello JM, Houwen RH, Ferenci P, Stremmel W, EUROWILSON Consortium (2013). Efficacy and safety of oral chelators in treatment of patients with Wilson disease. Clin Gastroenterol Hepatol.

[R274] Wen MH, Xie X, Huang PS, Yang K, Chen TY (2021). Crossroads between membrane trafficking machinery and copper homeostasis in the nerve system. Open Biol.

[R275] Wen S, Li Y, Shen X, Wang Z, Zhang K, Zhang J, Mei X (2021). Protective effects of zinc on spinal cord injury. J Mol Neurosci.

[R276] Westphal D, Kluck RM, Dewson G (2014). Building blocks of the apoptotic pore: how Bax and Bak are activated and oligomerize during apoptosis. Cell Death Differ.

[R277] White AR, Huang X, Jobling MF, Barrow CJ, Beyreuther K, Masters CL, Bush AI, Cappai R (2001). Homocysteine potentiates copper- and amyloid beta peptide-mediated toxicity in primary neuronal cultures: possible risk factors in the Alzheimer’s-type neurodegenerative pathways. J Neurochem.

[R278] White C, Lee J, Kambe T, Fritsche K, Petris MJ (2009). A role for the ATP7A copper-transporting ATPase in macrophage bactericidal activity. J Biol Chem.

[R279] Winkler J, Rand ML, Schmugge M, Speer O (2013). Omi/HtrA2 and XIAP are components of platelet apoptosis signalling. Thromb Haemost.

[R280] Wu C, Wang L, Chen S, Shi L, Liu M, Tu P, Sun J, Zhao R, Zhang Y, Wang J, Pan Y, Ma Y, Guo Y (2023). Iron induces B cell pyroptosis through Tom20-Bax-caspase-gasdermin E signaling to promote inflammation post-spinal cord injury. J Neuroinflammation.

[R281] Wu Y, Shen L, Wang R, Tang J, Ding SQ, Wang SN, Guo XY, Hu JG, Lü HZ (2018). Increased ceruloplasmin expression caused by infiltrated leukocytes, activated microglia, and astrocytes in injured female rat spinal cords. J Neurosci Res.

[R282] Xiao J, Wang C, Yao JC, Alippe Y, Xu C, Kress D, Civitelli R, Abu-Amer Y, Kanneganti TD, Link DC, Mbalaviele G (2018). Gasdermin D mediates the pathogenesis of neonatal-onset multisystem inflammatory disease in mice. PLoS Biol.

[R283] Xiao T, Liu J, Li Y, Cai Y, Xing X, Shao M, Zhang C, Duan D, Liu S, Tan G, Wang L, Wu Z, Gong Z, Zhou L (2023). Microenvironment-responsive Cu-phenolic networks coated nanofibrous dressing with timely macrophage phenotype transition for chronic MRSA infected wound healing. Mater Today Bio.

[R284] Xiao Y, Zhang T, Ma X, Yang QC, Yang LL, Yang SC, Liang M, Xu Z, Sun ZJ (2021). Microenvironment-responsive prodrug-induced pyroptosis boosts cancer immunotherapy. Adv Sci (Weinh).

[R285] Xie J, Yang Y, Gao Y, He J (2023). Cuproptosis: mechanisms and links with cancers. Mol Cancer.

[R286] Xiong W, Li C, Kong G, Zeng Q, Wang S, Yin G, Gu J, Fan J (2022). Treg cell-derived exosomes miR-709 attenuates microglia pyroptosis and promotes motor function recovery after spinal cord injury. J Nanobiotechnology.

[R287] Xu P, Xu J, Liu S, Yang Z (2012). Nano copper induced apoptosis in podocytes via increasing oxidative stress. J Hazard Mater.

[R288] Xu S, Wang J, Zhong J, Shao M, Jiang J, Song J, Zhu W, Zhang F, Xu H, Xu G, Zhang Y, Ma X, Lyu F (2021). CD73 alleviates GSDMD-mediated microglia pyroptosis in spinal cord injury through PI3K/AKT/Foxo1 signaling. Clin Transl Med.

[R289] Xue Q, Kang R, Klionsky DJ, Tang D, Liu J, Chen X (2023). Copper metabolism in cell death and autophagy. Autophagy.

[R290] Xue Q, Yan D, Chen X, Li X, Kang R, Klionsky DJ, Kroemer G, Chen X, Tang D, Liu J (2023). Copper-dependent autophagic degradation of GPX4 drives ferroptosis. Autophagy.

[R291] Xue X, Hu Y, Wang S, Chen X, Jiang Y, Su J (2022). Fabrication of physical and chemical crosslinked hydrogels for bone tissue engineering. Bioact Mater.

[R292] Yang F, Pei R, Zhang Z, Liao J, Yu W, Qiao N, Han Q, Li Y, Hu L, Guo J, Pan J, Tang Z (2019). Copper induces oxidative stress and apoptosis through mitochondria-mediated pathway in chicken hepatocytes. Toxicol In Vitro.

[R293] Yang F, Liao J, Yu W, Qiao N, Guo J, Han Q, Li Y, Hu L, Pan J, Tang Z (2021). Exposure to copper induces mitochondria-mediated apoptosis by inhibiting mitophagy and the PINK1/parkin pathway in chicken (Gallus gallus) livers. J Hazard Mater.

[R294] Yang M, Chen P, Liu J, Zhu S, Kroemer G, Klionsky DJ, Lotze MT, Zeh HJ, Kang R, Tang D (2019). Clockophagy is a novel selective autophagy process favoring ferroptosis. Sci Adv.

[R295] Yang M (2022). COMMD10 inhibits HIF1α/CP loop to enhance ferroptosis and radiosensitivity by disrupting Cu-Fe balance in hepatocellular carcinoma. J Hepatol.

[R296] Yang S, Li X, Yan J, Jiang F, Fan X, Jin J, Zhang W, Zhong D, Li G (2024). Disulfiram downregulates ferredoxin 1 to maintain copper homeostasis and inhibit inflammation in cerebral ischemia/reperfusion injury. Sci Rep.

[R297] Yang W, Guo Q, Wu H, Tong L, Xiao J, Wang Y, Liu R, Xu L, Yan H, Sun Z (2023). Comprehensive analysis of the cuproptosis-related gene DLD across cancers: a potential prognostic and immunotherapeutic target. Front Pharmacol.

[R298] Yang Y, Xu C, Xu S, Li Y, Chen K, Yang T, Bao J, Xu Y, Chen J, Mao C, Chen L, Sun W (2025). Injectable hydrogels activated with copper sulfide nanoparticles for enhancing spatiotemporal sterilization and osteogenesis in periodontal therapy. Biomater Sci.

[R299] Yang Z, Wang Y, Zhang Y, He X, Zhong CQ, Ni H, Chen X, Liang Y, Wu J, Zhao S, Zhou D, Han J (2018). RIP3 targets pyruvate dehydrogenase complex to increase aerobic respiration in TNF-induced necroptosis. Nat Cell Biol.

[R300] Yao X, Zhang Y, Hao J, Duan HQ, Zhao CX, Sun C, Li B, Fan BY, Wang X, Li WX, Fu XH, Hu Y, Liu C, Kong XH, Feng SQ (2019). Deferoxamine promotes recovery of traumatic spinal cord injury by inhibiting ferroptosis. Neural Regen Res.

[R301] Ye Z, Zhang S, Cai J, Ye L, Gao L, Wang Y, Tong S, Sun Q, Wu Y, Xiong X, Chen Q (2022). Development and validation of cuproptosis-associated prognostic signatures in WHO 2/3 glioma. Front Oncol.

[R302] Yi X, Kim K, Yuan W, Xu L, Kim HS, Homeister JW, Key NS, Maeda N (2009). Mice with heterozygous deficiency of lipoic acid synthase have an increased sensitivity to lipopolysaccharide-induced tissue injury. J Leukoc Biol.

[R303] Yu F, Gong P, Hu Z, Qiu Y, Cui Y, Gao X, Chen H, Li J (2015). Cu(II) enhances the effect of Alzheimer’s amyloid-β peptide on microglial activation. J Neuroinflammation.

[R304] Yu M, Wang Z, Wang D, Aierxi M, Ma Z, Wang Y (2023). Oxidative stress following spinal cord injury: from molecular mechanisms to therapeutic targets. J Neurosci Res.

[R305] Yu Z, Zhou R, Zhao Y, Pan Y, Liang H, Zhang JS, Tai S, Jin L, Teng CB (2019). Blockage of SLC31A1-dependent copper absorption increases pancreatic cancer cell autophagy to resist cell death. Cell Prolif.

[R306] Zhang C, Zeng Y, Guo X, Shen H, Zhang J, Wang K, Ji M, Huang S (2022). Pan-cancer analyses confirmed the cuproptosis-related gene FDX1 as an immunotherapy predictor and prognostic biomarker. Front Genet.

[R307] Zhang D, Wang M, Huang X, Wang L, Liu Y, Zhou S, Tang Y, Wang Q, Li Z, Wang G (2023). GLS as a diagnostic biomarker in breast cancer: in-silico, in-situ, and in-vitro insights. Front Oncol.

[R308] Zhang H, Sun C, He B, Zhang X, Hao H, Hou Y, Li A, Wang Y, Wang Y (2023). Macrophage migration inhibitory factor promotes expression of matrix metalloproteinases 1 and 3 in spinal cord astrocytes following gecko tail amputation. J Integr Neurosci.

[R309] Zhang L, Cui T, Wang X (2023). The interplay between autophagy and regulated necrosis. Antioxid Redox Signal.

[R310] Zhang N, Ji C, Peng X, Tang M, Bao X, Yuan C (2022). Bioinformatics analysis identified immune infiltration, risk and drug prediction models of copper-induced death genes involved in salivary glands damage of primary Sjögren’s syndrome. Medicine (Baltimore).

[R311] Zhang P, Yang H, Zhu K, Chang C, Lv W, Li R, Li X, Ye T, Cao D (2023). SLC31A1 identifying a novel biomarker with potential prognostic and immunotherapeutic potential in pan-cancer. Biomedicines.

[R312] Zhang SS, Noordin MM, Rahman SO, Haron J (2000). Effects of copper overload on hepatic lipid peroxidation and antioxidant defense in rats. Vet Hum Toxicol.

[R313] Zhang T, Kephart J, Bronson E, Anand M, Daly C, Spasojevic I, Bakthavatsalam S, Franz K, Berg H, Karachaliou GS, James OG, Howard L, Halabi S, Harrison MR, Armstrong AJ, George DJ (2022). Prospective clinical trial of disulfiram plus copper in men with metastatic castration-resistant prostate cancer. Prostate.

[R314] Zhang Y, Jia Q, Li J, Wang J, Liang K, Xue X, Chen T, Kong L, Ren H, Liu W, Wang P, Ge J (2023). Copper-bacteriochlorin nanosheet as a specific pyroptosis inducer for robust tumor immunotherapy. Adv Mater.

[R315] Zhang Y, Zhang Z, Mo Y, Zhang Y, Yuan J, Zhang Q (2024). MMP-3 mediates copper oxide nanoparticle-induced pulmonary inflammation and fibrosis. J Nanobiotechnology.

[R316] Zhao H, Wang Y, Shao Y, Liu J, Wang S, Xing M (2018). Oxidative stress-induced skeletal muscle injury involves in NF-κB/p53-activated immunosuppression and apoptosis response in copper (II) or/and arsenite-exposed chicken. Chemosphere.

[R317] Zhao X, Chen J, Yin S, Shi J, Zheng M, He C, Meng H, Han Y, Han J, Guo J, Yuan Z, Wang Y (2022). The expression of cuproptosis-related genes in hepatocellular carcinoma and their relationships with prognosis. Front Oncol.

[R318] Zhao Y, Xia Q, Zong H, Wang Y, Dong H, Zhu L, Xia J, Mao Q, Weng Z, Liao W, Xin Z (2023). Bibliometric and visual analysis of spinal cord injury-associated macrophages from 2002 to 2023. Front Neurol.

[R319] Zheng J, Chen T, Wang K, Peng C, Zhao M, Xie Q, Li B, Lin H, Zhao Z, Ji Z, Tang BZ, Liao Y (2024). Engineered multifunctional zinc-organic framework-based aggregation-induced emission nanozyme for accelerating spinal cord injury recovery. ACS Nano.

[R320] Zheng Q, Wang D, Lin R, Xu W (2025). Pyroptosis, ferroptosis, and autophagy in spinal cord injury: regulatory mechanisms and therapeutic targets. Neural Regen Res.

[R321] Zhong CC, Zhao T, Hogstrand C, Chen F, Song CC, Luo Z (2022). Copper (Cu) induced changes of lipid metabolism through oxidative stress-mediated autophagy and Nrf2/PPARγ pathways. J Nutr Biochem.

[R322] Zhou B, Liu J, Kang R, Klionsky DJ, Kroemer G, Tang D (2020). Ferroptosis is a type of autophagy-dependent cell death. Semin Cancer Biol.

[R323] Zhou H, Li Z, Jing S, Wang B, Ye Z, Xiong W, Liu Y, Liu Y, Xu C, Kumeria T, He Y, Ye Q (2024). Repair spinal cord injury with a versatile anti-oxidant and neural regenerative nanoplatform. J Nanobiotechnology.

[R324] Zhou K, Zheng Z, Li Y, Han W, Zhang J, Mao Y, Chen H, Zhang W, Liu M, Xie L, Zhang H, Xu H, Xiao J (2020). TFE3, a potential therapeutic target for Spinal Cord Injury via augmenting autophagy flux and alleviating ER stress. Theranostics.

[R325] Zhou L, Ouyang L, Lin S, Chen S, Liu Y, Zhou W, Wang X (2018). Protective role of β-carotene against oxidative stress and neuroinflammation in a rat model of spinal cord injury. Int Immunopharmacol.

[R326] Zhou Q, Zhang Y, Lu L, Zhang H, Zhao C, Pu Y, Yin L (2022). Copper induces microglia-mediated neuroinflammation through ROS/NF-κB pathway and mitophagy disorder. Food Chem Toxicol.

[R327] Zhou W, Zhang H, Huang L, Sun C, Yue Y, Cao X, Jia H, Wang C, Gao Y (2023). Disulfiram with Cu(2+) alleviates dextran sulfate sodium-induced ulcerative colitis in mice. Theranostics.

[R328] Zhu G, Xie Y, Wang J, Wang M, Qian Y, Sun Q, Dai Y, Li C (2024). Multifunctional copper-phenolic nanopills achieve comprehensive polyamines depletion to provoke enhanced pyroptosis and cuproptosis for cancer immunotherapy. Adv Mater.

[R329] Zhu MJ, Zhang L, Wang CP (2024). Copper overload promotes β-amyloid induced NLRP3/Caspase-1/GSDMD-mediated pyroptosis in Alzheimer’s disease. J Integr Neurosci.

[R330] Zhu Z, Song M, Ren J, Liang L, Mao G, Chen M (2024). Copper homeostasis and cuproptosis in central nervous system diseases. Cell Death Dis.

[R331] Zimnicka AM, Ivy K, Kaplan JH (2011). Acquisition of dietary copper: a role for anion transporters in intestinal apical copper uptake. Am J Physiol Cell Physiol.

[R332] Zirngibl M, Assinck P, Sizov A, Caprariello AV, Plemel JR (2022). Oligodendrocyte death and myelin loss in the cuprizone model: an updated overview of the intrinsic and extrinsic causes of cuprizone demyelination. Mol Neurodegener.

[R333] Zong WX, Rabinowitz JD, White E (2016). Mitochondria and cancer. Mol Cell.

